# Primary Infection by E. multilocularis Induces Distinct Patterns of Cross Talk between Hepatic Natural Killer T Cells and Regulatory T Cells in Mice

**DOI:** 10.1128/iai.00174-22

**Published:** 2022-07-13

**Authors:** Tural Yarahmadov, Junhua Wang, Daniel Sanchez-Taltavull, Cristian A. Alvarez Rojas, Tess Brodie, Isabel Büchi, Adrian Keogh, Bruno Gottstein, Deborah Stroka, Guido Beldi

**Affiliations:** a Department of Visceral Surgery and Medicine, Inselspital, Bern University Hospital, University of Bern, Bern, Switzerland; b Institute for Infectious Diseases, University of Bern, Bern, Switzerland; c Institute of Parasitology, University of Zurich, Zurich, Switzerland; University of Pennsylvania

**Keywords:** single-cell RNA sequencing, *Echinococcus multilocularis*, NKT cells, single-cell RNA-seq, transcriptomics, Tregs

## Abstract

The larval stage of the helminthic cestode Echinococcus multilocularis can inflict tumor-like hepatic lesions that cause the parasitic disease alveolar echinococcosis in humans, with high mortality in untreated patients. Opportunistic properties of the disease have been established based on the increased incidence in immunocompromised patients and mouse models, indicating that an appropriate adaptive immune response is required for the control of the disease. However, cellular interactions and the kinetics of the local hepatic immune responses during the different stages of infection with E. multilocularis remain unknown. In a mouse model of oral infection that mimics the normal infection route in human patients, the networks of the hepatic immune response were assessed using single-cell RNA sequencing (scRNA-seq) of isolated hepatic CD3^+^ T cells at different infection stages. We observed an early and sustained significant increase in natural killer T (NKT) cells and regulatory T cells (Tregs). Early tumor necrosis factor (TNF)- and integrin-dependent interactions between these two cell types promote the formation of hepatic lesions. At late time points, downregulation of programmed cell death protein 1 (PD-1) and ectonucleoside triphosphate diphosphohydrolase 1 (ENTPD1)-dependent signaling suppress the resolution of parasite-induced pathology. The obtained data provide fresh insight into the adaptive immune responses and local regulatory pathways at different infection stages of E. multilocularis in mice.

## INTRODUCTION

Alveolar echinococcosis (AE) is a rare but lethal zoonotic helminthic disease caused by Echinococcus multilocularis. If untreated, the liver is damaged directly by the parasitic infiltration and mass proliferation and indirectly by the periparasitic host response. In particular, the continuous proliferation of the larval stage (metacestode) of E. multilocularis leads to an intense local granulomatous immune response surrounding the parasitic tissue, thereby promoting tumorlike focal lesions (1, 2).

Clinical observations revealed an elevated incidence of AE in immunocompromised patients, indicating that the incidence and course of the disease may depend on the immune response of the host (3). The increase in case numbers over the last decades can therefore be explained in part by the increased number of patients receiving immunosuppressive therapy, in addition to the increased fox population density and associated environmental contamination with parasite eggs (4–6). Therefore, understanding the immune response to AE is required to apply potential immunomodulatory treatments.

Previous studies on the interaction between the parasite and the host immune system were performed in immunocompromised mice and in humans (7–10). Initial parasite survival and proliferation correlated with a Th2 reorientation of the CD4^+^ T lymphocytes, leading to immunotolerance or to an anergic response to infection at later stages (1, 8, 11, 12). A trend toward resistance to the parasite was associated with Th1-cytokine profiles at later stages, including interferon alpha (IFN-α) (13) and interleukin 12 (IL-12) (14) as initiating cytokines, and IFN-γ (15) and tumor necrosis factor (TNF) alpha (16, 17) as effector cytokines. This results in the persistence of E. multilocularis, which is associated with chronic granulomatous inflammation (18).

These studies are limited, as the most conventional infection model applied resembles secondary AE, i.e., infection induced upon intraperitoneal inoculation of E. multilocularis metacestodes (19, 20). The problem associated with this infection model is that it lacks the first phase of passage of the parasite through the intestinal barrier and the second phase of intrahepatic parasite development, both of which are potentially crucial for the formation of liver lesions (21, 22). Therefore, a primary infection model using orally ingested tapeworm eggs is preferential, as it is representative of the natural route of infection. The few studies using a primary infection model described a systemic humoral response when the metacestode invades the liver (23, 24) and a tolerogenic state at the late stage of AE (20). This model of primary infection is performed in immunocompetent mice in which the infection is ultimately lethal and thereby represents the situation in humans, in which infection frequently occurs in the absence of immunosuppression.

Thus far, no study has reported on the changes of *in situ* host T cell immune responses from the early to chronic stages of primary murine AE. We used single-cell RNA sequencing (scRNA-seq) in a murine model of AE to identify transcriptional changes at the cellular level and to identify alterations in the liver at different stages of the disease. Given that the adaptive immune response drives the disease, we aimed to assess T cell-dependent interactions at the various successive stages of disease, i.e., early, intermediate, and chronic. Our analysis revealed distinct temporal changes with the early expansion of natural killer T (NKT) cells and regulatory T cells (Tregs), as well as changes in their cellular interaction across the time points of the experiment. The activation of specific genes regulates CD4^+^ and CD8^+^ T cells in the early stages and NKT cells and Tregs at later time points.

## RESULTS

### Expansion of NKT cells and Tregs as first responders to infection.

**FIG 1:**
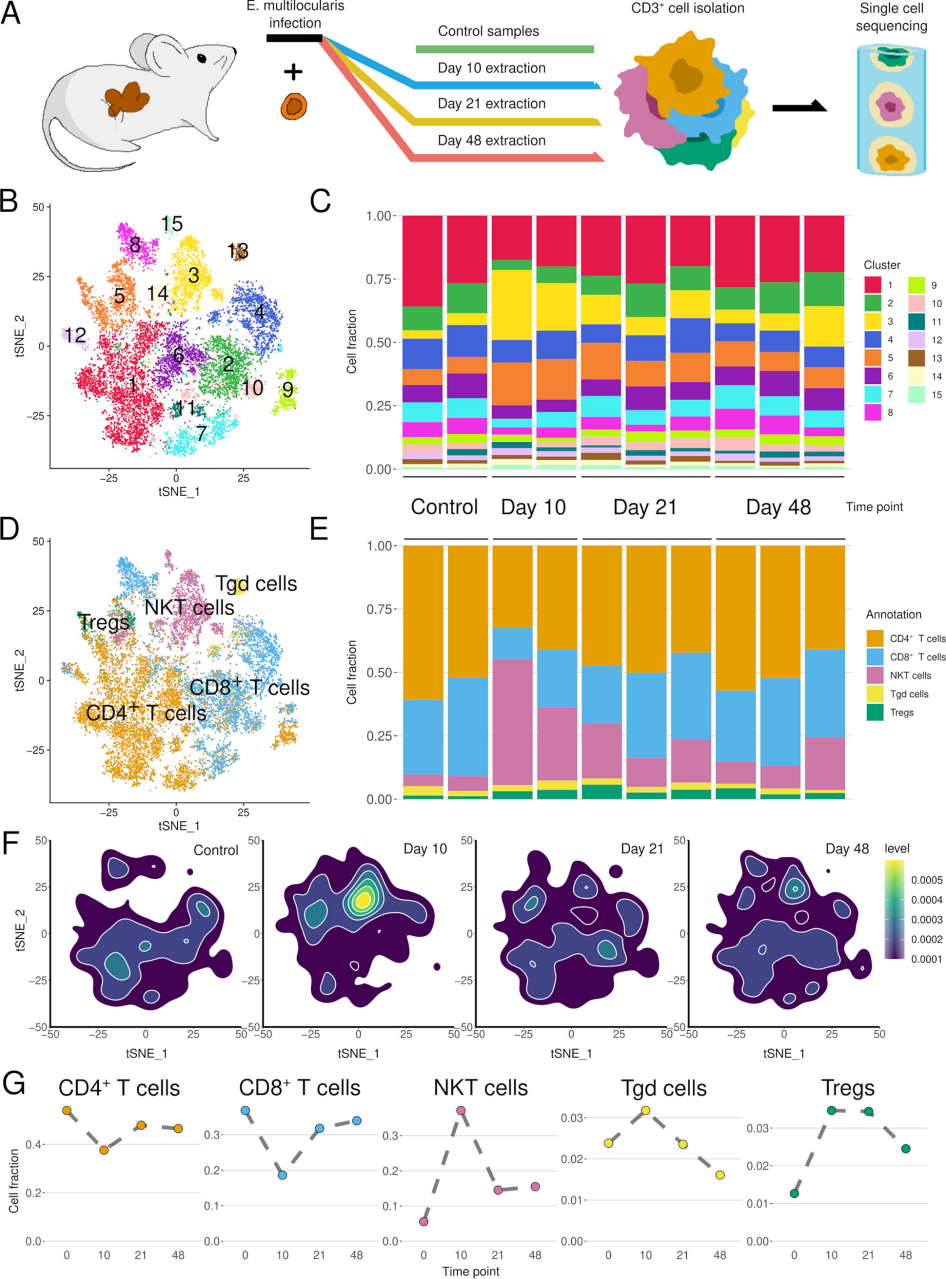
Unsupervised clustering identifies early upregulation of NKT cells and Tregs after oral infection with E. multilocularis. (A) Schematic representation of the experiment. CD3^+^ cells from the livers of control mice and mice at 10, 21, and 48 days post-peroral E. multilocularis infection were extracted for single-cell RNA sequencing. (B) *t*-distributed stochastic neighbor embedding (tSNE) of the final data set based on the single-cell RNA-seq data colored by clusters. (C) Normalized distribution of cell subsets from each cluster in samples is displayed in chronological order from left to right. (D) tSNE of the annotation using SingleR to represent the major T cell types in the data set. (E) Normalized cell numbers (cell subset distributions) from each cell type in chronological order. (F) Density maps of cells at different stages. The highest quantity of NKT cells can be observed at D10 compared to the rest. (G) Distribution of the relative to the total data set number of cells (*y* axis) over the course of the experiment (*x* axis) for all cell types.

We generated and analyzed a murine immune cell data set spanning 4 time points pre- and post-oral infection. The data set consists of hepatic T cells from noninfected control (D0) mice (2 samples) and the cells from the livers of mice at 10 days (D10; early infection, 2 samples), 21 days (D21; intermediate infection, 3 samples), and 48 days (D48; late infection, 3 samples) post-oral infection (Fig. 1A). Together, the studied data set consists of 14,848 cells from 10 different samples. Unsupervised clustering was performed using Seurat and manually adjusted based on a higher clustering resolution level to better separate immune cell subpopulations (see “Dimensionality reduction and filtering” in Materials and Methods) (Fig. 1B and C). The cell types were next annotated using SingleR (25) (Fig. 1D and E; see also Fig. S1 in the supplemental material), resulting in 15 clusters overlaid with the predicted cell annotations showing the spatial separation of the different cell types. According to the SingleR annotation, the final data set consists of 7,057 CD4^+^ T cells (48%), 4,805 CD8^+^ T cells (32%), 2,297 natural killer T (NKT) cells (15%), 372 regulatory T cells (Tregs; 3%), and 317 T gamma delta (Tgd) cells (2%) (Table S1). The differential abundance over the course of the experiment was quantified and tested by fitting the values to a quasi-likelihood negative binomial generalized log-linear model, producing *P* values adjusted using the Benjamini-Hochberg method of controlling the false discovery rate (FDR; for details, see “Differential abundance testing” in Materials and Methods). On D10, the NKT cells expanded sharply (FDR, 0.0004), and to a lesser extent, the abundance of Tregs also increased (FDR, 0.043) (Fig. 1D, F, G). No statistical significance was observed for other cell types or time points.

### Unbiased clustering identifies regulatory networks of early NKT cell and Treg expansion and downregulation of CD4^+^ and CD8^+^ cells.

**FIG 2:**
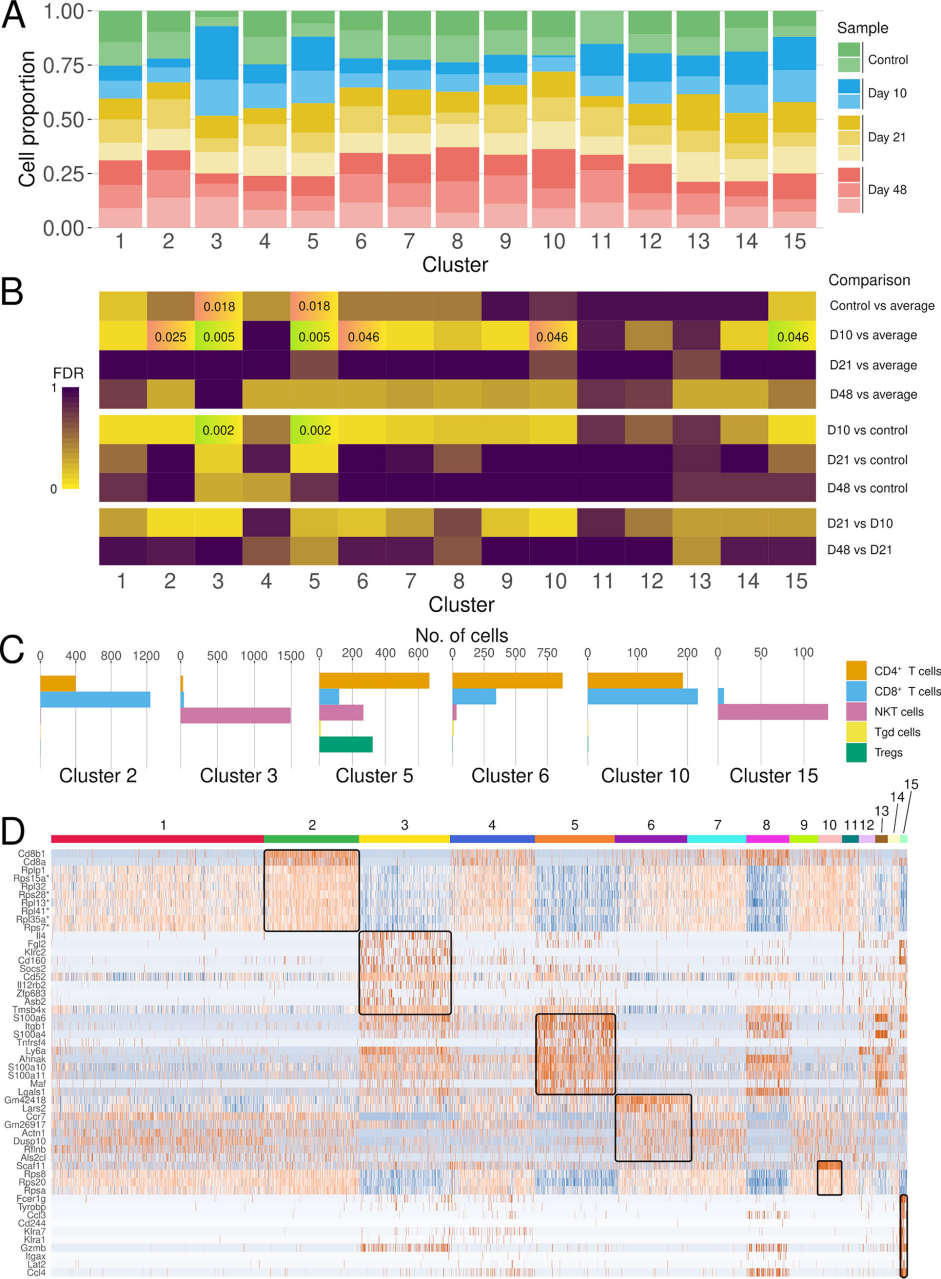
Regulatory networks indicate suppression of CD4^+^ and CD8^+^ T cells and expansion of NKT cells and Tregs on D10. (A) Distribution of sample cell numbers in each cluster over time. The normalized cell numbers from each sample are displayed per cluster. (B) Results of differential abundance testing of the number of cells in each cluster compared to the average number across other time points. Cells with FDR values of <0.05 are labeled and tinted green for higher numbers and red for lower numbers. (C) The majority of NKT cells and Tregs are contained in overrepresented D10 clusters. Underrepresented clusters contain only CD4^+^ and CD8^+^ cells. (D) Heat map of the most differentially expressed marker genes from the clusters of interest. The 10 most significant markers by *P* value and log_2_-fold change per cluster are depicted. Each line represents the expression value in one cell. Expression values are Z-score transformed by row, with low values shown in blue, intermediate in white, and high values in red. Genes and cells from differentially abundant clusters are highlighted via rectangles. *, markers that are shared between clusters 2 and 10.

Next, differential abundance testing of cellular subpopulations was performed for an unbiased assessment of the assembly and dynamics of the transcriptome of the cellular subsets (Fig. 2A). The cell numbers at each time point were compared to the control samples only and to the average of the other time points, and consecutive time point-wise (Fig. 2B). Significant changes were found for clusters 2, 3, 5, 6, 10, and 15 on D10. Clusters 3 and 15, which were elevated on D10, consist predominantly of NKT cells, while cluster 5 contains the majority of Tregs. Clusters 2, 6, and 10 decreased on D10; they represent CD4^+^ and CD8^+^ cells on D10 post-oral infection with E. multilocularis eggs, as shown in Fig. 1G (Fig. 2C; Table S1). The resolution did not allow us to identify further subsets of CD4^+^ and CD8^+^ cells.

The majority of all NKT cells in the data set are represented in cluster 3 (1,491/2,297 cells). Both clusters 3 and 15 consist almost entirely of NKT cells (1,491/1,573, 95% and 128/135, 95%) (Fig. 2C). This observation is further supported by the expression of, e.g., Zfp683/Hobit in this cluster, which is a gene important for proper NKT cell development and morphology (26) (Fig. 2D). Cluster 15 exhibits markers characteristic of both NKT and NK cells, indicating some potential remaining subsets of NK cells in the data set despite negative selection and filtering.

The majority of Tregs in the data set are represented in cluster 5. Its clustering is dependent by the presence of S100 genes (Table S2) and includes heterogenous cell types, split between 667 CD4^+^ T cells (48%), 323 Tregs (23%), 267 NKT cells (19%), and 125 CD8^+^ T cells (9%) (Fig. 2B and C; Table S1).

CD8^+^ T and CD4^+^ T cells are represented in clusters 2, 6, and 10. Cluster 2 is split between 1,243 CD8^+^ T cells (75%) and 398 CD4^+^ T cells (24%) (Fig. 2B). It displays high expression of CD8B1, a cell surface glycoprotein characteristic of cytotoxic CD8^+^ T lymphocytes (27). Cluster 6 contains 871 CD4^+^ T cells (69%) and 345 CD8^+^ T cells (27%) (Fig. 2B) and is characterized by high expression of the long noncoding RNAs Gm42418 and Gm26917 (Fig. 2D). Cluster 10 is evenly divided between 221 CD8^+^ T cells (53%) and 191 CD4^+^ T cells (46%) (Fig. 2B). This cluster is also remarkable for its strong and almost exclusive caspase 11 (Scaf11) expression (Fig. S3).

The two clusters, 2 and 10, underrepresented on D10 are among the few remarkable for the strong expression of genes encoding ribosomal proteins, indicating an active downregulation of the associated CD4^+^ and CD8^+^ T cells (Fig. S4).

These unbiased data indicate predominant changes in the specific cell fractions at an early time point of the disease, with an elevation of NKT cells and Tregs and active suppression of CD4^+^ and CD8^+^ cells.

### Interactions between NKT cells and Tregs mediate early recruitment and regulation of the responses at late phases.

**FIG 3:**
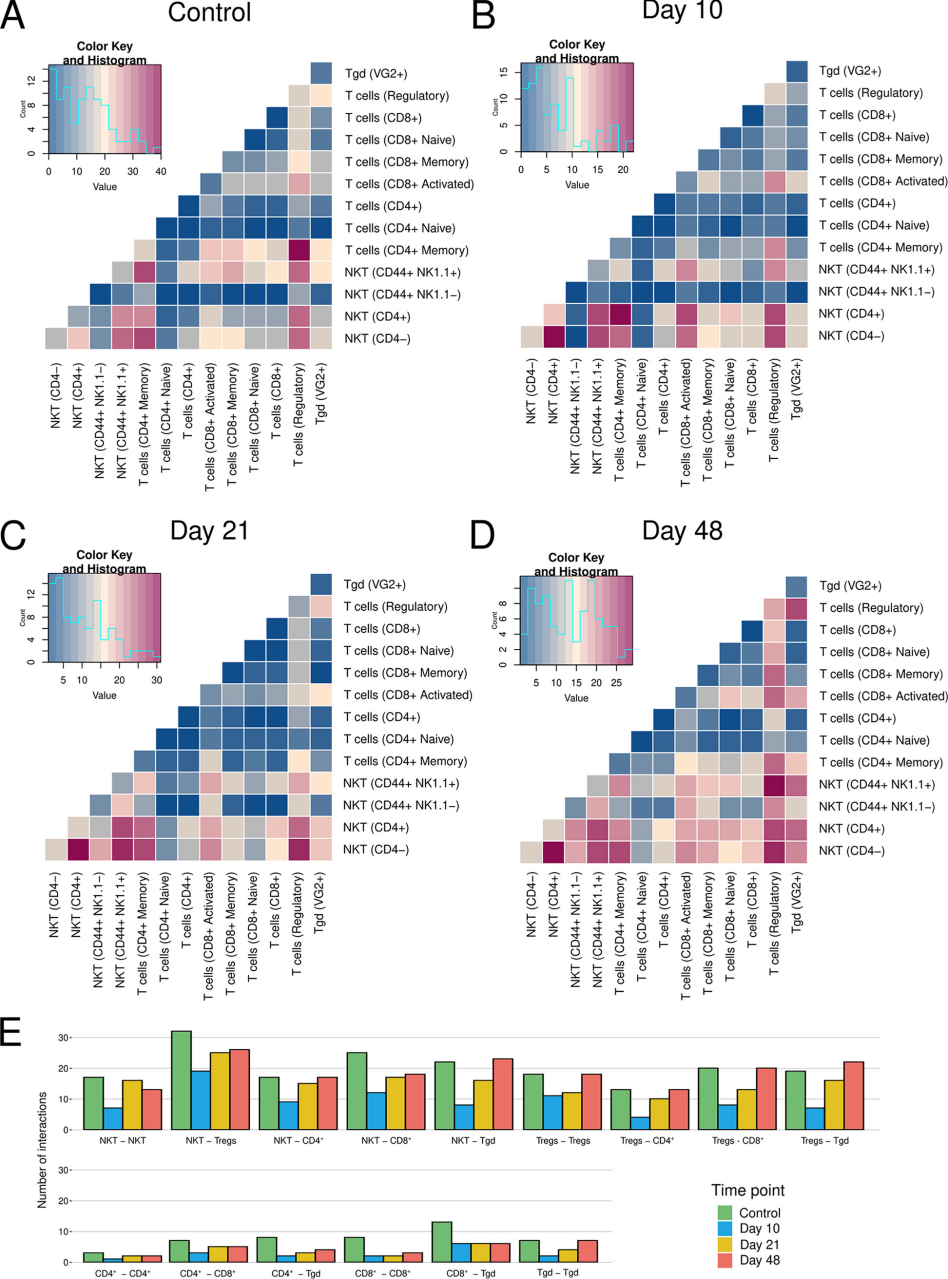
Maps of intercellular interactions showing a consistently high number of NKT and regulatory T cell interactions over the course of the experiment. Heat maps summarizing the statistically significant ligand-receptor interactions per cell type pair for the control (A), D10 (B), D21 (C), and D48 samples (D). (E) Total number of significant interactions between pairs of cell types, indicating a decrease on D10.

After identifying the genes (Fig. 2D) associated with the expansion and activation of NKT cells and Tregs on D10, we aimed to analyze the cross talk between the different immune cell types and subtypes (Fig. S5). We performed a receptor-ligand complex analysis via CellPhoneDB, using mouse orthologs from ENSEMBL to access the human database (Fig. 3) (28, 29). A consistently high number of interactions between Tregs and NKT cells was observed, while CD4^+^ T and CD8^+^ T cells showed a low number of predicted interactions between themselves and each other (Fig. 3A to E; Fig. S6). The CD4^+^ T and CD8^+^ T interaction pairs also displayed a reduction in number on D10, with a subsequent return to the baseline number of connections to other cell types over the course of the experiment (Fig. 3E). These kinetics were also observed in the interaction between NKT cells and Tregs, both expanding on D10 and associated with a transient suppression of cellular interactions at this time point (Fig. 1G). These results indicate that NKT- and Treg-driven interactions dictate the early course of infection, while CD4^+^ T and CD8^+^ T cells are suppressed at this time point.

### TNF-dependent interactions between NKT cells and Tregs drive early responses.

**FIG 4:**
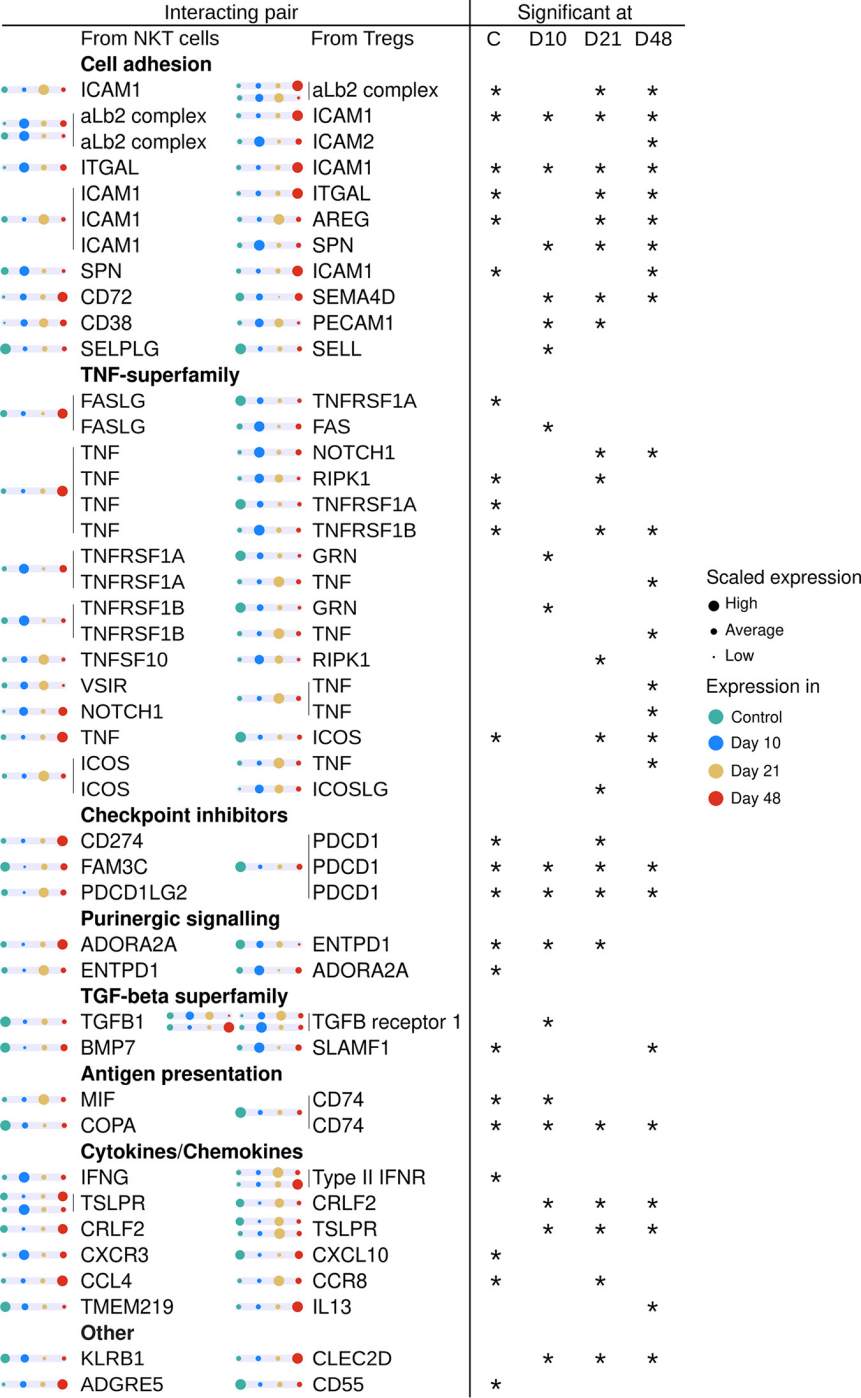
Temporal changes in significant interactions between NKT cells and Tregs. Mouse genes expressed by NKT cells and Tregs were associated with human orthologs and then analyzed using CellPhoneDB v.2.0.0 at each of the time points in order to predict the statistically significant pairwise interactions on a protein level. The pairs are represented by their participating orthologous gene names on the left, along with scaled expression levels per cell type across the experiment. Significant interactions (adjusted *P* value, <0.05) at different time points are shown as stars in their respective columns. For interacting protein complexes, the participating encoding genes are grouped together.

While the number of predicted interactions between Tregs and NKT cells remained comparatively high throughout the course of the infection (Fig. 3A to D), the composition of the interacting pairs changed across the time points (Fig. 3E, Fig. 4). NKT cell recruitment depended on the upregulation of the alb2 complex (LFA-1) and upregulation of ICAM1 on D10 that occurred in parallel to the upregulation of the selectin-dependent interaction SELPLG-SELL. In parallel to the recruitment, immune-suppressing interactions in NKT cells, such as checkpoint inhibitors (PD-1 and PD-L1), purinergic (ENTPD1 and ADORA2A), and transforming growth factor beta (TGF-β) signaling, were downregulated on D10 and D21.

In order to evaluate the connectivity between the proteins forming the NKT cell and Treg interaction pairs at the control and D10 time points, their protein-protein interaction (PPI) networks were constructed using the STRING database interface (Fig. S8). All resulting networks were found to be strongly interconnected, with the number of connections being significantly higher than expected (PPI enrichment *P* value, <1.0e−16). The networks made from proteins forming pairs in the control samples had a higher average degree than those made from D10 proteins in both NKT cells (7.65 to 3.89) and Tregs (7.85 to 4.95).

These data indicate that the recruitment and activation of NKT cells depends on integrins, selectins, and TNF-dependent signaling in parallel with the early downregulation of immunosuppression-dominating pathways. At the later stages of the experiment, immune suppression allowed for parasite survival.

## DISCUSSION

Based on studies in mice and humans, adaptive T cell-mediated responses dictate the outcome after infection respective to the development of chronic AE (10). In the present study, we investigated the *in situ* hepatic T cell responses involved in immune cell homing at the various successive stages of disease (early, middle, and chronic) in a model of primary murine AE. By performing scRNA-seq of isolated CD3^+^ T cells, we showed a significant increase in NKT cells, and to a lesser extent Tregs, on D10 postinfection, with a subsequent decrease at later time points (D21 to D48) to the levels of noninfected controls (Fig. 1). In parallel, CD4^+^ and CD8^+^ T cells were downregulated in a Caspase-11-dependent manner on D10. This indicates a peak in the acute immune response to E. multilocularis infection around 10 days post-oral infection, with a subsequent stabilization once the lesions were grossly visible in the liver (at 14 days post-oral infection in mice; not shown).

Unbiased analysis of the transcriptional data revealed 6 subclusters with significant differences in abundance over time (Fig. 2). Consistent with alterations in the cellular subsets (Fig. 1), the differences were predominant on D10, with the overrepresentation of two NKT cell clusters (Fig. 3A and B), which supports the role of NKT cells found in other macroparasitic infections (30, 31). A gene important for proper NKT cell development and morphology, Zfp683/Hobit (26) is a strong marker for cluster 3, thus confirming the results of the automatic annotation of the individual cell subsets (Fig. 2C). Our data are in contrast with previous findings which suggested that NKT cells played a rather minor role against infection with E. multilocularis (18). However, these previous data were derived from a model of secondary infection, with intraperitoneal administration of the parasite, in which intestinal uptake and hepatic seeding of AE were bypassed and therefore could not be assessed. Our current study indicates that NKT cells drive the response during the early infection stage of primary AE, potentially through interactions with Tregs (Fig. 3C and D).

The finding that NKT cells expand in patients with AE (32) supports the finding of the present study. Given the fact that many microbes and parasites are enriched in lipid antigens and NKT cells are a T cell subset that can recognize lipid antigens, it is reasonable to speculate about the potential relevance of NKT cells to control the early development of infection and control of the disease at later stages. The effector mechanisms by which NKT cells respond to E. multilocularis primarily consist of signaling by IFN-γ, TNF, IL-7, and IL-18 and cytotoxicity pathways, including granzyme b and killer cell lectin receptors (see Fig. S7 and Table S3 in the supplemental material).

In contrast to the role of NKT cells, Tregs have been shown to be relevant for the control of AE in patients (33) and in experimental models of primary AE (20, 34, 35). Previous research and our data suggest that metacestode persistence may be the consequence of immune tolerance or anergy, respectively, mainly mediated by specialized Tregs and related cytokines, such as IL-10 and TGF-β (Table S4) (20). The effect of NKT cells may be similar to the effect of Tregs that are manipulated by parasites (36), leading to an induction of inhibitory cytokines and/or chemokines, allowing worms to survive in their mammalian hosts for many years.

At the early time point, CD4^+^ and CD8^+^ were strongly downregulated. A peculiar subset of downregulated CD4^+^ and CD8^+^ cells expressed caspase-11 (Scaf11; cluster 10) (Fig. 2C; Fig. S3), indicating associated pyroptosis and cell death (37, 38), potentially by gasdermin-dependent regulation of these cells at an early stage of infection, as found in other parasitic diseases (39, 40). In addition, the CD8^+^ T cell-dependent cytotoxicity on D10 was actively suppressed, as indicated by the decrease in cell numbers and the high ribosomal gene activity, indicating translational activity of the cells (Fig. 2C; Fig. S4) (27). Also, the underrepresented CD4^+^/CD8^+^ cell cluster 6 was characterized by high expression of the long noncoding RNAs Gm42418 and Gm26917, which were found to be enriched in NLRP3 inflammasomes, which trigger inflammatory cell death and the release of cytokines (41, 42). These changes indicate that the parasite initiates active CD4^+^ and CD8^+^ T cell suppression in addition to NKT cell activation, which is in favor of parasite survival.

Analysis of the interaction between NKT cells and Tregs indicates the relevance of the activation of the TNF superfamily of integrin and selectin-dependent recruitment of NKT cells at the early time point (Fig. 4). This may be paralleled with the TNF production seen in NK cells (32). While clinical evidence of the opportunistic properties of AE is growing (6), the relevance of TNF signaling has been suggested once (43).

Conversely to the TNF-dependent activation, the genes associated with immune suppression include checkpoint blockade (PD-1/PD-L1) and purinergic signaling (ENTPD1/ADORA2A), which are upregulated during the early phase and downregulated during the late phase. The downregulation of these genes at the later time points indicates that the parasite seems to control the local immune system. Potentially, such an active checkpoint inhibition might be the cause of the altered immune reaction, with dense fibrous walls around the parasite, as observed in mice and humans, aiming to limit parasite growth.

Together, these comprehensive findings on the immunological changes at different stages in primary E. multilocularis infection now open the door for more applied approaches. For instance, addressing the role of CD4^+^ and CD8^+^ T cells by modulating pyroptotic pathways or by addressing the role of TNF signaling, checkpoint blockade, or purinergic signaling to modulate NKT cell responses could provide tools for converting the immunological anergy during chronic disease into a more proinflammatory response, with potentially fatal consequences for the metacestode. Furthermore, our findings may provide a rationale for studying immune cells as a target for an immunomodulatory treatment option in patients with progressive AE.

## MATERIALS AND METHODS

**Ethics statement.** The animal studies were performed in accordance with the recommendations of the Swiss Guidelines for the Care and Use of Laboratory Animals. The protocol was approved by the Governmental Commission for Animal Experimentation of the Canton of Bern (approval no. BE112/17).

**(i) Mice.** Thirty-six female 8-week-old wild-type C57BL/6-mice were purchased from Charles River GmbH (Sulzfeld, Germany) and divided randomly into 2 groups, (i) E. multilocularis infected (“AE”) and (ii) noninfected control (“control”), at three time points (D10, D21, and D48 post-oral infection; 6 mice per group/time point).

**(ii) Parasite and experimental infection.** E. multilocularis eggs were isolated from a naturally E. multilocularis-infected fox, which was euthanized at the Institute of Parasitology, Vetsuisse Faculty, Zurich. Infection with E. multilocularis was detected upon routine necropsy investigation by pathologists. To prepare the parasite eggs for the subsequent infection of mice, the fox intestine was removed under appropriate safety precautions and cut into 4 pieces. After longitudinal opening of the intestinal segments, the worm-containing mucus was scraped out and put into petri dishes containing sterile water. Subsequently, the mucosal suspension was serially filtered through 500-μm and then 250-μm metal sieves, by concurrently disrupting the worms with an inversed 2-mL syringe top. This suspension was further filtered through a 105-μm nylon sieve. The eggs were then washed by repeated sedimentation (1 × *g*, 30 min, room temperature) in sterile water containing 1% penicillin/streptomycin and stored in the same solution at 4°C. For the primary infection of the mice, the animals received approximately 400 eggs suspended in 100 μL sterile water by peroral gavage. The control mice (mock-infection) received 100 μL water only. All animal infections were performed in a biosafety level 2 plus unit (permit no. VTHa-R9).

**(iii) Sampling.** On D10, D21, and D48 postinfection, the mice were sacrificed by CO_2_ euthanasia. At necropsy, the number and size (diameter) of the individual liver lesions (each caused by one developing oncosphere, originating from one parasite egg) were recorded. Liver cells were isolated from 3 AE mice/time point with successful liver lesions by using Percoll density gradient centrifugation (see below).

### Hepatic lymphocyte isolation.

The mouse livers were cut into small pieces with a scalpel and crushed against a 50-mm stainless steel mesh plate. The mesh plate was rinsed with cold MACS buffer (phosphate-buffered saline [PBS] with 0.6% fetal bovine serum [FBS] and 0.5 M EDTA) to release the cells into the collection plate. The cells were washed with MACS buffer and centrifuged at 50 × *g* for 3 min at 4°C to get rid of most of the hepatocytes, and the supernatant was collected (nonparenchymal cells). The nonparenchymal cells were then centrifuged at 300 × *g* for 7 min at 4°C. The pellet was resuspended in 10 mL of 40% Percoll and overlaid with 70% Percoll. The cells were centrifuged at 800 × *g* for 20 min at 4°C with no brake, and the interphase cells were collected and resuspended in 15 mL MACS buffer. The cells were centrifuged at 300 × *g* for 7 min at 4°C; the cell pellet was resuspended and then counted using the Bio-Rad cell counter (TC20).

### CD3 negative selection.

Nonparenchymal cells (0.25 to 0.5 million) were used with the EasySep mouse T cell isolation kit (Stemcell Technologies, catalog no. 19851) for the negative selection of T cells, following the kit protocol. Briefly, cells were stained with a cocktail of antibodies bound to magnetic particles and specific for non-T cells. These cells were retained against the wall of the fluorescence-activated cell sorting (FACS) tube when the EasySep magnet was applied, and the remaining cells (T cells) were poured out of the tube and counted for sequencing.

### Sequencing.

Between 5,000 and 20,000 cells per sample were provided to the next-generation sequencing platform at the University of Bern, and standard methods were used to perform the sequencing. The read and data processing steps were performed with the help of UBELIX (http://www.id.unibe.ch/hpc), the high-performance computing (HPC) cluster at the University of Bern.

### Read processing.

Feature barcode counting was performed using the 10x Genomics Cell Ranger v.3.0.2 count function with the software-provided mouse reference genome mm10-3.0.0 and the *–expect-cells* parameter value set to 5000.

### Data processing.

The obtained data were analyzed in R v.3.6.1 (44). The data were imported into R and analyzed using the Seurat R package v.3.1.4 (45). Filtered count matrices produced by the Cell Ranger count function per sample were imported into R as Seurat objects using the Read10X function. Cells in the samples were individually filtered based on the gene (feature) content and proportion of mitochondrial mRNA using the Seurat subset command. For sample C1D10, the threshold was to have at least 100 genes; for sample AE2D10, 150 genes; for sample AE3D10, 200 genes; for samples AE1D48, AE2D48, C1D21, and C1D48, 250 genes each; and for samples AE5D21, AE6D21, AE7D21, and AE3D48, 300 genes. All samples were filtered to have cells with at most 15% of mitochondrial RNA. All samples were combined using the Seurat merge function. Samples AE3D10 and C1D10 were excluded based on the initial clustering analysis due to abnormal clustering and high mitochondrial content. The remaining samples were merged into a single object by applying Seurat SCTransform based on 3,000 anchoring features (with the parameter *vars.to.regress = “percent.mt”*).

### Cell annotation and filtering.

The data were analyzed with seed 322. The cells were annotated using the Immunological Genome Project (ImmGen) database (accessed February 2020) (46) with the R package SingleR v.3.10 (25), “main” and “fine” annotations. Only cells related to CD3^+^ T cell annotations—T cells, Tgd, or NKT cells—were kept for further analysis (15,228 retained out of 16,327; 6% discarded). CD4^+^ and CD8^+^ T cells were distinguished based on fine-level annotations.

### Dimensionality reduction and clustering.

Principal-component analysis (PCA) was performed on the full postfiltered data set using the Seurat function RunPCA. A tSNE dimension reduction was performed using the Seurat FindNeighbors and RunTSNE functions with the parameter *dims = 1:24*, which was chosen based on the ElbowPlot function graph. Clusters were defined via the FindClusters function at resolution 0.3.

### Data filtering and reclustering.

Positive cluster markers were identified using the FindAllMarkers function with the parameters *only.pos = T*, *min.pct = 0.25*, *logfc.threshold = 0.25*. The cell cycle stage of the cells was assessed using the function CellCycleScoring, with s.features and g2m.features provided by Seurat in the R object cc.genes after being transformed into mice genes with the R function gorth of the R package gprofiler (47). Based on a marker and cell cycle analysis, clusters 14 and 16 of likely actively proliferating cells were discarded. The remaining cells (14,848 retained out of 15,228; 10% discarded) were reclustered with the parameter *dims = 1:20* at resolutions 0.3 and 0.9. Clustering at resolution 0.3 with 14 cell groups was selected as a base for further study. Based on SingleR annotations, cluster 15 from resolution 0.9 was reintroduced into the final data set as cluster 15, resulting in 15 final cell groups (see Fig. S9 in the supplemental material). The clusters were finally sorted by size and renamed starting with 1.

### Data analysis.

For marker analysis, the raw expression data (“RNA” assay) were normalized using the Seurat function NormalizeData with the parameters *normalization.method = “LogNormalize”* and *scale.factor = 10000*. Afterwards, the data were scaled for all genes using the Seurat ScaleData function with default parameters. Cluster markers of the final data set were then identified using the Seurat FindAllMarkers function as described above. Gene Ontology (GO) term enrichment analysis of the statistically significant (adjusted *P* value, <0.05) cluster markers was performed with the help of the R package clusterProfiler and Metascape (48, 49). The compareCluster function was used to compare the marker sets per cluster to the org.Mm.eg.db database v.3.10 (50) with the parameters *fun =“enrichGO*,” *ont = “BP*,*” pAdjustMethod = “BH*,*” pvalueCutoff = 0.05*, *qvalueCutoff = 0.10*, *OrgDb = “org.Mm.eg.db*,*” keyType = “SYMBOL*.”

### Differential abundance testing.

In order to find clusters of interest, differential abundance testing was performed as described in chapter 14 of reference 51 using the R package edgeR (52). Input for the described workflow was produced as follows: cells were assigned a “day” parameter based on the stage of the experiment at which they were harvested. An abundance matrix of the cell counts per sample (columns) per cluster (rows) was produced and used to create a DGEList function output object. A design matrix was produced by the model.matrix function from the sample IDs (identifications) using the “day” parameter as a blocking factor. The clusters to be significantly increased on D10 were clusters 3 (adjusted *P* value, 0.005), 5 (adjusted *P* value, 0.005), and 15 (adjusted *P* value, 0.046) and those to be decreased were clusters 2 (adjusted *P* value, 0.025), 6 (adjusted *P* value, 0.046), and 10 (adjusted *P* value, 0.046). Clusters 3 and 5 were also found to have a significantly lower fraction of control samples compared to the average of the others (adjusted *P* values, 0.018 and 0.018, respectively), as well as to just the D10 samples (adjusted *P* values, 0.002 and 0.002, respectively) (Fig. 2B).

### Inference of pairwise cell interactions.

In order to predict the pairwise immune cell type interactions, expression matrices of the annotated cell types were extracted from each time point of the experimental data. The gene expression data were transformed using the formula *x*/[sum(*x*) *×* 10,000]. The data were then analyzed according to the standard CellPhoneDB v.2.1.7 pipeline using the cellphonedb method, statistical_analysis mode with default parameters against the v.2.0.0 database (28). To match the database, genes were preliminarily renamed to human homologs via the R biomaRt v.2.48.2 package getLDS function, accessing the ENSEMBL Homo sapiens and Mus musculus databases (53). For visualization, if multiple mouse orthologs were associated with a single human gene, an ortholog with the highest average expression was used. Protein-protein interaction networks of genes forming significant pairs were built and analyzed using the Search Tool for the Retrieval of Interacting Genes/Proteins (STRING) database v.11.5 (54) interface (https://string-db.org).

### Data visualization.

tSNE scatterplots were constructed using the Seurat function DimPlot with the parameters *reduction = “tsne”* and *pt.size = 0.2*. Gene expression violin plots were constructed using the Seurat VlnPlot function with the parameter *pt.size = 0*. Cell type density plots, stack plots, and dot plots were constructed with the help of the R package ggplot2 v.3.3.0 (55). Density plots were built using the tSNE coordinates of each cell per time point with the help of the stat_density_2d function with the parameters *geom = “polygon*,*” bins = 7*, *na.rm = T*. Enrichment assay visualization was performed using the dotplot function from the clusterProfiler package with default parameters. Cell composition bar plots were created using Microsoft Excel 2019. Gene expression dot plots were created using the Seurat R package DotPlot function with default parameters. Images were edited using Inkscape v.0.92.3 (56).

### Data availability.

scRNA-seq data are available on Gene Expression Omnibus repository with accession number GSE207480.
