# Supplementary material for: Primary Infection by E. multilocularis Induces Distinct Patterns of Cross Talk between Hepatic Natural Killer T Cells and Regulatory T Cells in Mice
Source: Infect Immun. 2022 Jul 13;90(8):e00174-22. doi: 10.1128/iai.00174-22 (PMC9387288; doi:10.1128/iai.00174-22)
Supplement: Supplemental file 1 — Supplemental material. Download iai.00174-22-s0001.pdf, PDF file, 2.1 MB [file iai.00174-22-s0001.pdf]

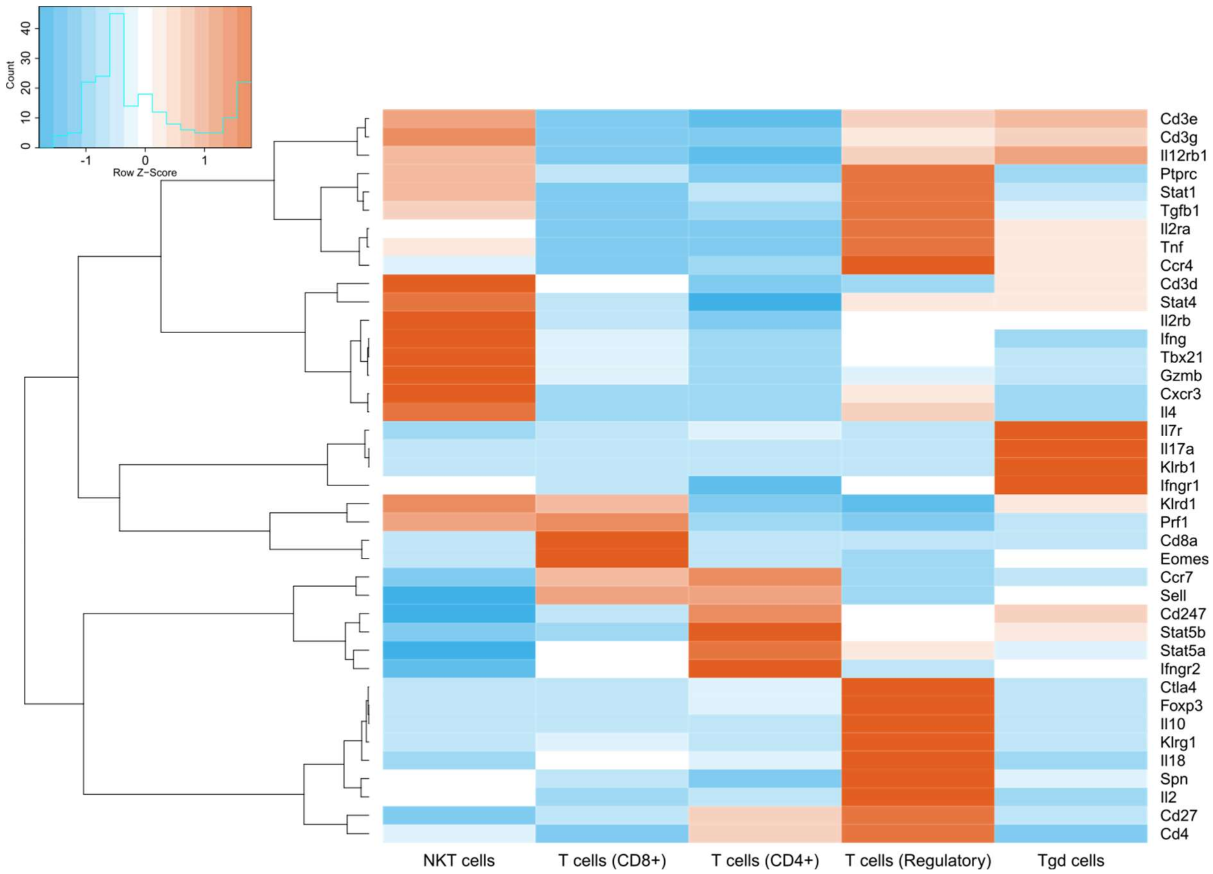

Figure S1. SingleR annotation of the dataset is supported by cell type-defining marker expression. For each SingleR-annotated cell type, the average expression of manually selected cell markers is shown.

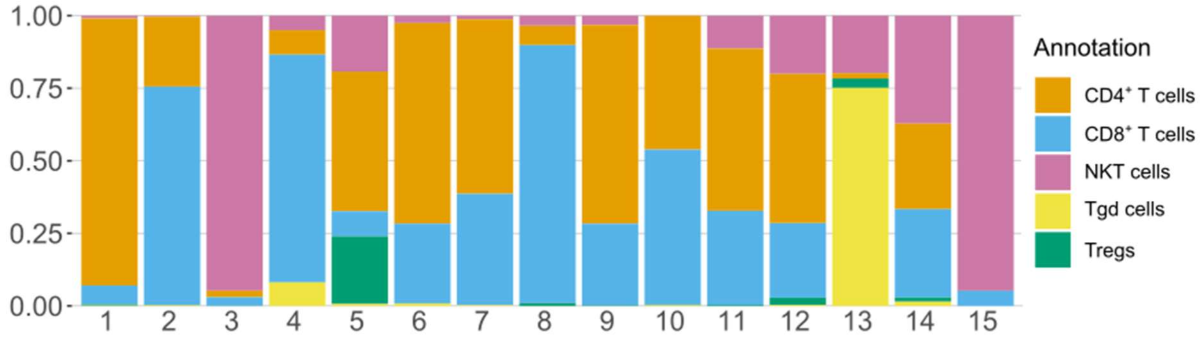

Figure S2. Majority of dataset's NKT cells is contained within clusters 3 and 15, while most of the Tregs are in cluster 5. For each cluster, the proportion of different cell types is shown.

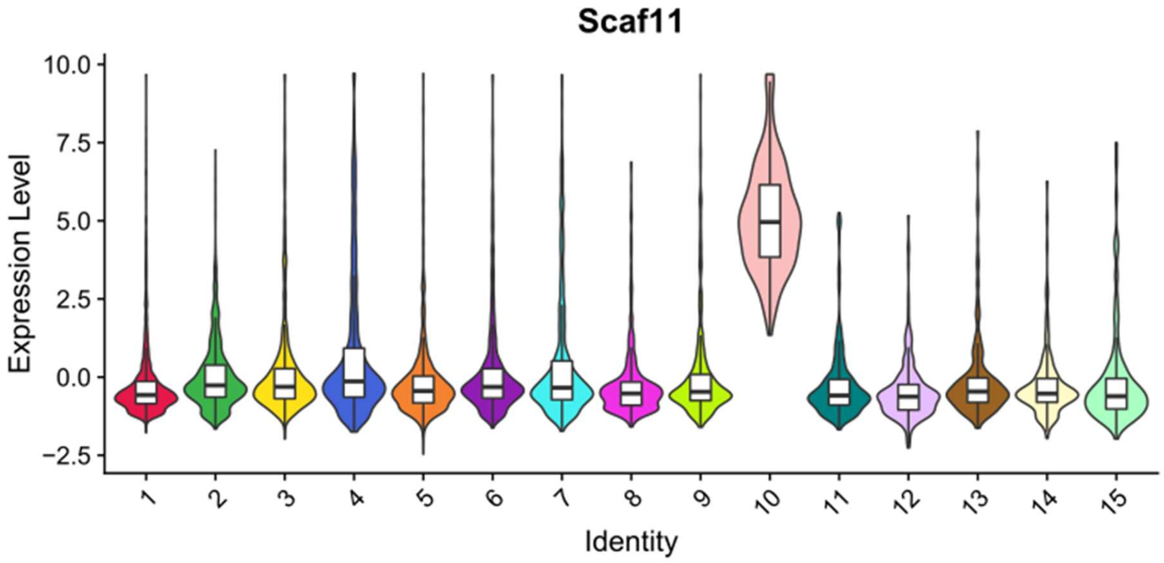

Figure S3. Scaf11 is a strong marker of CD4+/CD8+ cell cluster 10. Violin plots of expression of Scaf11 in each of the clusters of the datasets are shown, along with a boxplot showing the median and upper/lower quartiles.

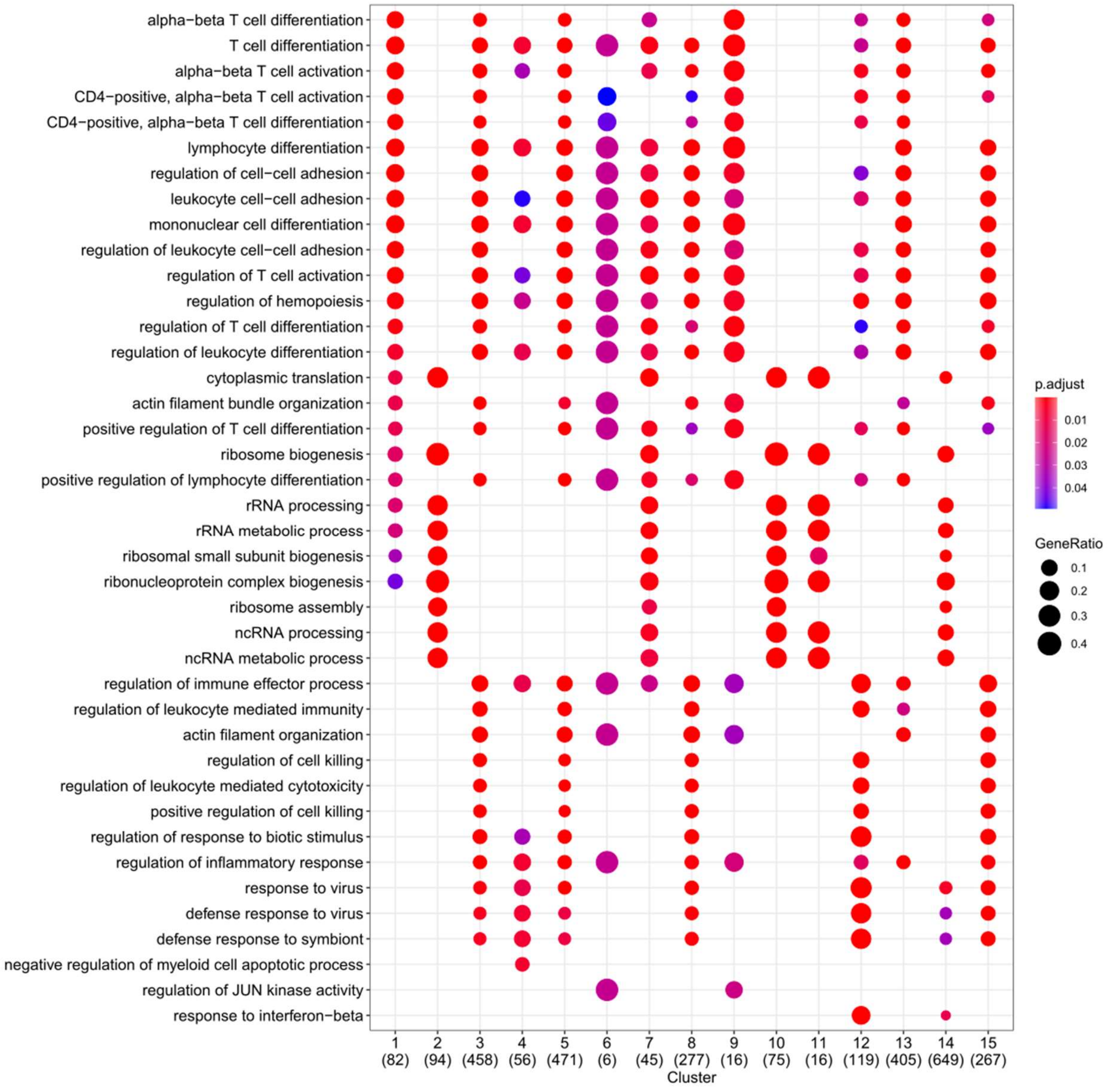

Figure S4. The results of marker set enrichment assay highlight few clusters with ribosomal gene expression on a background of majority immune response-related sets. Enriched pathways are shown on the left, analysed clusters with the number of significant markers (p-value adjusted < 0.05) in brackets on bottom.

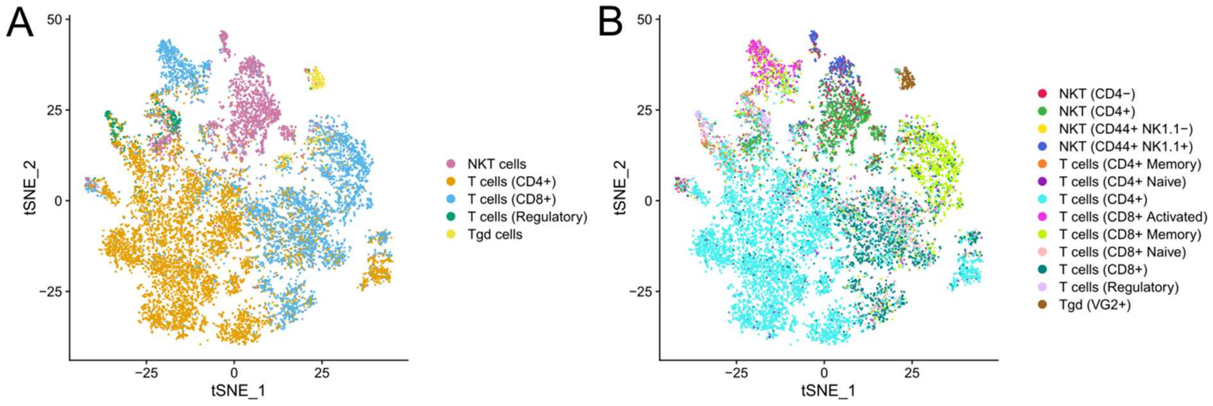

Figure S5. Distribution of T cell subtypes in the experiment. SingleR-based generic cell types (**A**) as well as advanced annotations (**B**) are shown on the tSNE plots representing the data. For exact numbers see **Table S1**.

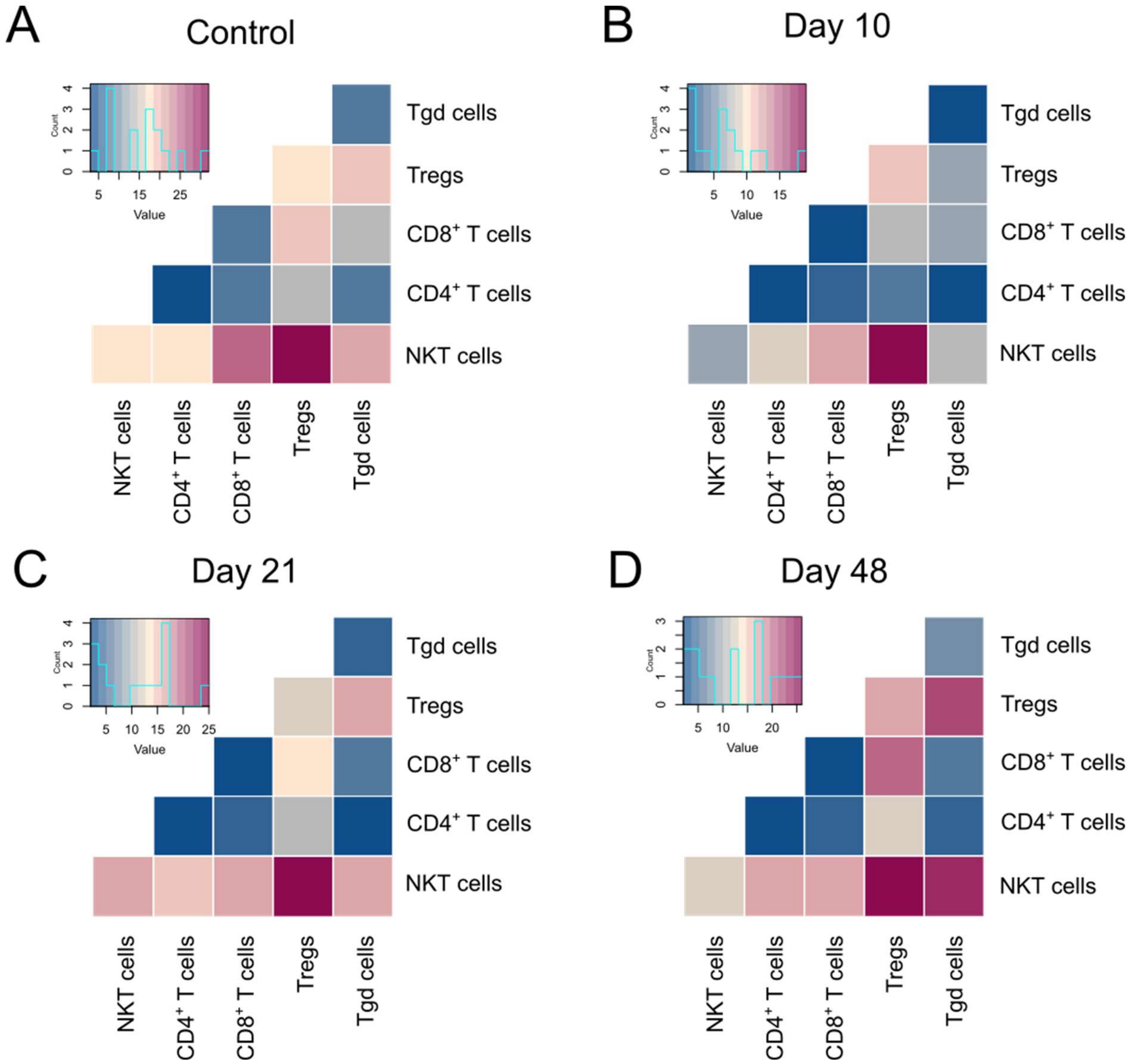

Figure S6. Maps of intercell interactions show a consistently high number of NKT cell and regulatory T cell interactions over the course of the experiment. Heatmaps summarizing statistically significant ligand-receptor interactions per general cell type pair can be seen for control (**A**), D10 (**B**), D21 (**C**), and D48 samples (**D**).

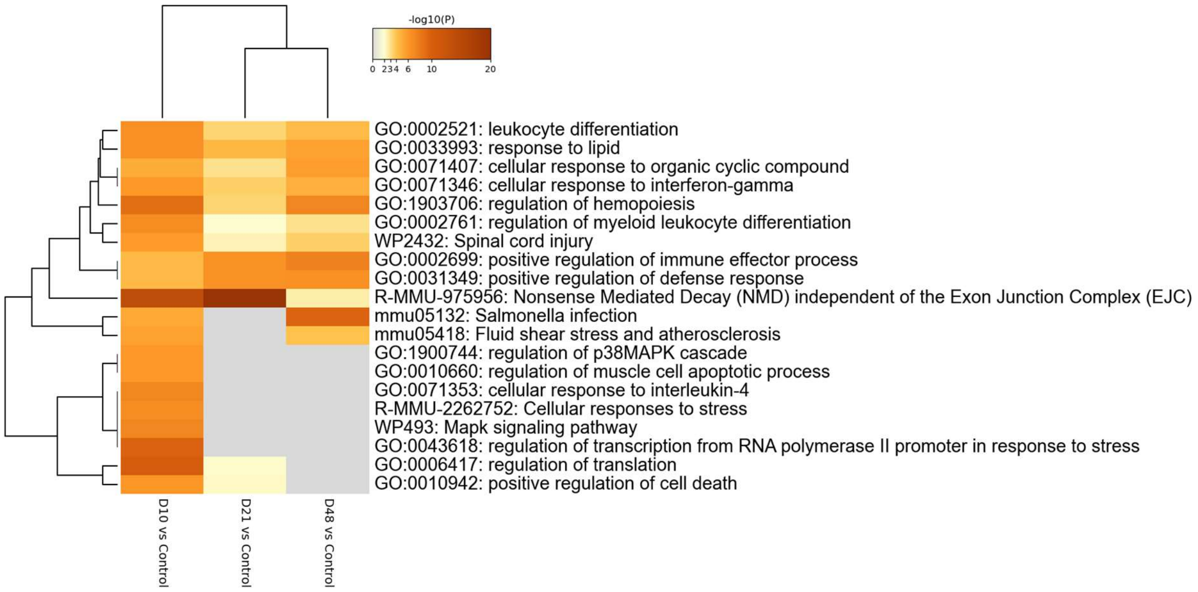

Figure S7. GO term enrichment assay of NKT cell markers of D10, D21 and D48 compared to the control cells shows effector pathways that regulate the response to the *E.multilocularis* infection. Heatmap was generated by supplying significant (p-value adjusted < 0.05) NKT cell markers of D10, D21, and D48 vs control comparisons (Table S3) to the Metascape web interface with default parameters (metascape.org, database version 20220422).

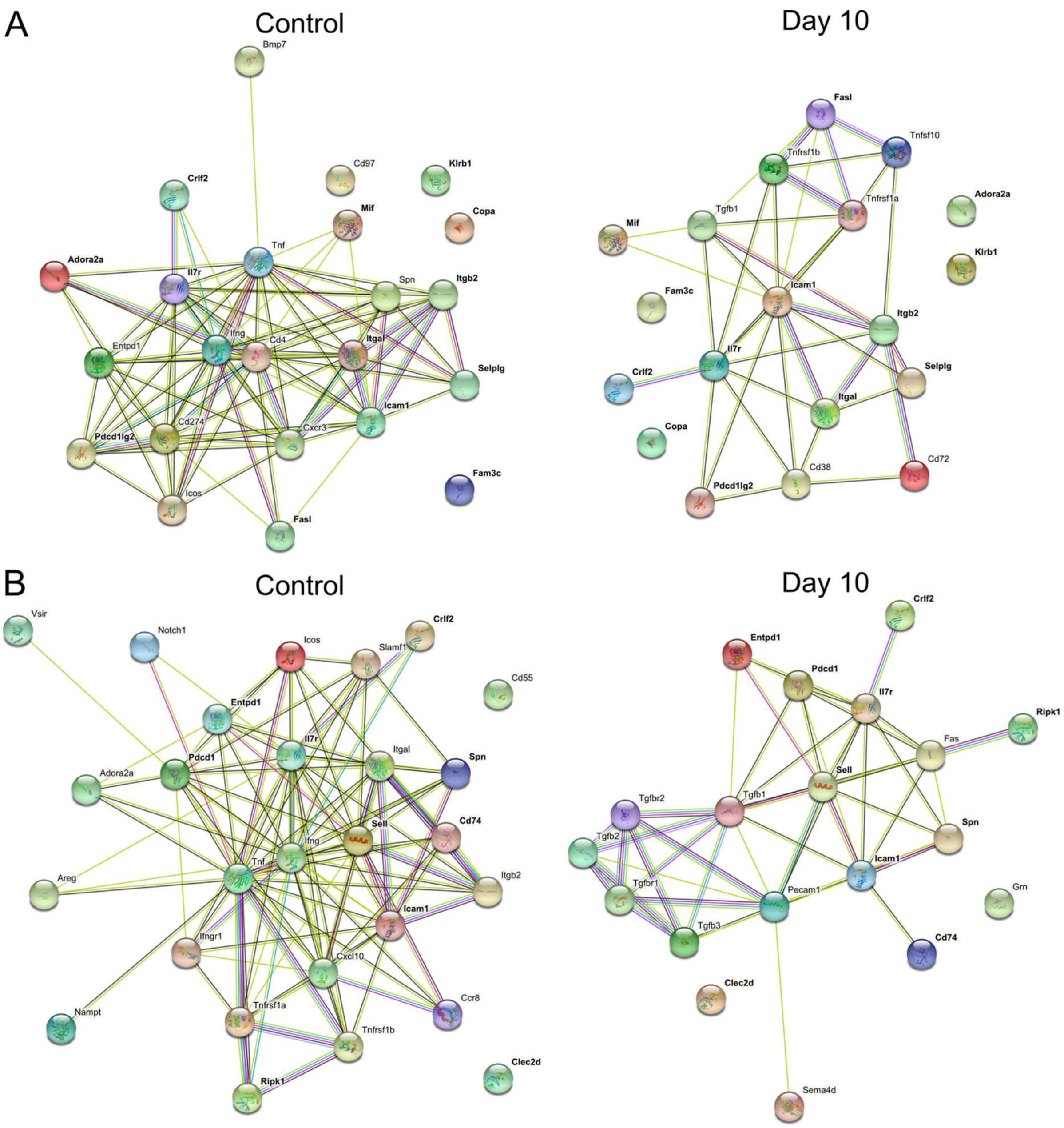

Figure S8. Proteins that form interaction pairs between NKT cells and Tregs display high connectivity within respective cell types. Protein-protein interaction networks are shown for NKT cells (A) and Tregs (B) for genes that form statistically significant predicted NKT-Tregs interaction pairs in control and D10 samples (Figure 4). Proteins that are found both in control and D10 interaction pairs are highlighted in bold.

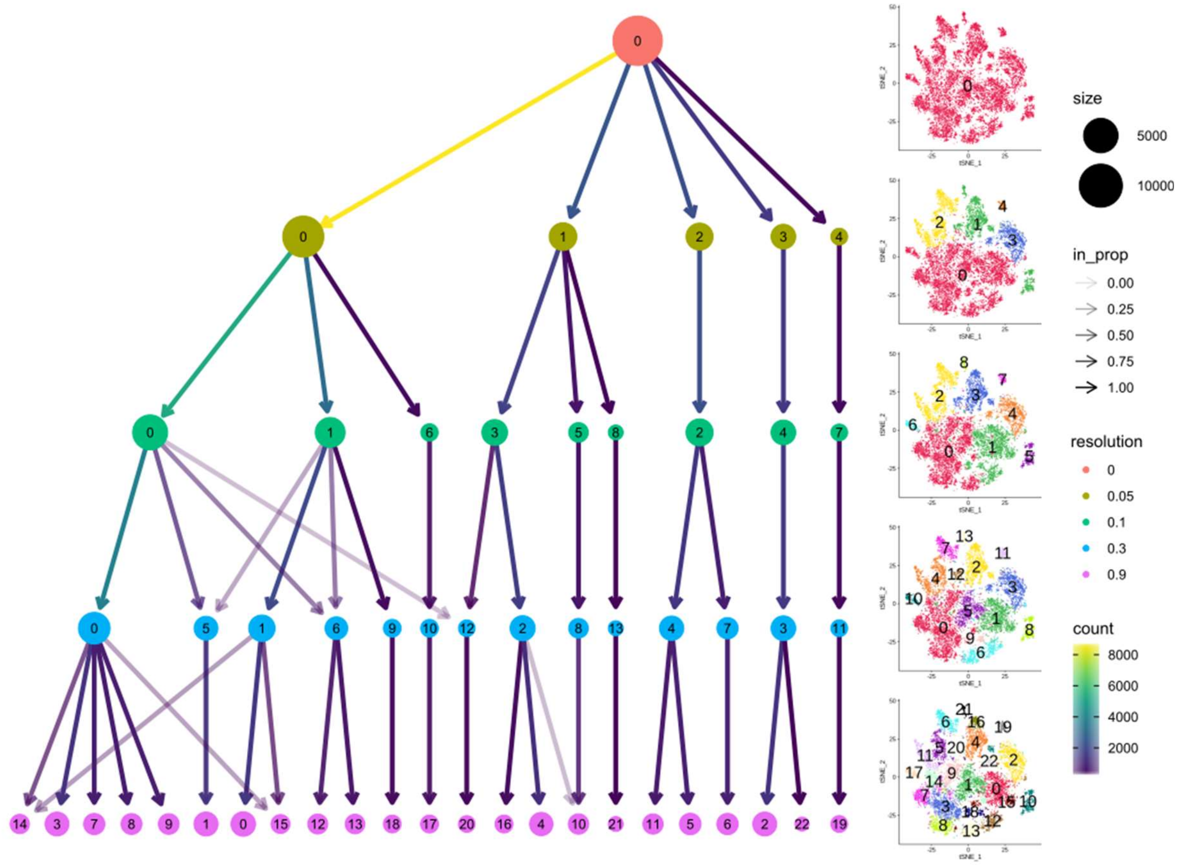

Figure S9. Increasing the clustering resolution allows to highlight a cluster of potential interest. Separation of data on increasing clustering resolution levels is shown schematically (left) as well as on tSNE representations of the dataset (right). Cluster 15 from resolution level 0.9 was reintroduced to the dataset. The number of cells (count) split into each cluster is represented by the color of the arrows; arrow transparency shows the incoming node proportion (in_prop).

Supplemental Table 1. Composition of different T cell subtypes in the dataset.

	Control		Day 10		Day 21		
Cell type (generic)	C1D21	C1D48	AE1D10	AE2D10	AE5D21	AE6D21	AE7D21
NKT cells	35	158	347	296	125	223	120
T cells (CD4+)	460	1421	226	425	273	969	303
T cells (CD8+)	222	1061	87	235	131	648	247
T cells (Regulatory)	11	33	22	38	33	51	27
Tgd cells	28	55	17	38	14	42	20

	Control		Day 10		Day 21		
Cell type (advanced)	C1D21	C1D48	AE1D10	AE2D10	AE5D21	AE6D21	AE7D21
NKT (CD4-)	8	14	55	55	20	22	21
NKT (CD4+)	10	15	266	203	86	173	65
NKT (CD44+ NK1.1-)	2	3	3	12	4	4	0
NKT (CD44+ NK1.1+)	15	126	23	26	15	24	34
T cells (CD4+ Memory)	12	62	20	29	8	31	11
T cells (CD4+ Naive)	3	20	3	2	1	9	2
T cells (CD4+)	445	1339	203	394	264	929	290
T cells (CD8+ Activated)	40	118	28	42	20	51	38
T cells (CD8+ Memory)	92	394	39	137	42	190	114
T cells (CD8+ Naive)	20	165	15	31	17	131	24
T cells (CD8+)	70	384	5	25	52	276	71
T cells (Regulatory)	11	33	22	38	33	51	27
Tgd (VG2+)	28	55	17	38	14	42	20

Day 48		
AE1D48	AE2D48	AE3D48
58	196	739
384	1147	1449
188	758	1228
29	42	86
12	50	41

Day 48		
AE1D48	AE2D48	AE3D48
6	26	125
36	92	528
1	6	5
15	72	81
14	16	31
2	14	22
368	1117	1396
43	131	114
48	225	286
17	130	283
80	272	545
29	42	86
12	50	41

Supplemental Table 2. List of significant markers for each of the clusters.

gene	p_val	avg_log2F	pct.1	pct.2	p_val_adj	cluster
Igfbp4	4,7E-286	1,35691	0,525	0,242	1,5E-281	1
Lef1	3,8E-228	0,796172	0,739	0,527	1,2E-223	1
Dusp10	1E-214	0,988733	0,598	0,362	3,1E-210	1
Rps8	2,5E-214	0,334264	1	0,995	7,8E-210	1
Rps29	5,8E-212	0,319437	0,999	0,996	1,8E-207	1
Ccr7	4,5E-199	0,808151	0,729	0,476	1,4E-194	1
Rps20	9,1E-192	0,3526	0,999	0,994	2,8E-187	1
Gimap6	5,3E-175	0,65266	0,894	0,822	1,6E-170	1
Rps3a1	7,4E-174	0,28494	0,999	0,995	2,3E-169	1
Rpl35a	3,9E-172	0,282321	0,997	0,992	1,2E-167	1
Rps24	9,5E-162	0,251141	1	0,997	2,9E-157	1
Cytip	9,4E-149	0,635251	0,757	0,633	2,9E-144	1
Tcf7	8,8E-142	0,647028	0,73	0,582	2,7E-137	1
Rps4x	4,4E-141	0,25956	0,999	0,993	1,4E-136	1
Rpl8	9,8E-141	0,268528	0,998	0,991	3,1E-136	1
Satb1	2,6E-127	0,639354	0,682	0,54	8E-123	1
Cd4	4,9E-117	0,520121	0,365	0,183	1,5E-112	1
Limd2	1,4E-115	0,425318	0,897	0,841	4,2E-111	1
Rplp0	2,1E-113	0,254756	0,999	0,992	6,4E-109	1
Rps19	1,1E-110	0,280954	0,993	0,985	3,6E-106	1
Klf2	1,2E-108	0,548636	0,846	0,716	3,8E-104	1
Rpl35	1,3E-107	0,256906	0,992	0,982	4,1E-103	1
Pnrc1	2,2E-107	0,392967	0,943	0,924	7E-103	1
Npc2	6,93E-94	0,450528	0,776	0,692	2,15E-89	1
Frat2	4,43E-87	0,62249	0,394	0,254	1,38E-82	1
Selenop	5,53E-85	0,636172	0,414	0,271	1,72E-80	1
Trib2	1,26E-84	0,61333	0,25	0,125	3,9E-80	1
S1pr1	2,12E-79	0,582081	0,462	0,326	6,57E-75	1
Actn1	2,71E-79	0,53422	0,44	0,297	8,42E-75	1
Dgka	4,14E-76	0,494322	0,606	0,503	1,29E-71	1
Gm20400	5,31E-75	0,855212	0,368	0,237	1,65E-70	1
Rflnb	3,5E-73	0,591226	0,434	0,305	1,09E-68	1
Ass1	3,79E-73	0,499168	0,406	0,272	1,18E-68	1
Il6ra	1,16E-70	0,503246	0,272	0,153	3,61E-66	1
Foxp1	5,96E-68	0,50553	0,588	0,494	1,85E-63	1
Rgs10	2,13E-66	0,541723	0,531	0,423	6,61E-62	1
Bach2	2,23E-65	0,502148	0,311	0,193	6,91E-61	1
Vps37b	5,19E-61	0,437515	0,781	0,732	1,61E-56	1
Arhgef1	7,52E-61	0,397462	0,754	0,723	2,33E-56	1
Btg1	1,23E-56	0,323811	0,902	0,886	3,8E-52	1
Sell	1,23E-54	0,418584	0,491	0,37	3,83E-50	1
Smc4	3,19E-50	0,565929	0,475	0,384	9,9E-46	1
Znrf1	6,11E-50	0,459299	0,352	0,251	1,9E-45	1
Crlf3	1,43E-49	0,513048	0,43	0,341	4,45E-45	1
Tspan32	4,99E-47	0,427127	0,311	0,209	1,55E-42	1
Atp1b3	9,21E-46	0,362382	0,623	0,555	2,86E-41	1
Cd247	3,57E-42	0,369754	0,569	0,502	1,11E-37	1
Gm8369	1,59E-40	0,434101	0,475	0,389	4,95E-36	1
Klf13	7,02E-38	0,377032	0,582	0,529	2,18E-33	1
Foxo1	3,05E-37	0,406052	0,415	0,333	9,48E-33	1
Ets1	6,99E-35	0,290758	0,725	0,693	2,17E-30	1
Rasgrp2	9,01E-35	0,380715	0,476	0,401	2,8E-30	1
Pdk1	1,41E-32	0,392931	0,263	0,189	4,38E-28	1

Smad7	8,59E-31	0,401081	0,391	0,318	2,67E-26	1
Tsc22d3	4,66E-30	0,26986	0,669	0,607	1,45E-25	1
Gpr183	1,88E-29	0,356783	0,354	0,276	5,83E-25	1
Rbm38	4,67E-29	0,404841	0,459	0,401	1,45E-24	1
Stk17b	2,96E-26	0,293482	0,698	0,689	9,18E-22	1
Nsg2	1,11E-25	0,327711	0,34	0,27	3,45E-21	1
Peli1	7,16E-25	0,317928	0,554	0,508	2,22E-20	1
Atp6v1d	4,74E-24	0,382115	0,279	0,216	1,47E-19	1
Arhgap31	6,06E-24	0,350512	0,321	0,254	1,88E-19	1
Pik3ip1	1,83E-23	0,358694	0,261	0,199	5,67E-19	1
Chd3	4,72E-23	0,32018	0,544	0,509	1,46E-18	1
Emb	1,54E-22	0,260174	0,577	0,519	4,78E-18	1
Pdlim1	1,28E-20	0,330108	0,267	0,21	3,98E-16	1
Rgcc	1,06E-18	0,254626	0,383	0,325	3,3E-14	1
Oser1	1,95E-18	0,326106	0,351	0,301	6,06E-14	1
Bcl11b	2,62E-18	0,303481	0,43	0,388	8,13E-14	1
Smc6	5,26E-18	0,342741	0,309	0,259	1,63E-13	1
Cited2	8,02E-18	0,34416	0,456	0,412	2,49E-13	1
Retreg1	1,05E-16	0,275395	0,259	0,206	3,28E-12	1
Cd5	7,49E-16	0,275193	0,345	0,295	2,33E-11	1
Cirbp	7,52E-16	0,268179	0,463	0,426	2,34E-11	1
Add3	1,55E-15	0,262514	0,329	0,281	4,82E-11	1
Txnip	1,56E-15	0,307538	0,578	0,561	4,85E-11	1
Ube2d2a	4,14E-14	0,265673	0,524	0,509	1,29E-09	1
Ube2b	4,36E-14	0,279304	0,462	0,442	1,35E-09	1
Hmgn1	1,12E-13	0,295548	0,381	0,345	3,47E-09	1
Tob1	1,19E-13	0,305898	0,309	0,266	3,7E-09	1
Tgfbr2	1,73E-13	0,262992	0,394	0,357	5,36E-09	1
Cdkn1b	2,85E-13	0,276694	0,296	0,253	8,86E-09	1
Ctss	4,42E-13	0,276689	0,28	0,237	1,37E-08	1
Epc1	2,89E-10	0,271239	0,373	0,347	8,96E-06	1
Cd8b1	0	1,54382	0,898	0,261	0	2
Cd8a	0	1,162577	0,665	0,194	0	2
Rplp1	1,4E-263	0,511004	1	0,992	4,3E-259	2
Rps15a	7E-233	0,413319	1	0,992	2,2E-228	2
Rpl32	1,3E-229	0,433206	1	0,989	4,1E-225	2
Rps28	3,6E-216	0,4337	1	0,989	1,1E-211	2
Rpl13	1,5E-208	0,373302	1	0,997	4,8E-204	2
Rpl41	4,6E-208	0,350252	1	0,996	1,4E-203	2
Rpl35a	2,8E-206	0,402534	1	0,992	8,8E-202	2
Rps7	1,5E-205	0,402329	1	0,993	4,6E-201	2
Rpl35	3,5E-204	0,464431	1	0,983	1,1E-199	2
Rplp0	6,5E-204	0,43165	1	0,993	2E-199	2
Fam241a	2,4E-200	0,844967	0,389	0,115	7,4E-196	2
Rps24	7E-197	0,349354	1	0,997	2,2E-192	2
Rps20	1,2E-191	0,440419	1	0,994	3,6E-187	2
Rpl39	1,7E-187	0,3826	1	0,992	5,3E-183	2
Rps10	4,2E-186	0,361412	0,999	0,994	1,3E-181	2
Rpl23	4,6E-186	0,370702	1	0,995	1,4E-181	2
Rpl28	5,9E-184	0,448543	0,999	0,983	1,8E-179	2
Rps3a1	2,6E-183	0,357086	1	0,995	8,2E-179	2
Rpsa	4,7E-182	0,373856	1	0,996	1,5E-177	2
Rps4x	1,9E-176	0,371439	1	0,994	5,8E-172	2
Rps18	2,7E-168	0,437988	0,999	0,972	8,3E-164	2
Rps8	4,6E-167	0,370401	1	0,996	1,4E-162	2

Rpl15	6,4E-165	0,418176	1	0,975	2E-160	2
Rpl36a	2,2E-163	0,464119	0,998	0,962	7E-159	2
Rps3	4,8E-161	0,340106	1	0,991	1,5E-156	2
Rps26	1,4E-160	0,391464	1	0,989	4,2E-156	2
Rps5	2,2E-158	0,348767	0,999	0,992	6,8E-154	2
Rpl10a	1,2E-154	0,397232	1	0,975	3,9E-150	2
Eef1a1	2,2E-147	0,294046	1	0,998	7E-143	2
Rpl37a	1,3E-146	0,339148	0,999	0,992	4E-142	2
Rpl27a	2E-146	0,329607	0,999	0,989	6,2E-142	2
Rpl34	2,8E-144	0,305938	1	0,991	8,7E-140	2
Rps2	1,2E-143	0,358875	1	0,992	3,6E-139	2
Rpl37	7,6E-142	0,305653	1	0,995	2,4E-137	2
Rps27a	1E-135	0,311622	1	0,994	3,2E-131	2
Rpl30	2,1E-133	0,302545	1	0,995	6,5E-129	2
Eef1b2	2,9E-133	0,425934	0,996	0,949	9E-129	2
Rps19	3,9E-131	0,386362	1	0,985	1,2E-126	2
Rps29	1,2E-130	0,322288	1	0,996	3,6E-126	2
Rpl21	6,8E-130	0,310587	1	0,995	2,1E-125	2
Rps16	9,5E-129	0,288294	1	0,995	3E-124	2
Rpl11	2,3E-128	0,299036	0,999	0,992	7,1E-124	2
Rps21	9,5E-127	0,305321	0,999	0,991	2,9E-122	2
Rpl3	3,8E-124	0,359444	0,998	0,975	1,2E-119	2
Rpl8	3E-123	0,3165	1	0,992	9,2E-119	2
Rpl10	4,4E-121	0,361123	0,997	0,97	1,4E-116	2
Rpl26	3,9E-120	0,348595	0,999	0,977	1,2E-115	2
Rpl36	2,1E-117	0,281266	1	0,991	6,6E-113	2
Rps6	1,2E-116	0,390144	0,996	0,946	3,7E-112	2
Rpl9	1,3E-114	0,262285	1	0,995	3,9E-110	2
Rps13	8,1E-113	0,269709	1	0,993	2,5E-108	2
Rps23	9E-113	0,329387	0,999	0,98	2,8E-108	2
Tmem108	1,8E-112	0,544452	0,253	0,078	5,6E-108	2
Rpl18	1,9E-110	0,261224	1	0,994	6E-106	2
Rps9	2,1E-109	0,309726	0,999	0,984	6,5E-105	2
Dapl1	5,4E-109	0,802055	0,438	0,2	1,7E-104	2
Rpl6	2,9E-108	0,277433	1	0,993	8,9E-104	2
Rpl17	3,2E-106	0,317596	1	0,984	1E-101	2
Ccr7	1,2E-104	0,621049	0,796	0,507	3,9E-100	2
Rplp2	1,6E-103	0,286153	0,999	0,99	5,1E-99	2
Rpl12	2,8E-102	0,355022	0,996	0,968	8,59E-98	2
Rps14	2,9E-102	0,298103	0,999	0,985	9,05E-98	2
Rps27	2,1E-100	0,2902	1	0,991	6,58E-96	2
Rpl7	7,62E-98	0,330941	0,996	0,964	2,37E-93	2
Rps12	3,33E-90	0,352633	0,997	0,958	1,03E-85	2
Rack1	1,87E-82	0,33473	0,99	0,947	5,79E-78	2
Rpl14	1,86E-75	0,297563	0,999	0,97	5,77E-71	2
Rpl5	2,74E-73	0,311121	0,992	0,949	8,52E-69	2
Rpl4	3,12E-71	0,328227	0,985	0,925	9,69E-67	2
Rpl13a	1,03E-69	0,292974	0,998	0,969	3,2E-65	2
Rpl22l1	2,22E-66	0,37585	0,962	0,871	6,88E-62	2
Rpl7a	3,5E-62	0,282413	0,992	0,95	1,09E-57	2
Rps25	1,4E-60	0,328954	0,958	0,873	4,36E-56	2
Rpl23a	5,07E-58	0,317808	0,975	0,902	1,58E-53	2
Dnajc15	8,37E-56	0,417713	0,581	0,376	2,6E-51	2
Nme2	1,06E-52	0,344174	0,897	0,757	3,3E-48	2
Slc25a4	3,39E-51	0,416529	0,427	0,249	1,05E-46	2

Rps15	2,57E-50	0,275297	0,985	0,929	7,99E-46	2
Npc2	7,62E-50	0,345158	0,861	0,694	2,37E-45	2
Rps17	4,56E-48	0,317312	0,932	0,826	1,41E-43	2
Npm1	1,17E-47	0,293915	0,962	0,875	3,64E-43	2
Sell	9,2E-47	0,36826	0,583	0,377	2,86E-42	2
Cox7a2l	3,83E-44	0,347166	0,822	0,662	1,19E-39	2
Tubb5	8,13E-43	0,353114	0,776	0,621	2,53E-38	2
Hspe1	2,64E-41	0,349305	0,751	0,589	8,2E-37	2
Acp5	1,85E-40	0,402929	0,505	0,339	5,75E-36	2
Plac8	1,06E-31	0,260614	0,255	0,141	3,29E-27	2
Klk8	3,43E-29	0,327655	0,472	0,331	1,06E-24	2
Hmgn1	4,92E-26	0,256547	0,477	0,338	1,53E-21	2
Eef1g	2,68E-24	0,268233	0,807	0,684	8,33E-20	2
Epsti1	3,42E-24	0,268308	0,543	0,405	1,06E-19	2
Gm10076	1,36E-23	0,289062	0,444	0,318	4,22E-19	2
Fam189b	4,77E-21	0,282106	0,444	0,325	1,48E-16	2
Il2rb	0	2,104079	0,924	0,316	0	3
Cxcr6	0	2,088897	0,704	0,103	0	3
Xcl1	0	1,968384	0,619	0,06	0	3
Klrb1c	0	1,841089	0,547	0,023	0	3
Id2	0	1,771427	0,863	0,337	0	3
Klrk1	0	1,634873	0,51	0,061	0	3
Ckb	0	1,625469	0,5	0,056	0	3
Ifng	0	1,526843	0,532	0,091	0	3
Ly6e	0	1,400227	0,981	0,78	0	3
Klrc1	0	1,373887	0,324	0,033	0	3
Nkg7	0	1,357685	0,936	0,416	0	3
Gzmb	0	1,320836	0,365	0,054	0	3
Hopx	0	1,304677	0,691	0,216	0	3
Ly6a	0	1,301765	0,702	0,21	0	3
AW11201(	0	1,300867	0,948	0,604	0	3
Rcbtb2	0	1,292551	0,447	0,092	0	3
Sh3bgrl3	0	1,26884	0,984	0,851	0	3
Gimap4	0	1,25833	0,903	0,579	0	3
Ctsw	0	1,226859	0,815	0,378	0	3
Mmp9	0	1,204783	0,316	0,022	0	3
Ccr2	0	1,133943	0,402	0,056	0	3
Bhlhe40	0	1,098993	0,562	0,155	0	3
Il4	0	1,026515	0,304	0,023	0	3
Fgl2	0	1,014302	0,305	0,033	0	3
Klrc2	0	0,995845	0,254	0,017	0	3
Cd160	0	0,987234	0,323	0,052	0	3
Socs2	0	0,967961	0,296	0,037	0	3
Cd52	0	0,914642	0,992	0,917	0	3
Il12rb2	0	0,865905	0,277	0,03	0	3
Zfp683	0	0,856662	0,252	0,019	0	3
Asb2	0	0,826152	0,265	0,032	0	3
Tmsb4x	0	0,700907	0,999	0,993	0	3
Cxcr3	1,8E-306	1,047554	0,422	0,092	5,7E-302	3
S100a6	4,8E-297	0,993199	0,649	0,203	1,5E-292	3
Rac2	1,5E-294	0,809628	0,985	0,905	4,7E-290	3
Prrt1	7,3E-291	0,910701	0,312	0,052	2,3E-286	3
Arsb	3,6E-280	0,890018	0,315	0,056	1,1E-275	3
P2rx7	4,2E-279	0,855872	0,263	0,037	1,3E-274	3
Cd226	1,5E-277	1,037703	0,433	0,109	4,6E-273	3

Coro2a	4,4E-263	0,840562	0,289	0,048	1,4E-258	3
Gimap3	1,6E-259	1,062096	0,903	0,649	5,1E-255	3
Slamf7	1,4E-258	0,802469	0,272	0,043	4,4E-254	3
Gm19585	1,3E-257	1,076476	0,459	0,131	4,1E-253	3
Cyba	8,4E-255	0,975661	0,905	0,627	2,6E-250	3
Trbv13-2	1E-252	1,672459	0,49	0,159	3,3E-248	3
Lyst	3E-248	1,110203	0,444	0,128	9,2E-244	3
Tbx21	2,2E-247	0,84804	0,318	0,063	7E-243	3
Pfn1	6,1E-228	0,612801	0,992	0,966	1,9E-223	3
Itgal	2,2E-227	1,027455	0,673	0,303	6,9E-223	3
Sptssa	2,8E-227	1,063815	0,61	0,255	8,8E-223	3
Myl6	5,9E-227	0,758236	0,973	0,868	1,8E-222	3
Dok2	7,1E-217	0,978017	0,5	0,175	2,2E-212	3
Bcl2a1d	1,5E-207	0,780968	0,305	0,067	4,5E-203	3
Bcl2a1b	1,3E-206	0,974409	0,475	0,157	4,2E-202	3
Nedd4	1,6E-206	0,725864	0,255	0,048	4,9E-202	3
Cd44	2,7E-206	0,859129	0,425	0,128	8,2E-202	3
Efhd2	6,3E-206	0,939872	0,545	0,212	1,9E-201	3
Lpcat4	2,3E-201	0,887929	0,444	0,145	7,3E-197	3
Fth1	7,6E-200	0,608126	0,99	0,967	2,4E-195	3
AU020206	3,7E-198	0,822525	0,401	0,118	1,1E-193	3
Ctla2a	2,5E-197	0,819237	0,434	0,134	7,8E-193	3
Ccnd2	1,7E-196	0,987785	0,736	0,414	5,2E-192	3
Itgb2	1,4E-193	1,00335	0,709	0,386	4,4E-189	3
Dusp1	2,9E-192	1,357067	0,784	0,527	9E-188	3
Ifitm10	5,4E-189	0,8376	0,362	0,103	1,7E-184	3
Rgs3	4,8E-187	0,818718	0,358	0,102	1,5E-182	3
Thy1	2E-186	0,788373	0,923	0,733	6,2E-182	3
Cd82	8,9E-185	0,931608	0,62	0,282	2,7E-180	3
Ctsd	6E-182	0,90448	0,72	0,403	1,9E-177	3
Ptpn7	9,6E-182	0,826239	0,419	0,138	3E-177	3
Samsn1	1,9E-180	0,815237	0,406	0,128	5,9E-176	3
H2-K1	6,2E-177	0,50123	0,998	0,98	1,9E-172	3
Dennd4a	1,6E-175	0,999332	0,528	0,222	5E-171	3
Serinc3	2,4E-174	0,908794	0,649	0,33	7,4E-170	3
Ptprcap	1,3E-171	0,819244	0,855	0,611	3,9E-167	3
Ppp1r12a	7,1E-168	0,880078	0,691	0,376	2,2E-163	3
Trip4	1,7E-167	0,666181	0,256	0,059	5,3E-163	3
Ccnd3	1,5E-163	0,886841	0,713	0,413	4,7E-159	3
H2-Q6	1E-157	0,787755	0,816	0,539	3,2E-153	3
Lcp1	1,4E-157	0,817799	0,833	0,559	4,2E-153	3
Cox8a	1,2E-156	0,655311	0,941	0,788	3,8E-152	3
Gimap7	1,2E-154	0,847063	0,52	0,221	3,7E-150	3
Padi2	8,6E-154	0,687692	0,262	0,066	2,7E-149	3
Rgs1	3,1E-145	0,867358	0,584	0,278	9,7E-141	3
B2m	7,7E-145	0,445627	0,992	0,978	2,4E-140	3
S100a10	2,2E-144	0,529755	0,868	0,601	6,8E-140	3
Tnfrsf18	4,9E-143	0,748346	0,465	0,19	1,5E-138	3
Adgre5	9,4E-141	0,825319	0,716	0,428	2,9E-136	3
Ly6c2	3,1E-140	1,35806	0,484	0,214	9,6E-136	3
H2-D1	5,4E-138	0,398192	0,996	0,982	1,7E-133	3
Hcst	1,2E-136	0,651812	0,922	0,776	3,8E-132	3
Sema4a	6,5E-135	0,723689	0,379	0,139	2E-130	3
Unc119	1,7E-134	0,627705	0,252	0,067	5,4E-130	3
Sytl3	1,5E-133	0,776726	0,411	0,159	4,5E-129	3

Ucp2	5E-129	0,694807	0,846	0,64	1,6E-124	3
Cd3g	2,3E-128	0,564153	0,941	0,836	7E-124	3
Sept1	2E-126	0,638214	0,882	0,716	6,1E-122	3
S100a11	1,2E-125	0,55578	0,704	0,401	3,8E-121	3
H2-Q7	1,2E-125	0,576668	0,931	0,807	3,9E-121	3
Actb	1,8E-125	0,440546	0,997	0,988	5,6E-121	3
Prr13	9E-124	0,643157	0,569	0,275	2,8E-119	3
Psmb8	1,5E-123	0,64125	0,847	0,654	4,5E-119	3
Aph1a	1,9E-118	0,72055	0,404	0,169	5,9E-114	3
Klrd1	1E-116	0,857738	0,472	0,226	3,2E-112	3
Adam19	4,7E-115	0,63647	0,256	0,079	1,4E-110	3
Art2b	3,7E-114	0,668963	0,258	0,081	1,2E-109	3
Ahnak	9,8E-113	0,625167	0,569	0,281	3E-108	3
Rora	1,6E-111	0,635832	0,266	0,083	4,9E-107	3
St3gal6	1,6E-106	0,658029	0,339	0,133	4,9E-102	3
Gabarapl2	5,8E-104	0,635121	0,688	0,443	1,8E-99	3
Lamtor4	6,1E-104	0,659412	0,404	0,178	1,9E-99	3
Dusp2	6,8E-104	0,821607	0,622	0,371	2,1E-99	3
Ikzf3	6,3E-102	0,654996	0,38	0,161	1,94E-97	3
Ndufa4	1,4E-101	0,597637	0,792	0,567	4,27E-97	3
Ppp1r18	2,6E-99	0,626207	0,79	0,564	8E-95	3
Ndfip1	9,54E-98	0,618999	0,735	0,508	2,96E-93	3
Cish	2,53E-95	0,517004	0,273	0,094	7,85E-91	3
Bcap31	6,53E-94	0,675385	0,47	0,249	2,03E-89	3
Pglyrp1	1,15E-93	0,600954	0,346	0,144	3,57E-89	3
Serf2	1,65E-93	0,422322	0,97	0,899	5,11E-89	3
Sh2d2a	7,56E-93	0,639835	0,6	0,358	2,35E-88	3
Nucb1	4,56E-91	0,612674	0,309	0,125	1,42E-86	3
Zfp36l2	6,59E-91	0,650844	0,841	0,663	2,05E-86	3
Zyx	1,06E-90	0,652172	0,569	0,324	3,31E-86	3
Ubl5	1,62E-90	0,561236	0,779	0,573	5,04E-86	3
Pla2g16	1,84E-90	0,649637	0,437	0,218	5,72E-86	3
Sept9	2,94E-90	0,6378	0,582	0,341	9,12E-86	3
Lgals1	7,53E-88	0,435954	0,619	0,357	2,34E-83	3
Rbl2	1,25E-87	0,619849	0,319	0,133	3,89E-83	3
Cyth4	9,08E-87	0,580574	0,416	0,2	2,82E-82	3
Cd40lg	3,08E-86	0,471387	0,352	0,146	9,56E-82	3
Pglyrp2	1,51E-85	0,565095	0,284	0,111	4,7E-81	3
Gng2	2,09E-85	0,5305	0,277	0,107	6,5E-81	3
Mxd4	1,29E-84	0,601765	0,368	0,167	4,02E-80	3
Ctsb	1,69E-84	0,595609	0,438	0,222	5,25E-80	3
Capzb	1,79E-84	0,562841	0,764	0,57	5,56E-80	3
Wipf1	2,86E-84	0,61627	0,481	0,26	8,89E-80	3
Malat1	1,62E-83	0,484186	0,992	0,984	5,02E-79	3
Myo1f	2,79E-83	0,51885	0,256	0,094	8,65E-79	3
Dtx1	1,67E-82	0,604443	0,363	0,164	5,2E-78	3
Lax1	2,3E-82	0,540932	0,264	0,102	7,14E-78	3
Bcl2	3E-82	0,627853	0,613	0,38	9,32E-78	3
Sla	5,45E-82	0,565184	0,39	0,185	1,69E-77	3
2010111I0	3,98E-80	0,590343	0,36	0,169	1,24E-75	3
Ppia	4,66E-80	0,308747	0,994	0,986	1,45E-75	3
Zap70	9,13E-79	0,58708	0,531	0,313	2,84E-74	3
Cfl1	4,28E-78	0,376233	0,964	0,919	1,33E-73	3
Plac8	5,99E-78	0,729002	0,306	0,135	1,86E-73	3
Sipa1	2,3E-77	0,591098	0,382	0,189	7,14E-73	3

Actg1	4,07E-76	0,380188	0,973	0,923	1,26E-71	3
Cd37	6,65E-76	0,560766	0,703	0,498	2,07E-71	3
Apobec3	3,52E-75	0,569418	0,425	0,221	1,09E-70	3
Nbeal2	5,06E-75	0,516546	0,251	0,098	1,57E-70	3
Cd3e	8E-75	0,396292	0,954	0,89	2,49E-70	3
Lsp1	1,08E-74	0,427869	0,798	0,604	3,34E-70	3
Aldoa	9,62E-74	0,564175	0,742	0,558	2,99E-69	3
Pdia3	1,47E-73	0,584739	0,655	0,445	4,56E-69	3
Vamp8	8,16E-73	0,51408	0,51	0,291	2,53E-68	3
Lasp1	9,15E-73	0,574985	0,374	0,184	2,84E-68	3
Pik3r1	1,12E-72	0,5766	0,374	0,186	3,48E-68	3
Mbnl1	1,17E-72	0,499718	0,84	0,688	3,64E-68	3
Hsp90b1	4,09E-72	0,606558	0,599	0,385	1,27E-67	3
B4galnt1	1,24E-71	0,551372	0,762	0,578	3,84E-67	3
Nedd9	2,92E-70	0,552236	0,346	0,166	9,05E-66	3
Clic1	5,74E-69	0,500869	0,795	0,623	1,78E-64	3
Junb	2,59E-68	0,481349	0,963	0,959	8,04E-64	3
Gimap5	8,07E-67	0,518385	0,641	0,429	2,51E-62	3
Inpp4b	2,38E-65	0,571779	0,513	0,314	7,39E-61	3
Ptpn18	2,69E-65	0,392357	0,931	0,851	8,34E-61	3
Ppp1r9b	4,29E-65	0,539713	0,307	0,143	1,33E-60	3
Actr3	2,6E-64	0,442735	0,831	0,673	8,06E-60	3
Csnk2b	7,46E-64	0,539633	0,552	0,355	2,32E-59	3
Sec11c	1,35E-63	0,530441	0,542	0,339	4,2E-59	3
Skap1	3,68E-63	0,48979	0,784	0,62	1,14E-58	3
D16Ertd47	1,17E-62	0,533648	0,331	0,164	3,62E-58	3
Ndufa13	1,74E-62	0,47028	0,767	0,584	5,39E-58	3
Dnajc3	2,08E-62	0,5215	0,53	0,331	6,45E-58	3
Vars	1,39E-61	0,492602	0,334	0,167	4,31E-57	3
Isg15	7,16E-61	0,387803	0,259	0,111	2,22E-56	3
Ppp1r11	3,96E-60	0,499144	0,343	0,175	1,23E-55	3
Icos	3,94E-59	0,476122	0,359	0,185	1,22E-54	3
Pde3b	6,17E-59	0,525338	0,373	0,2	1,92E-54	3
Ptpn22	6,58E-59	0,510243	0,486	0,295	2,04E-54	3
Prex1	2,54E-58	0,490589	0,41	0,23	7,89E-54	3
Ly9	2,84E-58	0,536953	0,333	0,172	8,83E-54	3
Ifngr1	6,42E-58	0,580554	0,642	0,455	1,99E-53	3
Calm1	9,22E-58	0,407451	0,902	0,8	2,86E-53	3
Clec2d	1,24E-57	0,554028	0,436	0,251	3,85E-53	3
Icam1	1,68E-57	0,679192	0,265	0,123	5,21E-53	3
Trac	2,45E-57	0,494865	0,748	0,585	7,6E-53	3
Calm3	6,73E-57	0,513349	0,472	0,289	2,09E-52	3
Laptm5	1,01E-56	0,340491	0,901	0,79	3,13E-52	3
Shisa5	1,19E-56	0,327396	0,959	0,905	3,7E-52	3
Selplg	6,19E-56	0,437468	0,812	0,666	1,92E-51	3
Fam204a	5,96E-55	0,41357	0,262	0,12	1,85E-50	3
Aplp2	7,8E-55	0,456636	0,252	0,114	2,42E-50	3
Hcls1	1,14E-54	0,472199	0,454	0,275	3,55E-50	3
Aes	1,23E-54	0,485158	0,743	0,588	3,81E-50	3
Prdx5	1,41E-54	0,45645	0,465	0,281	4,37E-50	3
Adcy7	2,5E-54	0,4978	0,366	0,202	7,76E-50	3
Gpr65	3,91E-54	0,456059	0,256	0,118	1,21E-49	3
Gimap8	5,81E-54	0,51313	0,39	0,22	1,8E-49	3
Tmem64	1,37E-53	0,474338	0,328	0,167	4,26E-49	3
Pgam1	1,98E-53	0,480514	0,327	0,173	6,15E-49	3

Ost4	3,62E-53	0,45028	0,702	0,528	1,12E-48	3
Gata3	3,73E-53	0,41748	0,33	0,167	1,16E-48	3
Rinl	5,91E-53	0,506815	0,467	0,291	1,83E-48	3
Fnbp1	7,3E-53	0,462714	0,422	0,246	2,27E-48	3
Zmiz1	1,57E-52	0,453078	0,283	0,138	4,89E-48	3
Atp6v0e	5,6E-52	0,458483	0,496	0,312	1,74E-47	3
Runx3	6,53E-52	0,476236	0,36	0,198	2,03E-47	3
Diaph1	6,8E-52	0,465177	0,395	0,227	2,11E-47	3
Pde7a	8,9E-52	0,497767	0,34	0,184	2,76E-47	3
Prkar1a	1,67E-50	0,463293	0,628	0,456	5,2E-46	3
Cox6c	2,19E-50	0,399083	0,822	0,716	6,79E-46	3
Ptk2b	2,31E-50	0,50594	0,357	0,2	7,18E-46	3
Nr3c1	2,44E-50	0,438975	0,313	0,163	7,58E-46	3
Arpc4	4,39E-50	0,453711	0,645	0,47	1,36E-45	3
Tram1	6,03E-50	0,474401	0,423	0,256	1,87E-45	3
Arpc1b	7,94E-50	0,354049	0,844	0,71	2,47E-45	3
Itm2c	8,92E-50	0,410004	0,366	0,202	2,77E-45	3
Cebpb	7,76E-49	0,481968	0,446	0,269	2,41E-44	3
Atp5e	8,71E-49	0,321067	0,938	0,879	2,7E-44	3
Grina	1,49E-48	0,432834	0,277	0,138	4,64E-44	3
Txk	2,1E-48	0,487364	0,544	0,365	6,52E-44	3
Tln1	3,09E-48	0,47501	0,58	0,414	9,58E-44	3
Hmgb2	6,69E-48	0,369265	0,68	0,496	2,08E-43	3
Rrbp1	2,18E-47	0,43441	0,294	0,152	6,78E-43	3
Btg2	6,99E-47	0,442718	0,61	0,436	2,17E-42	3
Gm26740	8,75E-47	0,425675	0,349	0,195	2,72E-42	3
Uqcc2	1,01E-46	0,442883	0,364	0,208	3,13E-42	3
Krtcap2	5,47E-46	0,458511	0,645	0,48	1,7E-41	3
Pdcd4	1,33E-45	0,447527	0,734	0,59	4,14E-41	3
Sem1	2,45E-45	0,377466	0,8	0,659	7,59E-41	3
Sumo2	3,39E-45	0,405724	0,744	0,602	1,05E-40	3
Capns1	4,74E-45	0,464232	0,473	0,305	1,47E-40	3
Ms4a4b	4,81E-45	0,359562	0,836	0,708	1,49E-40	3
Cdk4	5,08E-45	0,467181	0,386	0,232	1,58E-40	3
Atp5j2	5,31E-45	0,382077	0,777	0,645	1,65E-40	3
Arhgdia	6,29E-45	0,393143	0,672	0,507	1,95E-40	3
Gbp4	7,72E-45	0,378983	0,297	0,156	2,4E-40	3
Spcs2	1,32E-44	0,43119	0,474	0,308	4,1E-40	3
Psap	1,62E-44	0,451	0,598	0,435	5,04E-40	3
Tespa1	3,73E-44	0,445488	0,333	0,188	1,16E-39	3
Cdc42	5,47E-44	0,358531	0,788	0,652	1,7E-39	3
Tgfb1	1,09E-43	0,432526	0,463	0,294	3,38E-39	3
Atp5d	1,39E-43	0,378889	0,774	0,641	4,32E-39	3
Elob	1,43E-43	0,365731	0,772	0,64	4,43E-39	3
Atp2b1	1,73E-43	0,406322	0,372	0,216	5,36E-39	3
Tmbim6	8,05E-43	0,380588	0,71	0,556	2,5E-38	3
Nfkbid	8,63E-43	0,480258	0,3	0,161	2,68E-38	3
Ndufb7	9,09E-42	0,395518	0,516	0,347	2,82E-37	3
Il2rg	1,31E-41	0,346587	0,856	0,745	4,06E-37	3
Slc3a2	1,52E-41	0,489012	0,539	0,38	4,71E-37	3
Cd48	1,74E-41	0,355625	0,384	0,225	5,42E-37	3
Cbl	2E-41	0,445249	0,371	0,224	6,21E-37	3
Abhd17a	8,28E-41	0,408791	0,315	0,177	2,57E-36	3
Cd3d	9,29E-41	0,310161	0,915	0,859	2,89E-36	3
Tecr	1,41E-40	0,412561	0,512	0,349	4,37E-36	3

Epsti1	2,8E-40	0,364167	0,581	0,401	8,7E-36	3
Tpst2	2,83E-40	0,41373	0,444	0,286	8,79E-36	3
Arhgap9	7,28E-40	0,451541	0,46	0,305	2,26E-35	3
H2-Q4	1,24E-39	0,35008	0,804	0,687	3,85E-35	3
Nptn	1,91E-39	0,382846	0,307	0,172	5,92E-35	3
Gnb2	3,63E-39	0,375651	0,651	0,484	1,13E-34	3
Os9	3,69E-39	0,446966	0,324	0,189	1,14E-34	3
Cmtm7	5,08E-39	0,386982	0,346	0,205	1,58E-34	3
Zfp36	5,66E-39	0,429177	0,611	0,453	1,76E-34	3
Tsc22d4	8E-39	0,389002	0,495	0,335	2,48E-34	3
Ndufv3	1,35E-38	0,404778	0,589	0,432	4,18E-34	3
Rnf166	2,01E-38	0,383089	0,402	0,249	6,23E-34	3
Rnaseh2c	2,11E-38	0,394382	0,371	0,228	6,56E-34	3
Tecpr1	2,2E-38	0,445101	0,506	0,354	6,84E-34	3
Rab1b	3,64E-38	0,405537	0,374	0,232	1,13E-33	3
Socs1	1,48E-37	0,43086	0,381	0,239	4,58E-33	3
Gimap1	1,82E-37	0,384649	0,605	0,45	5,64E-33	3
Tmem243	2,33E-37	0,369053	0,266	0,144	7,24E-33	3
Polr2l	2,99E-37	0,379896	0,434	0,282	9,29E-33	3
Lrrfip1	3,27E-37	0,39407	0,418	0,27	1,01E-32	3
S100a13	4,75E-37	0,352515	0,537	0,37	1,47E-32	3
Cap1	5,38E-37	0,381557	0,509	0,353	1,67E-32	3
Gapdh	6,68E-37	0,294297	0,94	0,902	2,07E-32	3
Tpm4	7,13E-37	0,440452	0,341	0,205	2,22E-32	3
Mrps21	7,61E-37	0,428738	0,446	0,298	2,36E-32	3
Tmem30a	1,13E-36	0,394438	0,327	0,193	3,51E-32	3
Ifi27l2a	3,83E-36	0,322942	0,687	0,536	1,19E-31	3
Rps6ka1	4,41E-36	0,376394	0,309	0,179	1,37E-31	3
Ltb	4,53E-36	0,394226	0,83	0,729	1,41E-31	3
Foxn3	1,02E-35	0,39526	0,46	0,309	3,18E-31	3
Atp5g3	1,34E-35	0,320687	0,83	0,716	4,16E-31	3
Hsbp1	2,06E-35	0,381615	0,348	0,212	6,38E-31	3
Rassf5	3,6E-35	0,369348	0,423	0,278	1,12E-30	3
Uqcrb	5,51E-35	0,369784	0,558	0,402	1,71E-30	3
Cops9	3,25E-34	0,35932	0,51	0,359	1,01E-29	3
Plcg1	3,71E-34	0,368327	0,33	0,198	1,15E-29	3
Cox5a	5,51E-34	0,35448	0,625	0,481	1,71E-29	3
Mob3a	8,27E-34	0,351809	0,277	0,156	2,57E-29	3
Vps28	1,44E-33	0,359713	0,563	0,417	4,48E-29	3
Gabarap	2,05E-33	0,361653	0,638	0,497	6,35E-29	3
Rab8a	4,44E-33	0,350263	0,463	0,313	1,38E-28	3
Gramd1a	5,55E-33	0,353823	0,524	0,38	1,72E-28	3
Crlf2	9,59E-33	0,363412	0,288	0,168	2,98E-28	3
Fermt3	1,19E-32	0,37224	0,387	0,251	3,69E-28	3
Fyn	1,24E-32	0,350956	0,508	0,359	3,85E-28	3
Lgals9	1,48E-32	0,356547	0,43	0,287	4,59E-28	3
Neurl3	3,28E-32	0,350019	0,293	0,172	1,02E-27	3
Stat4	3,83E-32	0,356447	0,313	0,191	1,19E-27	3
Ctsa	4,4E-32	0,362049	0,331	0,203	1,37E-27	3
Atp5j	4,43E-32	0,329708	0,715	0,586	1,38E-27	3
Cd28	5,19E-32	0,378748	0,624	0,477	1,61E-27	3
Ddit3	1,41E-31	0,399921	0,251	0,142	4,39E-27	3
Malt1	1,61E-31	0,366932	0,289	0,171	5E-27	3
Gng10	2,04E-31	0,348438	0,278	0,162	6,35E-27	3
Ywhab	5,98E-31	0,367846	0,479	0,337	1,86E-26	3

Tspo	6,44E-31	0,308599	0,672	0,533	2E-26	3
Cd7	7,17E-31	0,400963	0,325	0,209	2,23E-26	3
Mbp	9,03E-31	0,392444	0,324	0,203	2,8E-26	3
Chchd2	9,89E-31	0,293844	0,831	0,742	3,07E-26	3
Ndufa3	2,63E-30	0,306671	0,772	0,654	8,18E-26	3
Ptpn6	2,66E-30	0,397124	0,318	0,201	8,27E-26	3
Gnas	2,7E-30	0,291668	0,795	0,689	8,4E-26	3
Tap2	4,4E-30	0,341249	0,457	0,317	1,37E-25	3
Psmb3	4,97E-30	0,347898	0,587	0,449	1,54E-25	3
Tgoln1	5,96E-30	0,332232	0,382	0,247	1,85E-25	3
Cotl1	6,06E-30	0,323103	0,707	0,564	1,88E-25	3
Med28	8,13E-30	0,364769	0,414	0,283	2,52E-25	3
Lypla2	9,2E-30	0,359691	0,395	0,266	2,86E-25	3
Cox6b1	1,18E-29	0,326815	0,687	0,569	3,68E-25	3
Atp5h	1,23E-29	0,280585	0,827	0,735	3,81E-25	3
Psme2	1,61E-29	0,306596	0,769	0,635	5E-25	3
Chmp2a	2,32E-29	0,325694	0,343	0,218	7,22E-25	3
Srrm2	2,76E-29	0,298472	0,778	0,678	8,56E-25	3
Cycs	2,85E-29	0,381155	0,544	0,411	8,85E-25	3
Edf1	3,13E-29	0,311905	0,641	0,499	9,72E-25	3
Lcp2	4,06E-29	0,333712	0,467	0,323	1,26E-24	3
Pstpip1	5,51E-29	0,318518	0,38	0,25	1,71E-24	3
Usp48	6,35E-29	0,328373	0,266	0,156	1,97E-24	3
Higd1a	6,68E-29	0,342627	0,378	0,251	2,07E-24	3
Cox6a1	6,9E-29	0,317328	0,703	0,583	2,14E-24	3
Ndufa1	6,96E-29	0,315359	0,585	0,444	2,16E-24	3
Dusp5	3,05E-28	0,460103	0,593	0,471	9,47E-24	3
Pomp	4,1E-28	0,366938	0,502	0,371	1,27E-23	3
Dbnl	6,61E-28	0,358829	0,378	0,254	2,05E-23	3
Tma7	7,64E-28	0,304086	0,722	0,59	2,37E-23	3
Fam102a	8,34E-28	0,324964	0,401	0,267	2,59E-23	3
Ifi27	8,87E-28	0,352265	0,423	0,293	2,76E-23	3
Fam173a	1,06E-27	0,321347	0,312	0,197	3,28E-23	3
Pkn1	1,31E-27	0,343804	0,399	0,27	4,06E-23	3
Slc38a1	1,49E-27	0,350866	0,332	0,213	4,64E-23	3
Trappc6b	2,58E-27	0,316962	0,27	0,161	8E-23	3
Ier3ip1	3,5E-27	0,36415	0,449	0,323	1,09E-22	3
Cuta	3,52E-27	0,363627	0,401	0,28	1,09E-22	3
Polr2g	3,84E-27	0,308263	0,268	0,161	1,19E-22	3
Dynlrb1	4,71E-27	0,316278	0,411	0,282	1,46E-22	3
Cyfip2	8,59E-27	0,345384	0,412	0,286	2,67E-22	3
Ap3s1	1,39E-26	0,316079	0,269	0,162	4,31E-22	3
Actr2	2,31E-26	0,351247	0,477	0,351	7,17E-22	3
Arpc5	2,75E-26	0,313302	0,571	0,441	8,53E-22	3
Trir	3,16E-26	0,329141	0,494	0,362	9,81E-22	3
Bloc1s1	4,26E-26	0,329274	0,425	0,296	1,32E-21	3
Atf4	4,49E-26	0,345067	0,551	0,418	1,4E-21	3
Usmg5	6,86E-26	0,317346	0,758	0,648	2,13E-21	3
Cd47	9,88E-26	0,283287	0,708	0,579	3,07E-21	3
Aak1	1,09E-25	0,324111	0,284	0,177	3,4E-21	3
Pak2	1,28E-25	0,347101	0,479	0,355	3,98E-21	3
Mien1	1,32E-25	0,312199	0,344	0,227	4,1E-21	3
Atp5k	1,99E-25	0,284237	0,608	0,482	6,19E-21	3
Psmb10	2,48E-25	0,341168	0,467	0,34	7,69E-21	3
Tapbp	3,37E-25	0,30265	0,542	0,416	1,05E-20	3

Tm9sf3	3,8E-25	0,298108	0,306	0,194	1,18E-20	3
Chmp1a	3,86E-25	0,308519	0,3	0,191	1,2E-20	3
Was	4,82E-25	0,329052	0,334	0,221	1,5E-20	3
Mrpl18	6,5E-25	0,306974	0,323	0,212	2,02E-20	3
Ppp1ca	8,92E-25	0,27581	0,738	0,615	2,77E-20	3
Arpc2	9,57E-25	0,257077	0,875	0,795	2,97E-20	3
Prdx2	1,13E-24	0,308017	0,356	0,238	3,51E-20	3
Tor1aip1	1,34E-24	0,339677	0,259	0,159	4,16E-20	3
Tmem258	1,64E-24	0,307883	0,567	0,44	5,08E-20	3
Cox7a2	1,67E-24	0,308057	0,655	0,54	5,2E-20	3
Atp6v0b	2,05E-24	0,284738	0,317	0,203	6,36E-20	3
Csk	2,19E-24	0,313505	0,474	0,353	6,79E-20	3
Fkbp3	3,15E-24	0,311924	0,472	0,349	9,77E-20	3
Swi5	3,86E-24	0,316913	0,415	0,298	1,2E-19	3
Pet100	3,92E-24	0,314501	0,346	0,234	1,22E-19	3
Nedd8	4,22E-24	0,287517	0,52	0,394	1,31E-19	3
Gpx4	4,9E-24	0,314844	0,598	0,48	1,52E-19	3
Acap1	6E-24	0,325657	0,453	0,334	1,86E-19	3
Hectd1	6,07E-24	0,315454	0,421	0,302	1,88E-19	3
Itpr2	9,05E-24	0,325095	0,312	0,205	2,81E-19	3
Nfatc1	1,1E-23	0,283573	0,277	0,173	3,4E-19	3
Grcc10	1,2E-23	0,288782	0,742	0,621	3,73E-19	3
Atp2a3	2,01E-23	0,296173	0,318	0,209	6,26E-19	3
Sh2d1a	2,48E-23	0,28667	0,339	0,225	7,7E-19	3
Gpsm3	3,23E-23	0,30087	0,614	0,493	1E-18	3
Cwc15	3,36E-23	0,302376	0,441	0,322	1,04E-18	3
Mob1a	4,54E-23	0,293455	0,257	0,16	1,41E-18	3
Cdc37	4,73E-23	0,289607	0,517	0,394	1,47E-18	3
Commd7	5,29E-23	0,306484	0,255	0,158	1,64E-18	3
Stub1	5,83E-23	0,285974	0,273	0,173	1,81E-18	3
Uba1	9,21E-23	0,276861	0,357	0,243	2,86E-18	3
Csnk1g2	1,08E-22	0,296999	0,291	0,187	3,35E-18	3
Drap1	1,28E-22	0,28936	0,312	0,206	3,97E-18	3
Lta4h	1,8E-22	0,276497	0,296	0,192	5,6E-18	3
Inpp5d	1,86E-22	0,271097	0,304	0,196	5,79E-18	3
Ier5	2,05E-22	0,348021	0,616	0,504	6,37E-18	3
Calr	2,12E-22	0,343484	0,522	0,407	6,59E-18	3
Sdf4	2,22E-22	0,283812	0,533	0,405	6,89E-18	3
Gnb1	2,33E-22	0,297542	0,385	0,269	7,22E-18	3
Eif5a	2,38E-22	0,251465	0,83	0,76	7,39E-18	3
Rab4b	3,12E-22	0,260402	0,275	0,174	9,69E-18	3
Ankrd13a	3,13E-22	0,287076	0,265	0,166	9,71E-18	3
Lman2	3,79E-22	0,293482	0,303	0,199	1,18E-17	3
Tbc1d10c	6,19E-22	0,3016	0,49	0,368	1,92E-17	3
Atxn2l	7,69E-22	0,320713	0,462	0,345	2,39E-17	3
Txn1	9,41E-22	0,254378	0,462	0,338	2,92E-17	3
Med10	1,16E-21	0,275082	0,33	0,222	3,6E-17	3
Ppp3cc	1,22E-21	0,292465	0,315	0,21	3,78E-17	3
Ndufb11	1,25E-21	0,251262	0,666	0,551	3,88E-17	3
Ndufs5	1,29E-21	0,279293	0,329	0,22	4E-17	3
Fam32a	1,36E-21	0,266946	0,306	0,202	4,21E-17	3
Nop10	1,93E-21	0,280419	0,618	0,493	5,98E-17	3
Fli1	2,94E-21	0,317365	0,282	0,186	9,14E-17	3
Rasgrp1	3,98E-21	0,318547	0,42	0,307	1,24E-16	3
Gstp1	5,52E-21	0,259154	0,645	0,534	1,71E-16	3

Vasp	5,69E-21	0,261051	0,555	0,428	1,77E-16	3
Cab39	6,59E-21	0,277311	0,282	0,185	2,05E-16	3
Slc9a3r1	6,69E-21	0,270287	0,458	0,339	2,08E-16	3
Sec61g	1,57E-20	0,253153	0,786	0,707	4,86E-16	3
Purb	1,59E-20	0,277804	0,259	0,165	4,92E-16	3
Cox17	2,45E-20	0,254471	0,473	0,355	7,59E-16	3
Cox7b	3,51E-20	0,287007	0,62	0,512	1,09E-15	3
Tspan32	3,63E-20	0,250716	0,331	0,223	1,13E-15	3
Gadd45b	3,95E-20	0,411928	0,263	0,172	1,23E-15	3
Fis1	4,9E-20	0,280001	0,59	0,474	1,52E-15	3
Igtp	6,41E-20	0,250804	0,26	0,164	1,99E-15	3
Akna	8,31E-20	0,277444	0,396	0,284	2,58E-15	3
Grb2	1,04E-19	0,267332	0,322	0,22	3,23E-15	3
Dpm3	1,18E-19	0,293171	0,473	0,362	3,67E-15	3
Arl6ip5	1,27E-19	0,295256	0,599	0,493	3,95E-15	3
Gimap9	1,31E-19	0,326863	0,355	0,254	4,07E-15	3
Leprotl1	1,45E-19	0,253747	0,553	0,437	4,51E-15	3
Prkacb	1,88E-19	0,268232	0,29	0,194	5,84E-15	3
Aurkaip1	3,32E-19	0,305152	0,361	0,261	1,03E-14	3
G3bp2	4,57E-19	0,295846	0,392	0,288	1,42E-14	3
Dock10	5,04E-19	0,286198	0,473	0,359	1,57E-14	3
Cltc	6,68E-19	0,275984	0,301	0,206	2,07E-14	3
Rab5c	9,41E-19	0,254939	0,3	0,203	2,92E-14	3
Ube2n	1,4E-18	0,256964	0,338	0,237	4,33E-14	3
Fosl2	1,47E-18	0,325521	0,292	0,199	4,57E-14	3
Lbh	1,62E-18	0,278513	0,344	0,245	5,03E-14	3
Atox1	4,15E-18	0,255208	0,465	0,355	1,29E-13	3
Ppp2r1a	4,39E-18	0,253815	0,335	0,239	1,36E-13	3
Uqcr11	4,48E-18	0,264497	0,427	0,324	1,39E-13	3
Pitpna	4,98E-18	0,263987	0,4	0,296	1,55E-13	3
Sri	6,76E-18	0,265816	0,573	0,463	2,1E-13	3
Wdr1	7,64E-18	0,269393	0,466	0,359	2,37E-13	3
Smdt1	1,65E-17	0,266504	0,489	0,381	5,14E-13	3
Romo1	1,78E-17	0,264718	0,434	0,334	5,54E-13	3
Cox20	1,82E-17	0,251313	0,374	0,275	5,65E-13	3
Actn4	2,16E-17	0,271543	0,312	0,217	6,7E-13	3
Anxa11	2,49E-17	0,269386	0,263	0,176	7,72E-13	3
Psmb5	2,51E-17	0,260212	0,394	0,292	7,79E-13	3
Ube2i	2,7E-17	0,286615	0,531	0,433	8,4E-13	3
Anapc11	3,01E-17	0,269886	0,363	0,264	9,34E-13	3
Eif4g1	3,55E-17	0,267841	0,336	0,243	1,1E-12	3
Psma2	5,09E-17	0,267407	0,568	0,472	1,58E-12	3
Ier2	5,5E-17	0,296839	0,687	0,601	1,71E-12	3
BC031181	7,74E-17	0,274742	0,32	0,227	2,4E-12	3
Abhd8	1,47E-16	0,268632	0,308	0,217	4,56E-12	3
Sept7	1,68E-16	0,252717	0,415	0,313	5,22E-12	3
Ugcg	3,51E-16	0,255271	0,364	0,269	1,09E-11	3
Tmem59	8,93E-16	0,25136	0,386	0,289	2,77E-11	3
Gm11808	1,33E-15	0,334269	0,923	0,891	4,13E-11	3
Ndufc1	1,64E-15	0,269264	0,326	0,239	5,08E-11	3
Ndufaf8	1,98E-15	0,266581	0,258	0,178	6,13E-11	3
Nme1	2,64E-15	0,256135	0,382	0,288	8,19E-11	3
Hdac7	4,21E-15	0,277482	0,294	0,211	1,31E-10	3
Ddx39b	5,93E-15	0,265915	0,493	0,392	1,84E-10	3
Cul3	8,19E-15	0,254864	0,252	0,174	2,54E-10	3

Akr1a1	1,38E-14	0,256927	0,406	0,311	4,29E-10	3
Wdr83os	1,84E-14	0,268085	0,399	0,306	5,72E-10	3
Polr2m	3,53E-14	0,25988	0,331	0,246	1,1E-09	3
Ndufb8	3,58E-14	0,266581	0,388	0,296	1,11E-09	3
Esyt1	5,57E-14	0,257658	0,401	0,309	1,73E-09	3
Ywhaq	6,98E-14	0,26382	0,392	0,304	2,17E-09	3
Rgs2	6,45E-11	0,276978	0,37	0,292	2E-06	3
Ly6c2	0	1,996958	0,824	0,179	0	4
Ccl5	0	1,196261	0,871	0,279	0	4
Ctla2a	1E-298	1,261728	0,501	0,129	3,2E-294	4
Nkg7	3,1E-220	0,687268	0,881	0,426	9,7E-216	4
Cst7	6,9E-163	0,775537	0,436	0,148	2,1E-158	4
Sidt1	6,8E-159	0,799504	0,487	0,182	2,1E-154	4
Il2rb	2,5E-149	0,602591	0,731	0,341	7,7E-145	4
Ifitm10	4,7E-129	0,570505	0,34	0,107	1,5E-124	4
Klrd1	3,2E-124	0,686784	0,52	0,223	1E-119	4
Ctsw	1,2E-123	0,66942	0,72	0,392	3,8E-119	4
Ifngr1	1,8E-99	0,678005	0,735	0,446	5,59E-95	4
Plac8	2,02E-96	0,569988	0,345	0,132	6,27E-92	4
Zyx	1,86E-90	0,534428	0,607	0,322	5,78E-86	4
Cd7	2,13E-90	0,559793	0,438	0,197	6,61E-86	4
Rps5	1,17E-86	0,27961	0,991	0,994	3,63E-82	4
Gem	4,85E-86	0,551116	0,266	0,094	1,51E-81	4
Rpl13	1,09E-79	0,252053	0,995	0,997	3,4E-75	4
Socs3	1,39E-77	0,532794	0,311	0,127	4,32E-73	4
Dennd4a	2,54E-74	0,553629	0,461	0,232	7,9E-70	4
Rps12	1,27E-71	0,385103	0,98	0,96	3,93E-67	4
Bcl2	1,21E-66	0,524444	0,619	0,381	3,75E-62	4
Pglyrp1	6,6E-65	0,417637	0,332	0,147	2,05E-60	4
Cd8a	2,24E-64	0,356509	0,451	0,223	6,95E-60	4
Sell	1,29E-62	0,440975	0,626	0,375	4,01E-58	4
H2afz	2,18E-59	0,420936	0,826	0,652	6,76E-55	4
Epsti1	6,58E-57	0,475255	0,628	0,397	2,04E-52	4
Cd8b1	1,35E-56	0,411263	0,546	0,308	4,19E-52	4
Dnajc15	1,47E-55	0,442775	0,602	0,376	4,57E-51	4
Nr4a2	1,75E-53	0,434139	0,262	0,115	5,44E-49	4
Nsg2	1,11E-51	0,425689	0,476	0,267	3,43E-47	4
Rpl23a	3,39E-51	0,305239	0,947	0,906	1,05E-46	4
Hopx	7E-48	0,289627	0,453	0,245	2,17E-43	4
Cd69	2,21E-46	0,468504	0,637	0,439	6,85E-42	4
Rpl36a	2,69E-45	0,257132	0,976	0,965	8,35E-41	4
Rnf138	1,21E-44	0,457611	0,551	0,355	3,76E-40	4
Ptpn22	8,72E-44	0,411041	0,488	0,296	2,71E-39	4
Glipr2	7,84E-34	0,341167	0,334	0,191	2,43E-29	4
Tmem243	3,45E-32	0,336843	0,27	0,145	1,07E-27	4
Neurl3	2,17E-31	0,330744	0,305	0,172	6,73E-27	4
Gramd3	4,33E-31	0,345198	0,671	0,495	1,35E-26	4
Traf1	2,17E-30	0,388985	0,44	0,284	6,73E-26	4
Slc25a4	2,24E-30	0,285724	0,411	0,253	6,97E-26	4
Stat4	1,39E-29	0,302585	0,325	0,191	4,32E-25	4
Mif	2,62E-29	0,311406	0,742	0,599	8,13E-25	4
Gm26917	8,1E-28	0,377161	0,674	0,525	2,51E-23	4
Arl4c	7,97E-27	0,280623	0,527	0,358	2,47E-22	4
Pde7a	1,83E-26	0,317537	0,313	0,188	5,68E-22	4
Zc3h12a	1,93E-25	0,306673	0,396	0,256	6E-21	4

Kdm6b	7,21E-24	0,300744	0,439	0,294	2,24E-19	4
Nme2	2,54E-23	0,269115	0,848	0,764	7,88E-19	4
Nfkbiz	3,43E-23	0,262773	0,353	0,226	1,07E-18	4
Chd7	9,16E-23	0,274454	0,384	0,249	2,84E-18	4
Emb	1,12E-22	0,295751	0,675	0,518	3,47E-18	4
Scaf11	6,11E-22	0,638845	0,569	0,447	1,9E-17	4
Hspe1	1,53E-21	0,255092	0,727	0,594	4,75E-17	4
Pim2	2,08E-21	0,250361	0,279	0,169	6,46E-17	4
Gimap5	4,71E-20	0,281787	0,589	0,436	1,46E-15	4
Tgif1	6,74E-19	0,262511	0,353	0,236	2,09E-14	4
Trmt112	8,03E-19	0,257752	0,682	0,545	2,49E-14	4
S100a6	0	2,104784	0,693	0,205	0	5
Itgb1	0	2,097821	0,622	0,171	0	5
S100a4	0	2,084696	0,475	0,08	0	5
Tnfrsf4	0	1,567445	0,325	0,031	0	5
Ly6a	0	1,536511	0,652	0,222	0	5
Ahnak	6.0573283	1,439007	0,718	0,27	1,9E-303	5
S100a10	1,3E-306	1,609973	0,881	0,603	4,2E-302	5
S100a11	5,3E-298	1,571168	0,782	0,397	1,7E-293	5
Maf	2,8E-274	1,212508	0,347	0,064	8,7E-270	5
Lgals1	7,3E-267	1,622322	0,734	0,349	2,3E-262	5
H2-D1	1,6E-238	0,704064	0,995	0,982	5E-234	5
Cd4	1,2E-229	1,172267	0,558	0,194	3,8E-225	5
Cish	2,5E-211	1,227787	0,357	0,088	7,9E-207	5
Cd40lg	1,1E-210	1,347585	0,447	0,139	3,5E-206	5
Bhlhe40	6,1E-203	1,599078	0,492	0,168	1,9E-198	5
Prr13	4,4E-195	1,076066	0,616	0,274	1,4E-190	5
H2-K1	5,8E-194	0,670149	0,994	0,981	1,8E-189	5
Tmsb4x	1,9E-191	0,625235	0,984	0,995	5,8E-187	5
Gata3	4,4E-171	1,157192	0,442	0,158	1,4E-166	5
Capg	5,2E-168	1,121475	0,312	0,082	1,6E-163	5
Ctla4	2,2E-161	1,542551	0,336	0,099	6,9E-157	5
Crip1	2,3E-161	1,499358	0,872	0,736	7,1E-157	5
Vim	2,6E-158	1,546693	0,673	0,406	8,1E-154	5
Rgs1	4,7E-156	1,581713	0,58	0,283	1,4E-151	5
Samsn1	1E-151	1,032353	0,391	0,133	3,2E-147	5
AU020206	8,3E-148	0,932207	0,375	0,125	2,6E-143	5
Cd44	8,8E-146	0,902941	0,392	0,135	2,7E-141	5
Rbpj	1,1E-144	0,957966	0,267	0,069	3,4E-140	5
Zfp36	4,4E-144	1,833635	0,697	0,446	1,4E-139	5
Srgn	4,4E-140	0,844975	0,934	0,849	1,4E-135	5
Bcl2a1b	1,2E-137	0,96729	0,431	0,166	3,7E-133	5
AW11201(	9,3E-136	0,903924	0,851	0,618	2,9E-131	5
Cxcr3	7,4E-134	0,885405	0,331	0,106	2,3E-129	5
Rora	2,9E-131	0,828866	0,291	0,083	9,1E-127	5
Junb	9,5E-127	0,996173	0,97	0,959	2,9E-122	5
Glrx	6,7E-126	0,863094	0,271	0,078	2,1E-121	5
S100a13	5,9E-124	0,909524	0,622	0,363	1,8E-119	5
Fosb	6,5E-118	1,308995	0,47	0,219	2E-113	5
Tnfrsf18	5,2E-116	0,969606	0,444	0,196	1,6E-111	5
Laptm5	2,3E-114	0,652035	0,901	0,791	7,3E-110	5
Cd82	4,1E-114	0,889902	0,562	0,293	1,3E-109	5
CAAA011	4,3E-112	0,833481	0,346	0,13	1,3E-107	5
Ndfip1	1,5E-108	0,836244	0,72	0,512	4,7E-104	5
Acot7	4,6E-108	0,689402	0,263	0,081	1,4E-103	5

Ccr2	7,7E-108	0,86067	0,254	0,076	2,4E-103	5
Actb	5E-106	0,579668	0,989	0,989	1,6E-101	5
Itm2b	9,3E-104	0,642722	0,9	0,825	2,9E-99	5
Gadd45b	2,4E-103	1,439815	0,374	0,162	7,4E-99	5
Lsp1	8,6E-102	0,838485	0,778	0,609	2,66E-97	5
Nfkbid	1,99E-98	0,986865	0,37	0,156	6,18E-94	5
Actg1	3,11E-93	0,773375	0,939	0,928	9,67E-89	5
Ly6e	2,33E-91	0,594736	0,894	0,792	7,22E-87	5
Serinc3	4,31E-90	0,857442	0,57	0,342	1,34E-85	5
Sla	6,81E-89	0,769609	0,402	0,187	2,12E-84	5
Btg2	1,28E-88	1,131617	0,637	0,435	3,97E-84	5
Cd5	1,4E-88	0,960464	0,507	0,287	4,33E-84	5
Id2	5,22E-88	0,879878	0,608	0,37	1,62E-83	5
Ucp2	5,89E-88	0,677595	0,799	0,647	1,83E-83	5
Nfkbiz	1,62E-87	1,08211	0,428	0,219	5,04E-83	5
Jund	4,28E-87	0,783133	0,948	0,899	1,33E-82	5
Rac2	1,2E-84	0,464274	0,946	0,91	3,73E-80	5
Ctsb	1,14E-83	0,747089	0,442	0,225	3,54E-79	5
Anxa6	3,69E-82	0,726737	0,615	0,409	1,15E-77	5
Smco4	1,35E-81	0,569409	0,25	0,089	4,2E-77	5
Myl6	1,51E-81	0,515053	0,916	0,876	4,68E-77	5
Sh3bgrl3	6,52E-80	0,53743	0,901	0,861	2,02E-75	5
Itga4	9,55E-79	0,896051	0,485	0,276	2,97E-74	5
Pkp3	8,19E-76	0,713843	0,361	0,171	2,54E-71	5
Egr1	7,22E-75	1,998978	0,365	0,187	2,24E-70	5
Atp2b1	7,62E-75	0,761106	0,414	0,214	2,37E-70	5
Malat1	2,76E-73	0,752421	0,994	0,984	8,57E-69	5
Icos	1,72E-72	0,814708	0,374	0,186	5,34E-68	5
Gm2a	2,74E-72	0,679559	0,345	0,162	8,51E-68	5
Ppp1r12a	1,02E-71	0,662405	0,604	0,389	3,17E-67	5
Hmgb2	1,24E-71	0,767314	0,664	0,501	3,85E-67	5
H2-Q7	1,47E-71	0,580468	0,887	0,813	4,58E-67	5
Pglyrp1	5,88E-71	0,757204	0,323	0,149	1,83E-66	5
Ttc39b	1,68E-69	0,619734	0,265	0,107	5,22E-65	5
Myo1f	3,44E-69	0,560376	0,251	0,097	1,07E-64	5
Ptpn7	4,96E-69	0,611018	0,331	0,151	1,54E-64	5
Gapdh	7,38E-68	0,521541	0,924	0,904	2,29E-63	5
Dusp1	5,28E-67	0,971348	0,69	0,54	1,64E-62	5
Rinl	6,23E-67	0,647849	0,495	0,291	1,94E-62	5
Itgb7	3,04E-66	0,719205	0,685	0,528	9,43E-62	5
Clic1	2,25E-65	0,572526	0,76	0,629	6,98E-61	5
Cd2	7,42E-65	0,638661	0,685	0,513	2,3E-60	5
Ifi27l2a	4,96E-63	1,263074	0,658	0,541	1,54E-58	5
Plk3	1,34E-62	0,729607	0,305	0,144	4,18E-58	5
Ldha	2,03E-62	0,613163	0,771	0,653	6,31E-58	5
Nfkb1	2,04E-61	0,691074	0,398	0,217	6,35E-57	5
Lcp2	1,83E-60	0,628854	0,508	0,321	5,68E-56	5
Shisa5	7,6E-60	0,42977	0,942	0,908	2,36E-55	5
Hif1a	1,16E-59	0,729772	0,387	0,212	3,59E-55	5
Tagln2	1,58E-59	0,71313	0,719	0,594	4,9E-55	5
Smap1	5,24E-59	0,637027	0,474	0,291	1,63E-54	5
B2m	5,63E-59	0,330841	0,982	0,979	1,75E-54	5
Sept9	1,35E-58	0,633922	0,535	0,349	4,18E-54	5
Il2rg	1,52E-58	0,538656	0,836	0,749	4,73E-54	5
Sorl1	5,93E-58	0,668544	0,304	0,147	1,84E-53	5

Itgal	1,66E-57	0,594141	0,517	0,324	5,17E-53	5
Psen2	3,5E-57	0,577377	0,271	0,122	1,09E-52	5
Cd28	3,64E-57	0,713981	0,639	0,478	1,13E-52	5
Nr4a1	4,98E-57	0,995459	0,59	0,432	1,55E-52	5
Il2rb	8,76E-57	0,376056	0,599	0,358	2,72E-52	5
H2-Q6	2,13E-55	0,603593	0,716	0,553	6,6E-51	5
Tspo	1,35E-54	0,578912	0,682	0,534	4,2E-50	5
Gng2	1,51E-54	0,506791	0,253	0,111	4,68E-50	5
Tmbim6	8,02E-54	0,52856	0,7	0,559	2,49E-49	5
Ywhaz	1,37E-53	0,44713	0,857	0,781	4,27E-49	5
Spn	2,83E-53	0,638682	0,385	0,218	8,79E-49	5
Gnb2	4,85E-52	0,564782	0,639	0,488	1,51E-47	5
Ier2	4,97E-52	0,938419	0,716	0,599	1,54E-47	5
Apobec3	1,12E-51	0,590976	0,393	0,227	3,48E-47	5
Ass1	2,63E-51	0,764971	0,457	0,29	8,18E-47	5
Cd52	4,34E-51	0,445665	0,941	0,923	1,35E-46	5
Lrp10	1,14E-50	0,594305	0,415	0,25	3,53E-46	5
Grap	1,89E-50	0,594978	0,458	0,291	5,86E-46	5
Lcp1	2,91E-50	0,550783	0,705	0,576	9,04E-46	5
Gnai2	4,5E-50	0,514306	0,758	0,674	1,4E-45	5
Emp3	5,98E-50	0,607908	0,644	0,503	1,86E-45	5
Sdf4	8,52E-50	0,591953	0,569	0,403	2,64E-45	5
Fos	9,92E-50	1,028134	0,559	0,408	3,08E-45	5
Hopx	1,9E-49	0,518035	0,429	0,249	5,89E-45	5
Ubb	2,5E-49	0,31455	0,983	0,988	7,77E-45	5
Traf1	3,89E-48	0,695383	0,444	0,284	1,21E-43	5
Calm1	4,05E-48	0,455613	0,863	0,806	1,26E-43	5
Vamp8	5,6E-48	0,596549	0,462	0,298	1,74E-43	5
Nek7	6,41E-48	0,52917	0,277	0,136	1,99E-43	5
Thy1	3,46E-47	0,483685	0,832	0,745	1,07E-42	5
Pfn1	4,28E-47	0,340726	0,975	0,968	1,33E-42	5
Arpc1b	7,8E-47	0,479049	0,798	0,716	2,42E-42	5
Hcst	2,85E-45	0,505324	0,843	0,786	8,86E-41	5
Fyb	3,51E-45	0,513436	0,672	0,529	1,09E-40	5
Ppp1r18	9,33E-45	0,489609	0,707	0,576	2,9E-40	5
Cxcr6	2,33E-44	0,535159	0,302	0,153	7,24E-40	5
Fkbp1a	4,24E-44	0,529926	0,55	0,396	1,32E-39	5
H2-T23	8,18E-43	0,500996	0,719	0,583	2,54E-38	5
Syngr2	1,17E-42	0,467129	0,251	0,123	3,65E-38	5
Ier5	2,15E-42	0,781313	0,622	0,505	6,69E-38	5
Malt1	2,72E-42	0,639611	0,308	0,17	8,45E-38	5
Coro1a	8,06E-42	0,355324	0,941	0,929	2,5E-37	5
Iqgap1	9,78E-42	0,583263	0,515	0,369	3,04E-37	5
Smpdl3a	2,3E-41	0,589662	0,368	0,224	7,13E-37	5
Mrpl33	2,66E-41	0,5313	0,541	0,4	8,27E-37	5
Ubc	1,2E-40	0,448195	0,872	0,843	3,74E-36	5
Arhgdib	1,22E-40	0,360832	0,901	0,884	3,8E-36	5
Reep5	1,8E-40	0,488457	0,477	0,321	5,6E-36	5
Cyba	2,63E-40	0,407089	0,756	0,646	8,15E-36	5
Cd48	3,28E-40	0,598626	0,372	0,229	1,02E-35	5
Ifng	3,56E-40	0,786603	0,25	0,126	1,11E-35	5
Cdc42	3,71E-40	0,484915	0,733	0,659	1,15E-35	5
Cfl1	4,43E-40	0,358984	0,929	0,923	1,38E-35	5
Runx1	6,18E-40	0,505366	0,324	0,181	1,92E-35	5
Adgre5	6,58E-40	0,524888	0,596	0,445	2,04E-35	5

Ppp1ca	1,02E-39	0,455059	0,712	0,619	3,17E-35	5
Nr3c1	4,61E-39	0,573506	0,3	0,167	1,43E-34	5
Lat	1,22E-38	0,374925	0,852	0,795	3,79E-34	5
Txn1	3E-38	0,474379	0,483	0,337	9,31E-34	5
Aldoa	4,21E-37	0,472572	0,679	0,567	1,31E-32	5
Ddx5	7,66E-37	0,339905	0,96	0,951	2,38E-32	5
Myl12b	1,46E-36	0,367153	0,864	0,827	4,53E-32	5
Psmb8	1,55E-36	0,412886	0,763	0,666	4,81E-32	5
Nfkbia	2,24E-35	0,885285	0,776	0,751	6,94E-31	5
Pla2g16	3,9E-35	0,464092	0,368	0,228	1,21E-30	5
Lpcat4	5,05E-35	0,517532	0,29	0,166	1,57E-30	5
Tgfb1	6,69E-35	0,562107	0,434	0,299	2,08E-30	5
Itm2c	1,11E-34	0,543581	0,337	0,207	3,44E-30	5
Gabarapl2	1,51E-34	0,462063	0,589	0,456	4,7E-30	5
Cd6	2,27E-34	0,611521	0,43	0,3	7,03E-30	5
Cd3e	4E-34	0,308207	0,917	0,894	1,24E-29	5
Prelid1	5,05E-34	0,445524	0,572	0,45	1,57E-29	5
Oaz1	8,67E-34	0,279148	0,937	0,931	2,69E-29	5
Pim1	1,99E-33	0,62655	0,605	0,472	6,19E-29	5
Elob	2,16E-33	0,368396	0,736	0,645	6,71E-29	5
Cox17	2,18E-33	0,434219	0,498	0,354	6,79E-29	5
Myo1g	2,31E-33	0,526189	0,317	0,194	7,17E-29	5
Ets1	2,57E-33	0,430787	0,784	0,692	7,99E-29	5
Tmem50a	4,51E-33	0,410413	0,639	0,522	1,4E-28	5
2010111I0	5,06E-33	0,477356	0,306	0,177	1,57E-28	5
H3f3b	6,41E-33	0,441284	0,979	0,977	1,99E-28	5
Cap1	6,78E-33	0,466868	0,495	0,357	2,11E-28	5
Hivep2	7,74E-33	0,500855	0,294	0,17	2,4E-28	5
Sit1	1,24E-32	0,463972	0,318	0,192	3,86E-28	5
Capns1	1,32E-32	0,436231	0,447	0,31	4,11E-28	5
Arpc3	1,39E-32	0,380176	0,756	0,677	4,31E-28	5
Ms4a4b	1,5E-32	0,509171	0,756	0,718	4,67E-28	5
Faah	1,22E-31	0,47812	0,263	0,149	3,78E-27	5
Hnrnpa2b'	2,09E-31	0,371636	0,819	0,759	6,5E-27	5
Mbnl1	4,1E-31	0,431373	0,768	0,697	1,27E-26	5
Xist	7,82E-31	0,463364	0,665	0,526	2,43E-26	5
Prdx2	2,17E-30	0,44284	0,364	0,239	6,75E-26	5
Gimap1	4,66E-30	0,430499	0,579	0,455	1,45E-25	5
Grb2	7,83E-30	0,435263	0,343	0,219	2,43E-25	5
Dok2	1,25E-29	0,422332	0,323	0,198	3,87E-25	5
Cd47	2,09E-29	0,408625	0,673	0,585	6,5E-25	5
Samhd1	2,17E-29	0,578437	0,517	0,399	6,72E-25	5
Anxa5	2,85E-29	0,415506	0,263	0,151	8,83E-25	5
Tspan13	2,87E-29	0,549025	0,395	0,273	8,9E-25	5
Arpc4	5,75E-29	0,443423	0,582	0,479	1,79E-24	5
Cd37	5,99E-29	0,447583	0,619	0,51	1,86E-24	5
Tbc1d10c	9,39E-29	0,443488	0,498	0,369	2,91E-24	5
Tsc22d4	1,16E-28	0,423461	0,475	0,339	3,61E-24	5
Tnfaip3	1,18E-28	0,698209	0,765	0,751	3,68E-24	5
BC031181	1,23E-28	0,435669	0,347	0,225	3,81E-24	5
Actr3	2,09E-28	0,344152	0,758	0,682	6,48E-24	5
Pdia3	3,57E-28	0,434639	0,566	0,457	1,11E-23	5
Ptp4a2	7,86E-28	0,435611	0,608	0,5	2,44E-23	5
Esyt1	1,01E-27	0,465134	0,431	0,307	3,14E-23	5
Csrnp1	1,56E-27	0,551882	0,462	0,346	4,83E-23	5

Flna	1,58E-27	0,529016	0,501	0,387	4,9E-23	5
Glipr2	1,64E-27	0,447705	0,309	0,194	5,11E-23	5
Arf4	1,84E-27	0,514108	0,571	0,468	5,71E-23	5
Neurl3	3,1E-27	0,601102	0,284	0,175	9,63E-23	5
Sec11c	3,69E-27	0,418965	0,473	0,349	1,15E-22	5
Arpc5	4,07E-27	0,430389	0,548	0,445	1,26E-22	5
Gna13	7,76E-27	0,522839	0,519	0,398	2,41E-22	5
Cd3g	1,06E-26	0,304598	0,863	0,845	3,28E-22	5
Gimap4	1,28E-26	0,33697	0,704	0,604	3,98E-22	5
Dusp5	1,76E-26	0,731423	0,57	0,475	5,48E-22	5
Dad1	3,84E-26	0,389179	0,634	0,54	1,19E-21	5
Tpst2	6,43E-26	0,418238	0,413	0,291	2E-21	5
Ppp1r15a	7,11E-26	0,486299	0,737	0,67	2,21E-21	5
Edf1	8,23E-26	0,349984	0,613	0,504	2,55E-21	5
H2-Q4	8,31E-26	0,439471	0,758	0,694	2,58E-21	5
Fam107b	1,01E-25	0,393264	0,672	0,58	3,13E-21	5
Clec2d	1,05E-25	0,412584	0,385	0,259	3,25E-21	5
Ppib	1,15E-25	0,378888	0,67	0,6	3,56E-21	5
Ctsd	1,48E-25	0,413234	0,54	0,425	4,6E-21	5
Itgb2	2E-25	0,378399	0,532	0,409	6,2E-21	5
Arl6ip1	3,24E-25	0,41734	0,515	0,401	1,01E-20	5
Fli1	4E-25	0,402538	0,298	0,186	1,24E-20	5
Ccng1	4,3E-25	0,415315	0,297	0,187	1,33E-20	5
Cnot6l	7,69E-25	0,425187	0,31	0,198	2,39E-20	5
Klf6	1,08E-24	0,645015	0,622	0,547	3,35E-20	5
Cdk11b	1,29E-24	0,556701	0,484	0,377	4E-20	5
Nfatc1	1,33E-24	0,474183	0,276	0,174	4,13E-20	5
Tm9sf3	1,89E-24	0,393098	0,306	0,196	5,87E-20	5
Wipf1	1,96E-24	0,415389	0,389	0,273	6,08E-20	5
Atpif1	2,51E-24	0,431418	0,262	0,162	7,8E-20	5
Ptprcap	4,97E-24	0,401713	0,698	0,631	1,54E-19	5
Ubl5	7,71E-24	0,346875	0,682	0,586	2,39E-19	5
Arpc2	1,74E-23	0,302877	0,823	0,801	5,4E-19	5
Calm3	1,75E-23	0,392429	0,408	0,298	5,42E-19	5
AC149090	1,92E-23	0,423653	0,392	0,276	5,95E-19	5
Dock2	2E-23	0,485746	0,523	0,427	6,22E-19	5
Inpp5d	2,21E-23	0,430518	0,305	0,198	6,86E-19	5
Pycard	2,69E-23	0,501142	0,364	0,265	8,36E-19	5
Cox6b1	2,85E-23	0,353306	0,653	0,574	8,84E-19	5
Gmfg	3,16E-23	0,414418	0,65	0,58	9,81E-19	5
Atox1	4,25E-23	0,381997	0,469	0,356	1,32E-18	5
Runx3	5,23E-23	0,383217	0,312	0,206	1,62E-18	5
Sft2d1	6,82E-23	0,34372	0,289	0,184	2,12E-18	5
Akap13	7,49E-23	0,387401	0,704	0,621	2,32E-18	5
Lrrfip1	8,6E-23	0,414466	0,385	0,275	2,67E-18	5
Gpx4	1,16E-22	0,392707	0,574	0,484	3,6E-18	5
Cst3	1,25E-22	0,410657	0,297	0,193	3,87E-18	5
Ptpn1	1,26E-22	0,416102	0,345	0,238	3,92E-18	5
Fnbp1	1,42E-22	0,372572	0,364	0,254	4,42E-18	5
Arhgdia	1,62E-22	0,378259	0,604	0,516	5,04E-18	5
Pstpip1	5,74E-22	0,349208	0,367	0,253	1,78E-17	5
Arhgap30	6,13E-22	0,409027	0,405	0,298	1,9E-17	5
Psmb9	1,33E-21	0,391352	0,454	0,344	4,14E-17	5
Ccnl1	2,33E-21	0,436271	0,592	0,501	7,25E-17	5
Ctsa	2,37E-21	0,380101	0,31	0,207	7,36E-17	5

Zc3h12a	3,98E-21	0,48304	0,361	0,261	1,23E-16	5
Cotl1	6,38E-21	0,407605	0,638	0,574	1,98E-16	5
Ppp2ca	6,56E-21	0,375302	0,403	0,293	2,04E-16	5
Gpsm3	1,12E-20	0,334362	0,59	0,498	3,49E-16	5
Fxyd5	1,18E-20	0,263718	0,899	0,891	3,67E-16	5
Capzb	1,36E-20	0,33417	0,654	0,584	4,22E-16	5
Ppp4c	1,67E-20	0,352772	0,496	0,398	5,19E-16	5
Cdipt	2,21E-20	0,39564	0,325	0,224	6,87E-16	5
Spcs2	2,28E-20	0,38639	0,424	0,316	7,08E-16	5
Ndufa13	2,84E-20	0,295672	0,677	0,596	8,82E-16	5
Prrc2b	3,1E-20	0,377431	0,353	0,246	9,62E-16	5
Gimap7	3,12E-20	0,426777	0,347	0,243	9,68E-16	5
S1pr4	3,33E-20	0,397759	0,26	0,168	1,03E-15	5
H2afz	4,25E-20	0,459433	0,715	0,665	1,32E-15	5
Fmnl1	4,26E-20	0,368318	0,478	0,374	1,32E-15	5
Rap1a	4,33E-20	0,355414	0,45	0,346	1,34E-15	5
Bcap31	4,98E-20	0,347735	0,367	0,263	1,55E-15	5
Atp6v0e	6,07E-20	0,360127	0,428	0,321	1,88E-15	5
Fryl	7,35E-20	0,422628	0,317	0,216	2,28E-15	5
Nfatc3	7,51E-20	0,42036	0,353	0,254	2,33E-15	5
Gnb1	8,04E-20	0,339491	0,379	0,271	2,5E-15	5
1110008P	8,4E-20	0,309181	0,253	0,157	2,61E-15	5
Myl12a	1,96E-19	0,311043	0,704	0,656	6,09E-15	5
Cox6c	2,09E-19	0,266713	0,775	0,722	6,5E-15	5
Rps6ka1	2,15E-19	0,349754	0,28	0,184	6,68E-15	5
Irf2bp2	2,17E-19	0,392719	0,266	0,175	6,72E-15	5
Zgpat	2,59E-19	0,361541	0,342	0,239	8,06E-15	5
Ccnd2	4,57E-19	0,354294	0,547	0,438	1,42E-14	5
Zap70	4,84E-19	0,352021	0,432	0,326	1,5E-14	5
Odc1	5,52E-19	0,741892	0,415	0,327	1,72E-14	5
Cdc42se1	7,9E-19	0,352505	0,413	0,313	2,45E-14	5
Srpr	1,18E-18	0,344743	0,261	0,171	3,67E-14	5
Kmt2e	1,24E-18	0,381341	0,496	0,4	3,86E-14	5
Snx3	1,53E-18	0,336774	0,438	0,339	4,75E-14	5
Srrm2	1,93E-18	0,330984	0,742	0,683	5,98E-14	5
Plec	2,34E-18	0,385881	0,302	0,207	7,28E-14	5
Mndal	3,14E-18	0,371825	0,402	0,307	9,76E-14	5
Hspa5	4,21E-18	0,419851	0,747	0,707	1,31E-13	5
Sash3	4,27E-18	0,332248	0,345	0,248	1,33E-13	5
Prex1	4,43E-18	0,364552	0,338	0,24	1,38E-13	5
Cd69	4,82E-18	0,594593	0,537	0,451	1,5E-13	5
Lypla2	4,85E-18	0,359418	0,367	0,27	1,51E-13	5
Pbxip1	5,92E-18	0,347634	0,295	0,203	1,84E-13	5
Slc38a1	6,84E-18	0,384571	0,311	0,217	2,12E-13	5
Ubl3	7,2E-18	0,410442	0,338	0,244	2,24E-13	5
Hcls1	1,31E-17	0,344822	0,379	0,285	4,07E-13	5
Hint1	2,19E-17	0,334897	0,649	0,603	6,79E-13	5
Ndufb7	2,3E-17	0,329982	0,452	0,356	7,15E-13	5
Pomp	3,37E-17	0,303731	0,465	0,376	1,05E-12	5
Dynlrb1	3,69E-17	0,298372	0,383	0,286	1,15E-12	5
Kcnn4	4,7E-17	0,354782	0,294	0,202	1,46E-12	5
Stk24	6,55E-17	0,345594	0,447	0,353	2,03E-12	5
Mxd4	7,54E-17	0,305848	0,269	0,18	2,34E-12	5
Rab1b	9,63E-17	0,300996	0,333	0,238	2,99E-12	5
Cyth4	1,78E-16	0,316245	0,306	0,214	5,53E-12	5

Aprt	2,7E-16	0,358648	0,396	0,306	8,37E-12	5
Hmgb1	2,77E-16	0,279859	0,744	0,692	8,59E-12	5
Tln1	2,96E-16	0,330008	0,517	0,423	9,2E-12	5
Ankrd12	6,4E-16	0,400965	0,274	0,19	1,99E-11	5
Arf1	6,42E-16	0,287611	0,566	0,486	1,99E-11	5
Vps28	7,98E-16	0,315516	0,509	0,424	2,48E-11	5
Cnn2	9,46E-16	0,276601	0,734	0,688	2,94E-11	5
Ifi203	1,02E-15	0,395152	0,43	0,342	3,15E-11	5
Tap1	1,04E-15	0,320158	0,366	0,275	3,22E-11	5
Tgoln1	1,05E-15	0,328903	0,343	0,253	3,27E-11	5
Coro1b	1,32E-15	0,348401	0,341	0,254	4,1E-11	5
Sema4d	1,46E-15	0,342877	0,322	0,237	4,55E-11	5
Prkar1a	2,48E-15	0,296702	0,547	0,467	7,69E-11	5
Atrx	2,5E-15	0,335515	0,3	0,214	7,76E-11	5
Batf	2,68E-15	0,374857	0,254	0,176	8,31E-11	5
Cox5a	2,81E-15	0,289415	0,567	0,489	8,72E-11	5
M6pr	2,87E-15	0,349699	0,324	0,24	8,9E-11	5
Slc9a3r1	3,02E-15	0,334091	0,43	0,344	9,39E-11	5
Ywhab	3,17E-15	0,29972	0,434	0,344	9,84E-11	5
Mrps21	5,19E-15	0,29541	0,392	0,305	1,61E-10	5
Rab7	5,84E-15	0,305325	0,346	0,253	1,81E-10	5
Med28	6,55E-15	0,274779	0,38	0,288	2,03E-10	5
Srebf2	9,07E-15	0,305099	0,253	0,173	2,82E-10	5
Hsp90b1	9,79E-15	0,349787	0,48	0,4	3,04E-10	5
Selplg	1,09E-14	0,274726	0,72	0,677	3,37E-10	5
Usmg5	1,13E-14	0,270704	0,705	0,655	3,52E-10	5
Tmem59	1,14E-14	0,293216	0,384	0,291	3,55E-10	5
Sri	1,34E-14	0,291319	0,543	0,467	4,15E-10	5
Crlf2	1,4E-14	0,353081	0,251	0,174	4,36E-10	5
Atp5j2	1,46E-14	0,252846	0,703	0,655	4,53E-10	5
Dock8	1,48E-14	0,353769	0,302	0,219	4,59E-10	5
Polr1d	1,6E-14	0,284768	0,647	0,593	4,97E-10	5
Ak2	1,63E-14	0,275755	0,279	0,195	5,05E-10	5
Gabarap	2,23E-14	0,295849	0,576	0,505	6,93E-10	5
Aes	2,51E-14	0,250242	0,654	0,6	7,79E-10	5
Ptprc	2,59E-14	0,284059	0,82	0,784	8,04E-10	5
Lamtor4	2,93E-14	0,271728	0,276	0,195	9,11E-10	5
Actr2	2,97E-14	0,316205	0,439	0,357	9,23E-10	5
Celf2	3,01E-14	0,310856	0,44	0,351	9,33E-10	5
Srp14	3,03E-14	0,278475	0,467	0,377	9,4E-10	5
Bax	3,15E-14	0,295962	0,413	0,325	9,78E-10	5
Ccdc88c	3,88E-14	0,336303	0,343	0,262	1,21E-09	5
Ndufa3	4,44E-14	0,256241	0,706	0,663	1,38E-09	5
Pttg1	4,66E-14	0,289189	0,297	0,212	1,45E-09	5
Scamp3	5,36E-14	0,296746	0,309	0,227	1,66E-09	5
Rpn2	6,41E-14	0,340426	0,321	0,242	1,99E-09	5
Sem1	6,67E-14	0,251759	0,708	0,67	2,07E-09	5
D8Ertd738	6,73E-14	0,31102	0,631	0,562	2,09E-09	5
Cltc	6,98E-14	0,330911	0,288	0,208	2,17E-09	5
Bin2	7,19E-14	0,298467	0,614	0,555	2,23E-09	5
Lman2	7,51E-14	0,289172	0,284	0,202	2,33E-09	5
Psenen	9,49E-14	0,294642	0,465	0,383	2,95E-09	5
Fermt3	1,07E-13	0,2712	0,344	0,257	3,33E-09	5
Ddost	1,19E-13	0,299815	0,321	0,241	3,68E-09	5
Son	1,35E-13	0,308998	0,72	0,67	4,19E-09	5

Rock1	1,58E-13	0,291973	0,323	0,239	4,91E-09	5
Bsg	1,59E-13	0,300142	0,372	0,287	4,95E-09	5
Tprgl	1,92E-13	0,276642	0,481	0,394	5,97E-09	5
Cyb5a	2,2E-13	0,288401	0,354	0,271	6,83E-09	5
Cyld	2,8E-13	0,310476	0,257	0,18	8,68E-09	5
1810058I2	2,98E-13	0,273245	0,4	0,316	9,26E-09	5
Tmem30a	3,07E-13	0,287064	0,278	0,2	9,53E-09	5
Tapbp	3,63E-13	0,306497	0,5	0,422	1,13E-08	5
Ppp1cc	4,71E-13	0,309844	0,547	0,481	1,46E-08	5
Bloc1s1	4,75E-13	0,29215	0,383	0,302	1,47E-08	5
Ppp2r5c	4,77E-13	0,296685	0,28	0,201	1,48E-08	5
Csnk2b	4,8E-13	0,284146	0,449	0,368	1,49E-08	5
Fis1	5,66E-13	0,257822	0,551	0,48	1,76E-08	5
Arf6	5,76E-13	0,314227	0,538	0,474	1,79E-08	5
Tnfaip8	6,03E-13	0,296024	0,255	0,179	1,87E-08	5
1810037I1	6,78E-13	0,251132	0,428	0,348	2,1E-08	5
Maz	9,32E-13	0,283872	0,294	0,215	2,9E-08	5
Sytl3	1,08E-12	0,399256	0,251	0,179	3,34E-08	5
Tpm4	1,12E-12	0,30847	0,29	0,212	3,48E-08	5
Akna	1,18E-12	0,287868	0,37	0,289	3,68E-08	5
Os9	1,23E-12	0,279053	0,273	0,196	3,83E-08	5
Def6	1,24E-12	0,26369	0,343	0,259	3,87E-08	5
Nptn	1,29E-12	0,31626	0,253	0,18	4E-08	5
Ubxn4	1,38E-12	0,288911	0,294	0,217	4,28E-08	5
Tma7	1,51E-12	0,250937	0,656	0,598	4,7E-08	5
Stat3	1,52E-12	0,327774	0,318	0,238	4,73E-08	5
Trir	1,53E-12	0,273382	0,448	0,368	4,75E-08	5
Atp6v0b	1,58E-12	0,263497	0,286	0,208	4,92E-08	5
Tmem258	1,81E-12	0,256803	0,514	0,447	5,62E-08	5
Tap2	1,96E-12	0,269835	0,41	0,324	6,1E-08	5
Clk1	2,01E-12	0,377093	0,655	0,608	6,25E-08	5
Atp2a3	2,09E-12	0,283773	0,291	0,213	6,5E-08	5
Lnpep	2,41E-12	0,341401	0,335	0,259	7,5E-08	5
Birc3	2,43E-12	0,347928	0,268	0,193	7,54E-08	5
Dctn6	2,53E-12	0,279666	0,296	0,218	7,87E-08	5
Sptssa	2,62E-12	0,260068	0,366	0,285	8,12E-08	5
Ap2s1	2,75E-12	0,267372	0,289	0,212	8,53E-08	5
Gpr132	3E-12	0,398032	0,499	0,434	9,32E-08	5
Zdhhc18	3,32E-12	0,275343	0,293	0,217	1,03E-07	5
Abhd17a	3,35E-12	0,276793	0,258	0,185	1,04E-07	5
Tmem14c	3,39E-12	0,273314	0,342	0,263	1,05E-07	5
Ywhah	3,83E-12	0,269095	0,409	0,325	1,19E-07	5
Rab4b	3,96E-12	0,269849	0,25	0,178	1,23E-07	5
Top1	4,81E-12	0,326786	0,358	0,28	1,49E-07	5
Surf4	4,94E-12	0,292	0,257	0,185	1,53E-07	5
Kcnab2	5,43E-12	0,286984	0,302	0,227	1,69E-07	5
Gdi2	5,61E-12	0,292729	0,509	0,444	1,74E-07	5
Chmp2a	6,67E-12	0,304104	0,298	0,224	2,07E-07	5
Psma4	7,49E-12	0,262752	0,26	0,188	2,33E-07	5
Gm26740	7,52E-12	0,33788	0,277	0,205	2,34E-07	5
Wdr1	7,91E-12	0,263932	0,433	0,364	2,46E-07	5
Pkm	8,01E-12	0,270466	0,578	0,525	2,49E-07	5
Rsbn1l	8,46E-12	0,29033	0,344	0,268	2,63E-07	5
Sh2d2a	1,18E-11	0,307474	0,454	0,377	3,65E-07	5
Rab8a	1,19E-11	0,280231	0,398	0,321	3,7E-07	5

Taldo1	1,64E-11	0,260453	0,427	0,352	5,09E-07	5
Gsk3b	1,66E-11	0,284991	0,28	0,206	5,14E-07	5
Aph1a	1,93E-11	0,280173	0,26	0,187	5,98E-07	5
Hspa4	4,58E-11	0,299559	0,463	0,387	1,42E-06	5
Scand1	4,75E-11	0,289285	0,58	0,531	1,47E-06	5
Psmb5	5,39E-11	0,273491	0,37	0,296	1,67E-06	5
Inpp4b	5,51E-11	0,293479	0,407	0,328	1,71E-06	5
Tmem167	5,85E-11	0,270917	0,33	0,256	1,82E-06	5
Ghitm	7,59E-11	0,267032	0,381	0,303	2,36E-06	5
Cast	9,29E-11	0,287388	0,322	0,251	2,89E-06	5
Commd4	1,04E-10	0,261604	0,26	0,191	3,22E-06	5
Leprotl1	1,17E-10	0,288735	0,506	0,444	3,63E-06	5
Psmb2	1,44E-10	0,265762	0,362	0,292	4,46E-06	5
Aup1	1,44E-10	0,266006	0,457	0,387	4,47E-06	5
Tspan32	1,47E-10	0,271123	0,3	0,227	4,55E-06	5
Zc3hav1	1,61E-10	0,330121	0,528	0,466	4,98E-06	5
Zfp36l2	1,72E-10	0,327084	0,731	0,676	5,35E-06	5
Chmp4b	2,23E-10	0,251792	0,519	0,458	6,93E-06	5
Glud1	2,25E-10	0,333473	0,361	0,293	6,97E-06	5
Ifngr1	2,25E-10	0,316985	0,532	0,469	6,98E-06	5
Esyt2	2,38E-10	0,262678	0,272	0,204	7,4E-06	5
Zyx	2,52E-10	0,282551	0,41	0,344	7,82E-06	5
Dnajc3	3,26E-10	0,323329	0,411	0,346	1,01E-05	5
Sh3kbp1	3,79E-10	0,303188	0,399	0,334	1,18E-05	5
Vasp	4,06E-10	0,266766	0,496	0,436	1,26E-05	5
Ogt	4,94E-10	0,293143	0,269	0,202	1,53E-05	5
Spint2	4,99E-10	0,259854	0,255	0,19	1,55E-05	5
Dnajb6	5,2E-10	0,283549	0,385	0,315	1,61E-05	5
Actn4	5,34E-10	0,280028	0,288	0,221	1,66E-05	5
Mbp	6,47E-10	0,270077	0,273	0,21	2,01E-05	5
Cdc42se2	9,48E-10	0,27943	0,401	0,334	2,95E-05	5
Ddx24	1,44E-09	0,333633	0,474	0,415	4,46E-05	5
Hnrnpk	1,53E-09	0,260679	0,637	0,6	4,74E-05	5
R3hdm4	2,01E-09	0,252616	0,283	0,218	6,23E-05	5
Ythdc1	2,25E-09	0,294382	0,489	0,431	6,98E-05	5
Tmed5	2,87E-09	0,267353	0,281	0,218	8,9E-05	5
Ociad1	4E-09	0,266391	0,355	0,291	0,000124	5
Zfand6	4,87E-09	0,269876	0,288	0,225	0,000151	5
Sat1	4,9E-09	0,339021	0,391	0,328	0,000152	5
Csk	5,11E-09	0,250028	0,424	0,36	0,000159	5
Sqstm1	5,36E-09	0,32693	0,579	0,545	0,000166	5
Diaph1	7,69E-09	0,262903	0,299	0,239	0,000239	5
Vcp	7,9E-09	0,273464	0,367	0,304	0,000245	5
Ddx3x	7,96E-09	0,284158	0,482	0,422	0,000247	5
Ifrd1	8,77E-09	0,457303	0,484	0,446	0,000272	5
Slc1a5	1,09E-08	0,308443	0,305	0,245	0,000339	5
Prkca	1,13E-08	0,295254	0,258	0,201	0,000352	5
Nktr	1,26E-08	0,331867	0,304	0,24	0,000392	5
Rassf5	2,32E-08	0,251989	0,35	0,287	0,000721	5
Rexo2	2,66E-08	0,255556	0,337	0,278	0,000825	5
Mtdh	2,78E-08	0,252336	0,372	0,311	0,000863	5
Tnks2	3,58E-08	0,27751	0,268	0,211	0,001111	5
Zfp91	6,52E-08	0,263389	0,254	0,197	0,002026	5
Atf4	1,05E-07	0,269613	0,482	0,427	0,003253	5
Tra2a	1,14E-07	0,264867	0,378	0,319	0,003538	5

Cmtm7	1,45E-07	0,277917	0,268	0,215	0,004491	5
Vsir	1,48E-07	0,251092	0,358	0,303	0,004598	5
Fam102a	1,75E-07	0,252738	0,33	0,276	0,005428	5
Lbh	2,26E-07	0,265178	0,302	0,251	0,007026	5
Ywhae	3,11E-07	0,251817	0,361	0,308	0,009667	5
Itpkb	4,23E-07	0,304846	0,391	0,34	0,013131	5
Cd247	4,59E-07	0,328568	0,546	0,516	0,014261	5
Ltb	4,85E-07	0,251323	0,748	0,739	0,01506	5
Kdm6b	6,55E-07	0,319592	0,351	0,304	0,020353	5
Rgs2	9,42E-07	0,50736	0,335	0,297	0,02925	5
Gm42418	0	2,09319	1	0,999	0	6
Lars2	1,4E-202	1,571485	0,997	0,965	4,3E-198	6
Ccr7	2,46E-37	0,404201	0,726	0,522	7,65E-33	6
Gm26917	1,01E-20	0,682293	0,623	0,532	3,15E-16	6
Actn1	7,96E-20	0,307975	0,453	0,321	2,47E-15	6
Dusp10	5,28E-14	0,292151	0,514	0,412	1,64E-09	6
Rflnb	2,3E-12	0,278884	0,427	0,329	7,15E-08	6
Als2cl	1,14E-11	0,257675	0,252	0,173	3,54E-07	6
AY036118	0	2,393582	1	0,853	0	7
Rpl17	3E-144	0,451951	0,999	0,985	9,2E-140	7
Actn1	5,3E-123	0,897957	0,66	0,308	1,7E-118	7
Rps29	6,48E-81	0,305582	1	0,996	2,01E-76	7
Rps28	7,13E-79	0,317709	0,996	0,99	2,21E-74	7
Rps19	3,68E-76	0,360134	0,995	0,986	1,14E-71	7
Lef1	7,9E-73	0,572727	0,84	0,56	2,45E-68	7
Rps26	1,82E-71	0,333191	0,998	0,99	5,66E-67	7
Rps18	4,07E-70	0,340032	0,986	0,974	1,26E-65	7
Rps27	6,34E-69	0,287559	0,999	0,991	1,97E-64	7
Rplp1	1,26E-57	0,283717	0,997	0,992	3,91E-53	7
Rps23	4,37E-56	0,281893	0,991	0,981	1,36E-51	7
Rpl12	4,75E-55	0,314559	0,988	0,97	1,48E-50	7
Med21	5,93E-53	0,711127	0,306	0,137	1,84E-48	7
Nsg2	1,09E-50	0,533459	0,495	0,272	3,38E-46	7
Selenop	5,49E-48	0,544404	0,514	0,291	1,7E-43	7
Dapl1	1,1E-46	0,561294	0,412	0,213	3,41E-42	7
Rpl26	1,21E-45	0,257741	0,991	0,979	3,76E-41	7
Rps6	3,29E-45	0,308221	0,988	0,949	1,02E-40	7
Npm1	1,91E-44	0,355742	0,949	0,88	5,93E-40	7
Ccr7	2,04E-44	0,421355	0,779	0,522	6,34E-40	7
Rpl35	2,5E-44	0,259485	0,996	0,984	7,76E-40	7
Igfbp4	4,4E-39	0,45712	0,505	0,298	1,37E-34	7
mt-Nd6	8,47E-37	0,482093	0,403	0,227	2,63E-32	7
Klf3	7,98E-36	0,540746	0,592	0,394	2,48E-31	7
Myh9	6,43E-34	0,428407	0,829	0,684	2E-29	7
Cd55	1,04E-33	0,436703	0,274	0,134	3,24E-29	7
Rpl4	2,21E-32	0,263253	0,97	0,929	6,86E-28	7
Sell	1,37E-24	0,347778	0,568	0,388	4,26E-20	7
Cblb	4,58E-24	0,426568	0,384	0,243	1,42E-19	7
Emb	2,26E-23	0,343118	0,694	0,522	7,03E-19	7
Npc2	2,61E-23	0,297848	0,855	0,702	8,1E-19	7
Dusp10	1,72E-22	0,412263	0,575	0,409	5,33E-18	7
Bach2	5,82E-22	0,31173	0,352	0,213	1,81E-17	7
Tcf7	1,18E-21	0,265775	0,799	0,606	3,66E-17	7
Uimc1	2,34E-21	0,354058	0,288	0,169	7,25E-17	7
Gm2682	8,1E-21	0,2723	0,456	0,299	2,51E-16	7

Fam78a	1,39E-20	0,287004	0,306	0,181	4,32E-16	7
Cmah	6,48E-19	0,267151	0,298	0,18	2,01E-14	7
Il7r	1,46E-17	0,278585	0,661	0,518	4,53E-13	7
Rflnb	1,1E-16	0,257127	0,475	0,327	3,42E-12	7
Ranbp10	1,48E-16	0,296016	0,262	0,159	4,58E-12	7
Cd8b1	2,78E-16	0,283515	0,459	0,323	8,62E-12	7
Peli1	3,07E-16	0,252537	0,651	0,51	9,52E-12	7
Rgs10	6,66E-15	0,256599	0,583	0,44	2,07E-10	7
S1pr1	1,06E-14	0,268164	0,484	0,351	3,31E-10	7
Ccl5	0	4,488991	0,999	0,303	0	8
Gzma	0	3,847007	0,393	0,022	0	8
Ccl4	0	3,259473	0,503	0,045	0	8
Nkg7	0	2,491986	0,964	0,445	0	8
Gzmk	0	1,990422	0,377	0,006	0	8
Zeb2	0	1,673207	0,332	0,006	0	8
Cx3cr1	0	1,64062	0,317	0,002	0	8
Klrg1	0	1,407401	0,252	0,011	0	8
Plek	1,3E-269	1,257162	0,289	0,029	4,1E-265	8
Bhlhe40	6,6E-264	1,894188	0,657	0,174	2E-259	8
Ahnak	1,6E-244	1,896333	0,769	0,287	4,9E-240	8
Gzmb	1,5E-243	1,71407	0,416	0,07	4,7E-239	8
Lgals1	1E-227	2,088558	0,799	0,363	3,1E-223	8
S100a6	2,3E-209	1,449768	0,711	0,226	7,2E-205	8
Itga4	6,2E-184	1,567479	0,688	0,275	1,9E-179	8
Cd8b1	5E-170	1,671369	0,719	0,311	1,6E-165	8
S100a10	4,9E-157	1,216737	0,892	0,615	1,5E-152	8
H2-D1	9,3E-151	0,719988	0,992	0,983	2,9E-146	8
Fasl	4E-138	1,308754	0,325	0,074	1,2E-133	8
Nr4a2	8,9E-138	1,456026	0,411	0,114	2,8E-133	8
Hopx	9,3E-137	1,346274	0,604	0,248	2,9E-132	8
H2-Q7	4,2E-126	0,973042	0,929	0,814	1,3E-121	8
Cd8a	1,5E-124	1,346184	0,567	0,229	4,8E-120	8
Ifng	3,3E-122	1,297234	0,416	0,123	1E-117	8
Zyx	4,3E-122	1,277166	0,661	0,334	1,3E-117	8
Itgb1	1E-118	1,406527	0,519	0,197	3,2E-114	8
H2-K1	3E-116	0,626624	0,995	0,982	9,2E-112	8
Ctsd	6,9E-114	1,253429	0,707	0,422	2,1E-109	8
Anxa2	1,8E-112	1,07428	0,337	0,09	5,7E-108	8
Laptm5	2,7E-112	0,941122	0,908	0,796	8,4E-108	8
AW11201(	1,8E-111	1,043149	0,893	0,627	5,5E-107	8
S100a4	1,6E-108	0,99228	0,371	0,103	4,9E-104	8
Crip1	6,2E-107	0,993755	0,921	0,739	1,9E-102	8
Prr13	7,3E-107	1,17336	0,607	0,29	2,3E-102	8
H2afz	2,3E-101	1,044303	0,853	0,66	7,04E-97	8
Itgb2	1,6E-100	1,149811	0,688	0,406	4,93E-96	8
Klrk1	1,57E-97	1,263484	0,333	0,097	4,88E-93	8
Actg1	1,39E-92	0,782401	0,967	0,927	4,32E-88	8
Cd48	1,16E-91	1,147845	0,501	0,228	3,59E-87	8
Ifitm10	1,32E-90	1,078713	0,355	0,118	4,1E-86	8
Hcst	2,62E-90	0,875748	0,901	0,786	8,14E-86	8
Rpa2	5,56E-90	1,006978	0,308	0,092	1,73E-85	8
Klrd1	3,26E-89	1,320007	0,517	0,238	1,01E-84	8
Rap1b	6,05E-89	1,003487	0,849	0,717	1,88E-84	8
Actb	6,32E-85	0,634492	0,997	0,989	1,96E-80	8
Tbx21	1,23E-81	0,943088	0,276	0,08	3,83E-77	8

Ms4a4b	4,38E-79	0,918095	0,851	0,715	1,36E-74	8
Selplg	1,45E-76	0,885634	0,809	0,675	4,51E-72	8
Emp3	4,44E-76	1,061362	0,708	0,505	1,38E-71	8
Tmsb4x	7,67E-76	0,465176	0,996	0,994	2,38E-71	8
H2-Q6	3,35E-72	0,844328	0,776	0,557	1,04E-67	8
Cyba	9,46E-72	0,901931	0,797	0,649	2,94E-67	8
Spn	1,22E-71	1,048698	0,463	0,221	3,79E-67	8
Cst7	3,6E-70	0,98278	0,393	0,165	1,12E-65	8
Arpc1b	7,48E-69	0,734787	0,849	0,717	2,32E-64	8
Rac2	1,04E-68	0,577571	0,951	0,911	3,23E-64	8
S100a11	6,71E-68	0,926257	0,663	0,421	2,08E-63	8
Itgal	9,25E-67	0,975773	0,565	0,33	2,87E-62	8
Ikzf3	3,87E-59	0,867191	0,385	0,174	1,2E-54	8
Lsp1	5,2E-59	0,829458	0,768	0,617	1,62E-54	8
Anxa6	1,16E-55	0,864876	0,613	0,419	3,6E-51	8
Efhd2	4,11E-54	0,870398	0,448	0,236	1,28E-49	8
Ubb	6,28E-54	0,402236	0,992	0,987	1,95E-49	8
S100a13	7,06E-53	0,822638	0,575	0,377	2,19E-48	8
Cd3e	7,49E-53	0,559434	0,937	0,894	2,33E-48	8
Fyn	1,02E-52	0,879358	0,56	0,365	3,18E-48	8
Armc7	2,85E-52	0,909792	0,261	0,098	8,84E-48	8
Myo1f	8,99E-51	0,744424	0,269	0,103	2,79E-46	8
Ly6c2	6,81E-50	1,054968	0,455	0,232	2,11E-45	8
Lck	3,05E-49	0,621653	0,823	0,747	9,46E-45	8
Sh2d2a	2,02E-47	0,957386	0,555	0,375	6,27E-43	8
Pfn1	1,61E-46	0,435199	0,983	0,968	4,99E-42	8
Ier2	1,12E-45	0,809552	0,741	0,603	3,48E-41	8
Vim	2,71E-45	0,888285	0,607	0,422	8,42E-41	8
Thy1	8,92E-44	0,604993	0,839	0,749	2,77E-39	8
Bin2	9,29E-44	0,75125	0,685	0,554	2,88E-39	8
Ppp1r12a	3,56E-43	0,739708	0,588	0,4	1,11E-38	8
Ywhaq	3,63E-42	0,847171	0,483	0,305	1,13E-37	8
Id2	3,6E-41	0,753115	0,591	0,382	1,12E-36	8
Lat	6,41E-40	0,551393	0,855	0,797	1,99E-35	8
Cd52	3,62E-39	0,46414	0,947	0,924	1,12E-34	8
Gimap7	2,87E-38	0,755778	0,424	0,244	8,92E-34	8
Cox8a	4,91E-38	0,518771	0,861	0,801	1,52E-33	8
Srgn	5,87E-38	0,508959	0,911	0,854	1,82E-33	8
Calm1	8,39E-38	0,539919	0,851	0,809	2,6E-33	8
Runx3	9,39E-37	0,83968	0,373	0,207	2,92E-32	8
Ostf1	1,24E-36	0,718887	0,567	0,412	3,85E-32	8
Sp100	1,34E-36	0,758739	0,604	0,465	4,17E-32	8
Myl6	1,36E-36	0,447048	0,911	0,878	4,21E-32	8
Hmgb2	3,32E-35	0,77561	0,636	0,51	1,03E-30	8
Gtf2i	3,72E-35	0,818103	0,496	0,337	1,16E-30	8
Zfp36l2	6,04E-35	0,691603	0,773	0,677	1,87E-30	8
Bcl2a1b	1,45E-34	0,606431	0,352	0,182	4,52E-30	8
Reep5	4,28E-34	0,748643	0,484	0,328	1,33E-29	8
Ctla2a	1,69E-33	0,998554	0,311	0,158	5,24E-29	8
Lfng	1,73E-33	0,750862	0,335	0,181	5,38E-29	8
Cd82	2,39E-33	0,670169	0,484	0,309	7,43E-29	8
Sumo2	3,66E-33	0,573845	0,724	0,612	1,14E-28	8
H3f3b	4,06E-33	0,383393	0,987	0,977	1,26E-28	8
Nptn	7,56E-33	0,777696	0,329	0,179	2,35E-28	8
Tpst2	1,35E-32	0,752745	0,448	0,295	4,19E-28	8

Cox17	2,81E-32	0,730934	0,509	0,36	8,73E-28	8
Pycard	6,2E-32	0,778199	0,416	0,266	1,93E-27	8
Itm2b	8,58E-32	0,524183	0,845	0,831	2,67E-27	8
Arhgdib	1,87E-31	0,424551	0,899	0,885	5,8E-27	8
Atp5h	1,76E-30	0,535181	0,784	0,743	5,46E-26	8
Ppp1r18	1,19E-29	0,577793	0,681	0,583	3,7E-25	8
Il2rb	1,8E-29	0,397713	0,576	0,37	5,57E-25	8
Sytl3	6,8E-29	0,697892	0,324	0,178	2,11E-24	8
Rgs1	8,63E-29	0,664259	0,465	0,302	2,68E-24	8
Cyth4	1,5E-28	0,698271	0,361	0,215	4,65E-24	8
Flna	4,58E-28	0,701604	0,523	0,391	1,42E-23	8
Arl6ip5	1,6E-27	0,614766	0,603	0,499	4,98E-23	8
Ost4	3,66E-27	0,615793	0,639	0,541	1,14E-22	8
B2m	1,31E-26	0,26433	0,981	0,98	4,08E-22	8
Zgpat	2,78E-26	0,648578	0,379	0,242	8,64E-22	8
Ctsw	4,21E-25	0,55682	0,56	0,417	1,31E-20	8
Gimap5	4,82E-25	0,646587	0,563	0,445	1,5E-20	8
Pglyrp1	6,38E-25	0,640109	0,289	0,159	1,98E-20	8
Clic1	8,79E-25	0,4704	0,704	0,638	2,73E-20	8
H2-T23	1,71E-24	0,499216	0,673	0,592	5,32E-20	8
Tmbim6	1,96E-24	0,544767	0,652	0,568	6,08E-20	8
Ptprcap	2,88E-24	0,482733	0,721	0,633	8,95E-20	8
1810037I1	5,73E-24	0,666301	0,471	0,349	1,78E-19	8
Cfl1	1,94E-23	0,348691	0,931	0,923	6,04E-19	8
Ppp1ca	4,29E-23	0,516576	0,696	0,624	1,33E-18	8
Myl12a	4,54E-23	0,51057	0,703	0,658	1,41E-18	8
Capzb	5,13E-23	0,503974	0,663	0,587	1,59E-18	8
Gapdh	6,05E-23	0,358467	0,899	0,906	1,88E-18	8
Vamp8	3,05E-22	0,60303	0,431	0,308	9,46E-18	8
Ptprc	4,72E-22	0,452928	0,816	0,786	1,46E-17	8
Krtcap2	4,76E-22	0,642182	0,573	0,493	1,48E-17	8
Rnf166	8,66E-22	0,64507	0,384	0,259	2,69E-17	8
S1pr4	2,32E-21	0,687386	0,285	0,171	7,2E-17	8
Cd7	4,21E-21	0,634415	0,34	0,214	1,31E-16	8
Abracl	6,9E-21	0,603942	0,581	0,513	2,14E-16	8
Tbc1d10c	1,8E-20	0,594752	0,484	0,376	5,58E-16	8
Rasgrp2	2,66E-20	0,616031	0,516	0,414	8,27E-16	8
Cdc42	2,8E-20	0,51619	0,701	0,664	8,69E-16	8
Sub1	2,98E-20	0,462337	0,789	0,765	9,25E-16	8
Junb	4,08E-20	0,362769	0,965	0,959	1,27E-15	8
Smap1	1,15E-19	0,568628	0,421	0,302	3,56E-15	8
Jpt1	1,56E-19	0,615675	0,531	0,447	4,86E-15	8
Arpc2	3,84E-19	0,37785	0,797	0,803	1,19E-14	8
Tspo	4,57E-19	0,459282	0,627	0,543	1,42E-14	8
Ccnd3	5,04E-19	0,550626	0,539	0,44	1,57E-14	8
Lime1	5,34E-19	0,521936	0,271	0,162	1,66E-14	8
Iqgap1	7,07E-19	0,58484	0,48	0,378	2,2E-14	8
Dock2	1,34E-18	0,536232	0,529	0,431	4,15E-14	8
H2-Q4	2,32E-18	0,41884	0,74	0,697	7,21E-14	8
Arpc4	7,7E-18	0,493361	0,563	0,485	2,39E-13	8
Ifngr1	1,57E-17	0,510265	0,563	0,47	4,88E-13	8
Psmb8	1,8E-17	0,409352	0,7	0,673	5,59E-13	8
Gabarap	6,16E-17	0,42272	0,588	0,508	1,91E-12	8
Epsti1	6,65E-17	0,4782	0,519	0,415	2,06E-12	8
Gimap4	7,28E-17	0,400496	0,689	0,609	2,26E-12	8

Fxyd5	8,49E-17	0,351616	0,887	0,892	2,64E-12	8
Atp2a3	8,67E-17	0,560391	0,321	0,215	2,69E-12	8
Gabarapl2	1,42E-16	0,502924	0,548	0,465	4,42E-12	8
Cdc42se1	1,46E-16	0,533599	0,416	0,317	4,52E-12	8
Cnn2	2,23E-16	0,390183	0,719	0,691	6,93E-12	8
Fryl	2,84E-16	0,554088	0,321	0,221	8,81E-12	8
Dusp2	1,33E-15	0,580999	0,488	0,392	4,13E-11	8
Nlrc5	2,13E-15	0,498626	0,28	0,18	6,62E-11	8
Tmem50a	2,23E-15	0,490992	0,592	0,53	6,92E-11	8
Gnb2	2,29E-15	0,448203	0,571	0,498	7,11E-11	8
Ndfip1	4,86E-15	0,417228	0,597	0,528	1,51E-10	8
Cap1	5,12E-15	0,544066	0,451	0,366	1,59E-10	8
Myl12b	5,72E-15	0,302765	0,833	0,83	1,77E-10	8
Sema4a	1,74E-14	0,537852	0,252	0,16	5,42E-10	8
Rassf1	2,08E-14	0,562392	0,275	0,182	6,45E-10	8
Serf2	3,58E-14	0,279779	0,917	0,906	1,11E-09	8
Tnfaip3	7,65E-14	0,435529	0,789	0,75	2,38E-09	8
Fmnl1	8,49E-14	0,519991	0,459	0,38	2,64E-09	8
Glipr2	8,68E-14	0,481273	0,293	0,2	2,69E-09	8
Ccdc12	1,14E-13	0,489847	0,417	0,334	3,54E-09	8
Prex1	1,19E-13	0,549431	0,337	0,244	3,7E-09	8
Ndufa4	1,35E-13	0,358525	0,644	0,588	4,19E-09	8
Cox5b	1,87E-13	0,403073	0,649	0,631	5,79E-09	8
Arf6	2,69E-13	0,483743	0,54	0,477	8,36E-09	8
Sept1	2,97E-13	0,348493	0,761	0,732	9,22E-09	8
Adgre5	3,49E-13	0,416498	0,543	0,455	1,08E-08	8
Sh2d1a	3,58E-13	0,61296	0,323	0,233	1,11E-08	8
Cox6b1	3,93E-13	0,391505	0,627	0,579	1,22E-08	8
Bag1	4,02E-13	0,455984	0,373	0,288	1,25E-08	8
Gimap3	7,24E-13	0,301324	0,727	0,673	2,25E-08	8
Sap18	1,34E-12	0,399848	0,581	0,534	4,15E-08	8
Sertad1	1,35E-12	0,546657	0,376	0,287	4,18E-08	8
Tma7	1,36E-12	0,382451	0,636	0,602	4,21E-08	8
Gnai2	1,93E-12	0,369824	0,703	0,68	5,99E-08	8
Ptpn22	3,1E-12	0,424434	0,396	0,311	9,63E-08	8
Txn1	3,92E-12	0,480612	0,424	0,347	1,22E-07	8
Lcp1	4,29E-12	0,417225	0,631	0,586	1,33E-07	8
Ucp2	4,4E-12	0,390111	0,681	0,661	1,37E-07	8
Gnas	4,59E-12	0,402348	0,703	0,7	1,43E-07	8
Pstpip1	5,66E-12	0,465112	0,341	0,259	1,76E-07	8
Mkrn1	6,23E-12	0,508678	0,396	0,319	1,93E-07	8
Arl4c	7,01E-12	0,522993	0,444	0,371	2,18E-07	8
Pttg1	7,86E-12	0,509803	0,297	0,216	2,44E-07	8
Arpc5	1,03E-11	0,467391	0,507	0,452	3,19E-07	8
Nfatc1	1,17E-11	0,46253	0,263	0,18	3,63E-07	8
Atp6v1f	1,39E-11	0,407746	0,544	0,496	4,32E-07	8
Def6	1,68E-11	0,498921	0,344	0,262	5,21E-07	8
Ctsa	1,92E-11	0,478846	0,293	0,213	5,97E-07	8
Slc9a3r1	2,35E-11	0,447981	0,421	0,348	7,31E-07	8
Prrc2b	2,41E-11	0,484298	0,331	0,252	7,49E-07	8
Ccdc88c	2,95E-11	0,517236	0,343	0,266	9,15E-07	8
Ubc	4,43E-11	0,296581	0,835	0,846	1,37E-06	8
Actr3	4,87E-11	0,342501	0,705	0,689	1,51E-06	8
Elob	5,13E-11	0,297351	0,692	0,652	1,59E-06	8
Tuba1c	5,51E-11	0,435951	0,256	0,175	1,71E-06	8

Ywhaz	6,84E-11	0,298472	0,791	0,788	2,12E-06	8
Cd3g	9,51E-11	0,341878	0,84	0,847	2,95E-06	8
Sh3glb1	1,09E-10	0,440816	0,399	0,331	3,38E-06	8
Slc3a2	1,09E-10	0,515751	0,457	0,394	3,39E-06	8
Dad1	1,1E-10	0,345385	0,591	0,546	3,42E-06	8
Rab8a	1,52E-10	0,420941	0,395	0,325	4,72E-06	8
Chchd2	2E-10	0,257756	0,767	0,75	6,21E-06	8
Arhgdia	2,74E-10	0,391219	0,564	0,522	8,51E-06	8
Atox1	5,14E-10	0,442061	0,425	0,363	1,6E-05	8
Wipf1	5,3E-10	0,409342	0,359	0,28	1,65E-05	8
Calm2	6,32E-10	0,349759	0,568	0,531	1,96E-05	8
Grap	6,41E-10	0,384766	0,38	0,302	1,99E-05	8
Plec	6,58E-10	0,485847	0,287	0,212	2,04E-05	8
AC149090	6,66E-10	0,485179	0,356	0,283	2,07E-05	8
Dnajc15	8,65E-10	0,486196	0,449	0,396	2,69E-05	8
Nfatc3	8,89E-10	0,450618	0,335	0,259	2,76E-05	8
Brd9	1,11E-09	0,466036	0,253	0,183	3,45E-05	8
Arl6ip1	1,17E-09	0,416141	0,468	0,409	3,64E-05	8
Atp5j2	1,71E-09	0,266199	0,671	0,659	5,29E-05	8
Dek	1,95E-09	0,434124	0,405	0,341	6,05E-05	8
Bax	1,96E-09	0,400418	0,396	0,33	6,08E-05	8
Atp6v0e	2E-09	0,41147	0,396	0,328	6,21E-05	8
Hsp90b1	3,03E-09	0,416433	0,467	0,405	9,4E-05	8
AI413582	3,67E-09	0,399921	0,283	0,214	0,000114	8
Puf60	3,97E-09	0,390582	0,436	0,372	0,000123	8
Tpm3	4,04E-09	0,291254	0,676	0,657	0,000126	8
Pld3	4,71E-09	0,394112	0,256	0,186	0,000146	8
Pde7a	5,22E-09	0,432058	0,268	0,196	0,000162	8
Eno1	7,11E-09	0,329372	0,665	0,649	0,000221	8
Bsg	7,51E-09	0,45833	0,355	0,292	0,000233	8
Jak1	9,09E-09	0,404503	0,668	0,676	0,000282	8
Hnrnpl	9,46E-09	0,343953	0,556	0,512	0,000294	8
H2-T22	1,04E-08	0,35605	0,567	0,548	0,000322	8
Akna	1,18E-08	0,487318	0,355	0,293	0,000367	8
Arf1	1,23E-08	0,350863	0,531	0,491	0,000383	8
B4galnt1	1,43E-08	0,33145	0,613	0,597	0,000444	8
Atf4	1,55E-08	0,439199	0,479	0,43	0,000481	8
Dennd4a	2,29E-08	0,368866	0,324	0,251	0,00071	8
Sla	2,62E-08	0,430549	0,272	0,204	0,000813	8
Hnrnpa2b1	3,69E-08	0,253021	0,769	0,764	0,001145	8
Esyt1	3,81E-08	0,396406	0,38	0,315	0,001182	8
Csrnp1	4,62E-08	0,401315	0,417	0,354	0,001434	8
Lrp10	6,07E-08	0,376268	0,328	0,262	0,001885	8
Kmt2e	8,24E-08	0,391724	0,456	0,407	0,002558	8
Cox6c	8,46E-08	0,254711	0,732	0,727	0,002626	8
Myo1g	8,93E-08	0,41888	0,265	0,202	0,002773	8
Ets1	1,1E-07	0,271398	0,705	0,701	0,003428	8
Selenok	1,14E-07	0,295653	0,652	0,643	0,003546	8
Nr4a1	1,35E-07	0,327906	0,505	0,443	0,004179	8
1810058I2	1,35E-07	0,44595	0,372	0,321	0,004193	8
Klf2	1,55E-07	0,416331	0,773	0,747	0,004812	8
Tmed2	1,75E-07	0,378755	0,504	0,478	0,005444	8
Capns1	1,97E-07	0,381692	0,373	0,32	0,006104	8
Arl2bp	2,26E-07	0,444904	0,347	0,298	0,00703	8
Srp19	2,3E-07	0,414388	0,279	0,215	0,007154	8

Ptp4a2	2,58E-07	0,322372	0,539	0,509	0,008009	8
Klf3	2,87E-07	0,378265	0,46	0,405	0,008918	8
Irf2	3,86E-07	0,372006	0,279	0,216	0,011991	8
Dusp5	4,08E-07	0,310672	0,529	0,482	0,012663	8
Ybx1	4,16E-07	0,270953	0,697	0,705	0,012913	8
Mien1	4,4E-07	0,376322	0,293	0,237	0,013664	8
Ubl5	4,94E-07	0,323226	0,605	0,594	0,01535	8
Hcls1	6,72E-07	0,324089	0,348	0,291	0,020861	8
Trir	6,8E-07	0,38846	0,413	0,374	0,021121	8
Rasa3	6,91E-07	0,39789	0,301	0,246	0,021466	8
Hsp90aa1	7,05E-07	0,376689	0,564	0,561	0,021888	8
Tprgl	7,63E-07	0,345414	0,437	0,4	0,023698	8
Lamtor4	8,35E-07	0,371334	0,256	0,199	0,025929	8
Atp5j	8,36E-07	0,276805	0,601	0,599	0,025967	8
Inpp5d	8,54E-07	0,343758	0,267	0,205	0,026507	8
Slbp	8,6E-07	0,370261	0,291	0,231	0,026701	8
Tacc1	8,67E-07	0,402258	0,295	0,24	0,02693	8
Gtf2b	9,82E-07	0,376035	0,253	0,197	0,0305	8
Rinl	1,01E-06	0,358345	0,363	0,307	0,031253	8
Arhgap45	1,07E-06	0,27746	0,699	0,698	0,033236	8
Grb2	1,08E-06	0,342838	0,287	0,228	0,03342	8
Psmb3	1,08E-06	0,340229	0,489	0,462	0,033665	8
Aldoa	1,2E-06	0,315114	0,591	0,577	0,037328	8
M6pr	1,33E-06	0,328965	0,299	0,245	0,041286	8
Mrpl33	1,4E-06	0,399771	0,441	0,411	0,043337	8
Polr2a	1,42E-06	0,345558	0,337	0,285	0,044238	8
Sept9	1,44E-06	0,307462	0,413	0,364	0,044774	8
Hmgb1	1,56E-06	0,26358	0,703	0,696	0,048419	8
Trbv1	0	3,632203	1	0,04	0	9
Rps29	3,33E-33	0,277378	1	0,996	1,03E-28	9
Lef1	1,44E-26	0,556774	0,761	0,573	4,46E-22	9
Ccr7	9,43E-21	0,454983	0,753	0,532	2,93E-16	9
Rps19	1,11E-19	0,250376	0,998	0,987	3,46E-15	9
Igfbp4	1,66E-16	0,520925	0,48	0,306	5,17E-12	9
Actn1	1,61E-12	0,396818	0,476	0,327	4,99E-08	9
Npc2	3,48E-12	0,305779	0,805	0,71	1,08E-07	9
Als2cl	1,91E-10	0,369834	0,283	0,176	5,92E-06	9
Dusp10	1,58E-09	0,334434	0,546	0,416	4,9E-05	9
Rflnb	1,84E-09	0,340666	0,462	0,333	5,72E-05	9
Dapl1	2,06E-09	0,406674	0,333	0,223	6,4E-05	9
Rgcc	5,12E-09	0,317109	0,464	0,335	0,000159	9
Tcf7	7,09E-09	0,265024	0,757	0,614	0,00022	9
Nsg2	5,68E-08	0,257154	0,4	0,284	0,001764	9
Dgka	1,34E-06	0,252216	0,629	0,525	0,041531	9
Satb1	1,53E-06	0,279365	0,655	0,572	0,047588	9
Scaf11	1,4E-266	2,483148	1	0,444	4,3E-262	10
Rps8	1,29E-55	0,395422	1	0,996	4E-51	10
Rps20	2,99E-55	0,436066	1	0,995	9,27E-51	10
Rps28	2,61E-54	0,395398	1	0,99	8,11E-50	10
Rpsa	9,94E-53	0,3636	1	0,996	3,09E-48	10
Rpl13	9,59E-52	0,340734	1	0,997	2,98E-47	10
Rps15a	2,47E-51	0,356442	1	0,993	7,67E-47	10
Rpl41	8,44E-48	0,3073	1	0,996	2,62E-43	10
Rps7	8,9E-48	0,361333	1	0,993	2,76E-43	10
Rpl35a	4,98E-46	0,354241	1	0,993	1,55E-41	10

Rpl39	7E-46	0,338656	1	0,993	2,17E-41	10
Rpl32	8,11E-46	0,364186	1	0,99	2,52E-41	10
Cd8b1	4,47E-44	0,801423	0,65	0,323	1,39E-39	10
Rpl23	1,75E-43	0,321179	1	0,995	5,43E-39	10
Rps3a1	2,27E-43	0,330005	1	0,996	7,03E-39	10
Rplp1	5,1E-43	0,376169	1	0,992	1,58E-38	10
Rpl35	1,06E-42	0,381919	1	0,984	3,28E-38	10
Rplp0	1,35E-42	0,364637	1	0,993	4,19E-38	10
Rps24	3,36E-41	0,288397	1	0,997	1,04E-36	10
Rpl36a	7,51E-41	0,427271	0,998	0,965	2,33E-36	10
Rpl30	1,04E-40	0,3055	1	0,995	3,23E-36	10
Rps5	2,52E-39	0,325893	1	0,993	7,82E-35	10
Rps29	4E-39	0,324342	1	0,996	1,24E-34	10
Rpl15	2,1E-38	0,389482	1	0,977	6,54E-34	10
Rps4x	1,93E-36	0,320602	1	0,995	6E-32	10
Rps16	5,64E-35	0,273229	1	0,995	1,75E-30	10
Rps21	1,5E-34	0,284446	1	0,992	4,67E-30	10
Rps26	1,9E-33	0,334551	1	0,99	5,89E-29	10
Rps19	8,53E-33	0,35084	1	0,987	2,65E-28	10
Rps10	3,17E-32	0,274904	1	0,995	9,85E-28	10
Rpl8	7,8E-32	0,288503	1	0,993	2,42E-27	10
Rpl34	1,09E-31	0,263477	1	0,992	3,38E-27	10
Rpl28	1,98E-31	0,334554	1	0,985	6,14E-27	10
Rps2	3,48E-31	0,305957	1	0,993	1,08E-26	10
Rpl21	5,59E-31	0,275777	1	0,995	1,74E-26	10
Eef1b2	2,39E-30	0,372448	0,998	0,953	7,42E-26	10
Rpl36	2,63E-30	0,265586	1	0,992	8,18E-26	10
Rps27a	4,9E-30	0,263981	1	0,995	1,52E-25	10
Rpl27a	3,92E-29	0,269013	1	0,99	1,22E-24	10
Rpl37	1,11E-28	0,264231	1	0,995	3,45E-24	10
Rpl10a	1,13E-27	0,308754	0,998	0,978	3,5E-23	10
Rps3	1,31E-27	0,259756	0,998	0,992	4,08E-23	10
Rpl26	3,34E-26	0,306248	0,993	0,979	1,04E-21	10
Rpl6	3,48E-26	0,264721	1	0,994	1,08E-21	10
Rps27	5,54E-26	0,277528	1	0,992	1,72E-21	10
Rpl3	7,15E-26	0,304638	0,995	0,977	2,22E-21	10
Rplp2	1,05E-25	0,279932	1	0,991	3,25E-21	10
Rps18	1,32E-25	0,318201	0,998	0,975	4,09E-21	10
Dapl1	3,1E-25	0,602624	0,44	0,22	9,63E-21	10
Fam241a	3,14E-24	0,550405	0,319	0,141	9,74E-20	10
Rpl37a	4,76E-24	0,265632	1	0,992	1,48E-19	10
Rpl12	6,47E-24	0,307108	1	0,97	2,01E-19	10
Rpl5	1,47E-23	0,345393	0,995	0,952	4,55E-19	10
Cd8a	4,86E-23	0,460092	0,466	0,24	1,51E-18	10
Rps6	3,96E-21	0,306719	0,988	0,951	1,23E-16	10
Rack1	1,01E-20	0,311917	0,99	0,951	3,14E-16	10
Igfbp4	1,13E-20	0,545263	0,536	0,306	3,52E-16	10
Rpl13a	1,36E-19	0,274253	0,998	0,972	4,23E-15	10
Rps25	2,34E-19	0,339753	0,949	0,88	7,28E-15	10
Rpl7	4,89E-18	0,260543	0,998	0,967	1,52E-13	10
Rpl10	5,15E-18	0,252262	0,998	0,972	1,6E-13	10
Rpl14	1,63E-17	0,251054	0,99	0,973	5,05E-13	10
Rps12	3,69E-17	0,287648	0,998	0,961	1,15E-12	10
Rpl4	5,32E-16	0,277633	0,986	0,93	1,65E-11	10
Ccr7	3,54E-15	0,290961	0,773	0,533	1,1E-10	10

Rpl22l1	8,16E-15	0,296413	0,964	0,879	2,53E-10	10
Rpl23a	1,06E-14	0,29678	0,966	0,909	3,3E-10	10
Sell	3,36E-14	0,395688	0,582	0,395	1,04E-09	10
Npm1	1,04E-13	0,26747	0,954	0,883	3,24E-09	10
Rps15	1,96E-13	0,256111	0,983	0,933	6,08E-09	10
Nme2	2,7E-12	0,288294	0,884	0,769	8,4E-08	10
Lef1	8,29E-12	0,277607	0,756	0,574	2,58E-07	10
Rps17	6E-10	0,25721	0,915	0,836	1,86E-05	10
Peli1	1,38E-09	0,265185	0,667	0,516	4,28E-05	10
Hspe1	1,44E-09	0,296819	0,742	0,603	4,47E-05	10
Npc2	2,62E-08	0,266182	0,824	0,71	0,000813	10
Gm26917	2,3E-07	0,273328	0,664	0,537	0,007155	10
Trbv13-1	0	3,496294	1	0,057	0	11
Rpl39	8,34E-19	0,259356	1	0,993	2,59E-14	11
Rplp1	3,7E-18	0,303098	1	0,992	1,15E-13	11
Rps15a	1,37E-17	0,258104	1	0,993	4,25E-13	11
Rps7	2,15E-17	0,261125	1	0,993	6,67E-13	11
Rpl12	1,65E-16	0,329206	1	0,97	5,11E-12	11
Rpl35a	2,48E-16	0,250753	1	0,993	7,69E-12	11
Rpl36a	6,01E-14	0,280988	0,983	0,966	1,87E-09	11
Rps20	1,38E-13	0,255062	0,997	0,995	4,27E-09	11
Rps19	8,52E-13	0,268859	0,997	0,987	2,64E-08	11
Rpl35	8,85E-13	0,261758	0,993	0,984	2,75E-08	11
Rpl10a	9,03E-13	0,257881	0,997	0,978	2,81E-08	11
Eef1b2	1,48E-11	0,289487	0,979	0,953	4,59E-07	11
Igfbp4	7,82E-09	0,296041	0,483	0,309	0,000243	11
Npc2	8,74E-08	0,301837	0,81	0,711	0,002715	11
Rpl22l1	1,4E-07	0,290298	0,941	0,88	0,004342	11
Ifit3	0	1,719451	0,426	0,017	0	12
Ifit1	3,6E-298	2,146835	0,451	0,03	1,1E-293	12
Mx1	3,4E-231	1,18524	0,268	0,013	1,1E-226	12
Isg15	3,9E-208	2,551637	0,687	0,116	1,2E-203	12
Slfn5	2,5E-169	1,606865	0,419	0,048	7,7E-165	12
Rtp4	2,5E-163	1,758651	0,599	0,105	7,9E-159	12
Usp18	1,2E-160	1,191984	0,292	0,024	3,9E-156	12
Isg20	4,2E-151	1,485624	0,433	0,057	1,3E-146	12
Zbp1	8,7E-151	1,748745	0,669	0,142	2,7E-146	12
Rnf213	5,1E-124	1,903015	0,697	0,193	1,6E-119	12
Xaf1	6,4E-111	1,270568	0,44	0,078	2E-106	12
Parp14	4,7E-105	1,404632	0,525	0,116	1,5E-100	12
Oas3	9,9E-105	1,04987	0,271	0,031	3,1E-100	12
Igtp	1,1E-103	1,547024	0,62	0,166	3,5E-99	12
Gbp6	1,3E-103	0,992494	0,296	0,037	4,2E-99	12
Tgtp2	2,3E-103	1,381487	0,521	0,115	7,1E-99	12
Stat1	1,62E-98	1,676481	0,824	0,33	5,03E-94	12
Eif2ak2	1,51E-97	0,9108	0,278	0,035	4,69E-93	12
Irf7	2,88E-92	1,4224	0,546	0,141	8,93E-88	12
Ifit1bl1	8,18E-90	0,877988	0,261	0,033	2,54E-85	12
Bst2	7,02E-89	1,791227	0,694	0,258	2,18E-84	12
Trim30a	4,39E-87	1,366224	0,525	0,134	1,36E-82	12
Tgtp1	7,18E-85	1,073319	0,338	0,058	2,23E-80	12
Pml	3,11E-82	0,991464	0,377	0,073	9,65E-78	12
Ifih1	7,96E-80	0,872501	0,25	0,034	2,47E-75	12
Oas1a	1,51E-78	0,945954	0,254	0,036	4,68E-74	12
Ifi27l2a	1,54E-78	1,874014	0,898	0,545	4,77E-74	12

Phf11b	4,13E-78	1,338524	0,465	0,115	1,28E-73	12
Irgm1	3,06E-76	1,097944	0,38	0,079	9,49E-72	12
Ifi203	1,06E-73	1,403631	0,768	0,342	3,31E-69	12
Gbp4	9,25E-73	1,272866	0,546	0,164	2,87E-68	12
Gbp9	1,3E-72	1,04377	0,415	0,098	4,05E-68	12
Stat2	1,07E-70	0,835312	0,303	0,054	3,31E-66	12
Ifi213	3,31E-68	1,268785	0,539	0,169	1,03E-63	12
Gbp7	3,66E-65	1,131963	0,433	0,112	1,14E-60	12
Ifi208	1,44E-64	1,276358	0,486	0,145	4,46E-60	12
Ddx58	1,58E-64	1,127984	0,423	0,11	4,91E-60	12
Gbp2	2,15E-64	1,286058	0,37	0,087	6,68E-60	12
Slfn1	3,09E-64	1,272825	0,581	0,204	9,61E-60	12
Lgals3bp	1,35E-63	1,102083	0,44	0,121	4,19E-59	12
Ifi47	3,23E-63	1,433746	0,627	0,254	1E-58	12
Ly6a	7,08E-62	1,906842	0,637	0,255	2,2E-57	12
Slfn8	3,34E-57	0,994091	0,398	0,106	1,04E-52	12
Ifi206	6,22E-55	1,125793	0,437	0,128	1,93E-50	12
Dtx3l	5,09E-53	1,038497	0,412	0,119	1,58E-48	12
Parp9	1,12E-50	0,830969	0,299	0,069	3,47E-46	12
Herc6	1,25E-48	0,793653	0,278	0,062	3,89E-44	12
Samd9l	3,8E-48	0,969006	0,412	0,127	1,18E-43	12
Ifi214	2,37E-47	0,810629	0,289	0,068	7,37E-43	12
Samhd1	3,75E-45	1,37621	0,715	0,404	1,16E-40	12
Daxx	1,71E-43	1,006035	0,38	0,12	5,3E-39	12
Mndal	7,79E-43	1,153676	0,634	0,309	2,42E-38	12
Trafd1	2,6E-42	0,879722	0,296	0,077	8,07E-38	12
Ifi35	7,22E-42	1,010154	0,475	0,181	2,24E-37	12
Ifi209	2,91E-39	0,991114	0,451	0,172	9,05E-35	12
Nampt	4,54E-39	0,683416	0,268	0,067	1,41E-34	12
Helz2	7,56E-39	0,870366	0,384	0,128	2,35E-34	12
Ppa1	1,49E-37	0,757041	0,317	0,093	4,62E-33	12
H2-T23	2,13E-37	0,883771	0,842	0,591	6,62E-33	12
Tapbp	2,52E-37	1,010869	0,729	0,423	7,81E-33	12
Ly6e	6,06E-35	0,830577	0,937	0,799	1,88E-30	12
Psmb8	1,98E-32	0,839168	0,87	0,671	6,16E-28	12
Sp100	5,5E-31	0,898319	0,739	0,467	1,71E-26	12
H2-T22	3,55E-29	0,876897	0,785	0,544	1,1E-24	12
Shisa5	5,07E-26	0,575012	0,975	0,91	1,57E-21	12
Psme2	3,28E-25	0,701158	0,845	0,645	1,02E-20	12
Smchd1	7,77E-24	0,72556	0,475	0,232	2,41E-19	12
Zufsp	4,8E-23	0,636874	0,261	0,09	1,49E-18	12
Psmb10	2,69E-22	0,77993	0,592	0,348	8,35E-18	12
Gm4070	8,09E-22	0,593793	0,261	0,092	2,51E-17	12
Ccnd2	9,52E-22	0,782708	0,665	0,444	2,96E-17	12
Ms4a4b	9,98E-22	0,884987	0,849	0,719	3,1E-17	12
Irf9	1,03E-21	0,668714	0,331	0,138	3,19E-17	12
Nlrc5	1,04E-21	0,786135	0,391	0,181	3,24E-17	12
Gbp8	1,07E-21	0,592828	0,296	0,113	3,34E-17	12
Mitd1	1,62E-21	0,547107	0,275	0,101	5,03E-17	12
H2-K1	7,26E-21	0,42011	0,993	0,982	2,26E-16	12
Slfn2	1,07E-20	0,762692	0,63	0,401	3,32E-16	12
9930111J:	1,95E-20	0,659542	0,366	0,166	6,06E-16	12
Tap1	2,81E-20	0,680621	0,504	0,279	8,71E-16	12
Lgals9	3,4E-19	0,827425	0,5	0,298	1,06E-14	12
Tor1aip1	4,14E-19	0,626219	0,359	0,166	1,29E-14	12

Sp110	6,21E-19	0,634964	0,511	0,293	1,93E-14	12
B2m	7,49E-19	0,386387	0,982	0,98	2,33E-14	12
Ddx24	9,2E-19	0,664607	0,637	0,416	2,86E-14	12
Epsti1	6E-18	0,703277	0,634	0,416	1,86E-13	12
Rnf114	8,35E-18	0,548688	0,468	0,251	2,59E-13	12
Irf1	1,13E-17	0,784987	0,637	0,416	3,51E-13	12
Psmb9	1,3E-17	0,661388	0,567	0,35	4,04E-13	12
Psme1	1,4E-17	0,542201	0,835	0,711	4,36E-13	12
Adar	2,91E-17	0,558915	0,254	0,102	9,03E-13	12
Apobec3	4,71E-17	0,726836	0,437	0,239	1,46E-12	12
H2-D1	7,6E-17	0,34007	0,996	0,983	2,36E-12	12
Usp25	3,93E-16	0,693741	0,394	0,209	1,22E-11	12
Socs1	7E-16	0,782763	0,44	0,25	2,17E-11	12
AW11201(	4,77E-15	0,798086	0,771	0,638	1,48E-10	12
Nmi	1,14E-14	0,599972	0,37	0,193	3,54E-10	12
Ms4a6b	1,71E-14	0,521604	0,782	0,644	5,31E-10	12
Uba7	5,06E-14	0,54006	0,25	0,11	1,57E-09	12
H2-Q4	7,46E-14	0,483875	0,82	0,697	2,32E-09	12
Chmp4b	1,25E-13	0,61045	0,623	0,461	3,89E-09	12
Tmbim6	5,85E-13	0,516161	0,739	0,569	1,82E-08	12
Trim12a	1,41E-12	0,488346	0,352	0,192	4,37E-08	12
Stat3	3,33E-11	0,589447	0,401	0,243	1,03E-06	12
Ogfr	2,14E-10	0,467962	0,299	0,164	6,66E-06	12
H2-Q7	3,98E-10	0,348473	0,905	0,818	1,24E-05	12
Selenow	7,93E-10	0,376447	0,859	0,748	2,46E-05	12
Tap2	6,04E-09	0,410041	0,482	0,329	0,000188	12
Zc3hav1	1,13E-08	0,414731	0,62	0,469	0,000352	12
Dbnl	1,25E-08	0,405949	0,415	0,264	0,000389	12
Phip	2,69E-08	0,398923	0,306	0,181	0,000837	12
Cd47	4,18E-08	0,430045	0,718	0,59	0,001298	12
Gimap7	6,88E-08	0,347161	0,398	0,25	0,002137	12
Casp8	1,17E-07	0,333056	0,268	0,155	0,003648	12
Grina	1,8E-07	0,418855	0,261	0,151	0,005603	12
Ascc3	1,84E-07	0,327886	0,25	0,142	0,005723	12
H2-Q6	2,61E-07	0,371091	0,694	0,566	0,00812	12
Il2rg	3,77E-07	0,26601	0,856	0,755	0,011719	12
Cnp	5,07E-07	0,405678	0,384	0,266	0,015756	12
Plac8	6,79E-07	0,71189	0,25	0,151	0,021094	12
Tspo	7,17E-07	0,45663	0,658	0,545	0,022261	12
Clec2d	7,44E-07	0,369101	0,391	0,269	0,023111	12
Wipf1	1,37E-06	0,289969	0,412	0,281	0,042529	12
Tmem176	0	2,938563	0,848	0,038	0	13
Tmem176	0	2,85339	0,816	0,029	0	13
5830411N	0	2,321817	0,387	0,006	0	13
Pxdc1	0	2,299021	0,613	0,005	0	13
Blk	0	1,78131	0,47	0,001	0	13
Nr1d1	0	1,71207	0,493	0,015	0	13
Serpinb1a	0	1,606133	0,433	0,004	0	13
Ccr6	0	1,432074	0,392	0,002	0	13
Rorc	0	1,219679	0,355	0,001	0	13
Sdc1	0	1,111608	0,295	0,001	0	13
Il1r1	0	0,821453	0,258	0	0	13
Hk2	4,4E-306	1,466727	0,401	0,018	1,4E-301	13
S100a4	3,5E-304	2,784814	0,889	0,105	1,1E-299	13
Ltb4r1	3,5E-297	1,055083	0,35	0,013	1,1E-292	13

Lmo4	1,1E-278	2,474903	0,77	0,087	3,3E-274	13
Smox	2,8E-236	1,412497	0,47	0,034	8,7E-232	13
Aqp3	7,6E-222	1,330814	0,396	0,025	2,4E-217	13
Capg	8,1E-209	2,073189	0,71	0,094	2,5E-204	13
Actn2	1,8E-195	1,69938	0,553	0,059	5,5E-191	13
Igf1r	2,8E-188	1,215235	0,359	0,024	8,6E-184	13
Lmna	2,3E-184	1,367939	0,373	0,027	7,1E-180	13
S100a6	6,1E-180	2,901092	0,963	0,239	1,9E-175	13
Ramp3	2,9E-179	1,664038	0,553	0,063	9,1E-175	13
Scpep1	5,9E-170	0,930458	0,253	0,013	1,8E-165	13
Il18r1	1,4E-160	1,71638	0,668	0,105	4,4E-156	13
Tcrg-C1	6,5E-159	1,402422	0,424	0,04	2E-154	13
Lgals3	8,8E-156	1,956665	0,419	0,041	2,7E-151	13
Zbtb16	3,7E-148	1,073934	0,382	0,035	1,1E-143	13
Rora	4,9E-144	1,546959	0,618	0,095	1,5E-139	13
Ramp1	7,7E-140	1,949389	0,811	0,195	2,4E-135	13
Ly6g5b	5,2E-132	0,977731	0,304	0,025	1,6E-127	13
Fam129a	8,8E-119	1,053132	0,424	0,053	2,7E-114	13
Cxcr6	2,7E-118	1,548363	0,747	0,158	8,4E-114	13
Maf	3,7E-117	1,298887	0,535	0,084	1,1E-112	13
Nfil3	7,7E-116	1,544819	0,525	0,085	2,4E-111	13
Acsbg1	3,1E-114	1,337949	0,359	0,041	9,5E-110	13
S100a11	5,3E-110	2,040826	0,949	0,425	1,6E-105	13
Furin	2,5E-99	1,261253	0,507	0,089	7,66E-95	13
Ccr2	4,27E-95	1,23113	0,498	0,087	1,33E-90	13
Atf3	1,88E-94	1,795347	0,369	0,051	5,83E-90	13
Avpi1	7,56E-94	0,922581	0,272	0,028	2,35E-89	13
Fam110a	4,04E-93	0,918102	0,253	0,025	1,25E-88	13
Jaml	1,36E-92	1,30197	0,373	0,054	4,22E-88	13
Ckb	1,73E-91	1,398293	0,512	0,097	5,37E-87	13
Icos	1,78E-86	1,656394	0,687	0,196	5,53E-82	13
F2r	4,62E-85	1,155917	0,424	0,073	1,44E-80	13
Lgals1	1,33E-80	1,860938	0,88	0,377	4,13E-76	13
Il7r	1,96E-80	1,636208	0,977	0,522	6,08E-76	13
S100a10	1,82E-78	1,656183	0,963	0,624	5,67E-74	13
Rarg	6,14E-78	0,903927	0,281	0,035	1,91E-73	13
Dgat1	1,59E-74	1,098436	0,41	0,075	4,93E-70	13
Ikzf3	3,16E-74	1,389161	0,636	0,178	9,82E-70	13
Tnfrsf25	8,01E-72	1,110453	0,406	0,076	2,49E-67	13
Prr13	7,17E-71	1,428203	0,774	0,299	2,23E-66	13
Mgat5	7,34E-71	0,817887	0,318	0,048	2,28E-66	13
Ly6e	1,47E-70	1,222942	0,995	0,799	4,56E-66	13
Sptssa	2,56E-70	1,482234	0,77	0,286	7,94E-66	13
Il2ra	8,57E-70	0,907561	0,3	0,043	2,66E-65	13
Ero1l	2,84E-69	0,995465	0,387	0,071	8,81E-65	13
Lysmd2	1,48E-67	0,95133	0,387	0,073	4,6E-63	13
Eif4e3	3,96E-66	0,775255	0,313	0,049	1,23E-61	13
Plin3	8,92E-65	0,826511	0,267	0,038	2,77E-60	13
Cdkn1a	3,05E-64	1,180834	0,309	0,05	9,47E-60	13
Ahnak	1,13E-61	1,405095	0,783	0,305	3,51E-57	13
Selenop	1,9E-61	1,556459	0,733	0,3	5,91E-57	13
Plp2	1,37E-60	1,188093	0,406	0,089	4,25E-56	13
1810058I2	1,71E-60	1,362934	0,779	0,317	5,32E-56	13
Tmem64	6,52E-57	1,12403	0,585	0,178	2,02E-52	13
Wls	6,5E-56	0,747527	0,258	0,04	2,02E-51	13

Itm2b	7,57E-56	1,06357	0,986	0,829	2,35E-51	13
Gpx1	9,08E-56	1,597104	0,857	0,501	2,82E-51	13
Fosb	1,42E-55	1,661715	0,654	0,237	4,4E-51	13
Klf4	2,5E-54	1,492005	0,429	0,107	7,77E-50	13
Rnf144a	1,94E-53	0,788752	0,29	0,052	6,02E-49	13
Cd82	4,91E-53	1,176784	0,756	0,312	1,52E-48	13
Smpdl3a	3,2E-50	1,197908	0,631	0,231	9,95E-46	13
St6galnac:	7,34E-48	0,754902	0,253	0,044	2,28E-43	13
Zfp36l1	4,95E-47	1,48874	0,843	0,487	1,54E-42	13
Bcl2a1b	1,19E-46	1,258755	0,548	0,185	3,7E-42	13
Podnl1	3,07E-46	0,705735	0,304	0,061	9,53E-42	13
Clcn3	2,67E-44	0,923227	0,378	0,097	8,29E-40	13
Bcl2a1d	1,14E-43	0,883769	0,364	0,088	3,54E-39	13
St3gal6	2,58E-43	1,029171	0,475	0,151	8E-39	13
Lpcat3	2,35E-42	0,563775	0,253	0,047	7,3E-38	13
Ap3s1	3,67E-42	0,964104	0,512	0,168	1,14E-37	13
Rgcc	6,31E-42	1,826648	0,691	0,334	1,96E-37	13
Psap	7,37E-42	1,084371	0,802	0,447	2,29E-37	13
Trgv2	4,89E-40	0,93512	0,253	0,051	1,52E-35	13
Cd44	5,25E-40	0,927445	0,479	0,154	1,63E-35	13
Dennd4c	6,87E-40	0,723218	0,309	0,072	2,13E-35	13
Tspo	1E-39	1,009705	0,857	0,543	3,11E-35	13
Irak2	1,73E-38	0,738222	0,263	0,056	5,36E-34	13
Dusp1	7,37E-38	1,195898	0,871	0,549	2,29E-33	13
Gabbr1	1,68E-37	0,677289	0,263	0,056	5,21E-33	13
Emb	2,34E-37	1,029008	0,871	0,529	7,26E-33	13
Sept11	6,48E-37	0,798001	0,327	0,085	2,01E-32	13
CAAA011	9,7E-37	0,907495	0,447	0,146	3,01E-32	13
Fos	2,05E-36	1,591331	0,747	0,417	6,37E-32	13
Lsp1	2,58E-36	1,027475	0,88	0,621	8E-32	13
Atp2b1	5,13E-36	1,027586	0,571	0,228	1,59E-31	13
Rnh1	1,08E-35	0,771007	0,369	0,106	3,35E-31	13
Ifngr1	2,61E-35	1,312315	0,783	0,47	8,09E-31	13
Adgrg5	1,01E-34	0,650137	0,281	0,068	3,15E-30	13
Zfp36	1,56E-33	1,293487	0,793	0,465	4,83E-29	13
Itgb7	2,72E-33	0,934179	0,825	0,538	8,44E-29	13
Gclc	9,13E-33	0,685238	0,309	0,082	2,84E-28	13
Gpr183	9,46E-33	0,996409	0,645	0,29	2,94E-28	13
Tagln2	1,61E-32	1,01733	0,866	0,602	5,01E-28	13
Bhlhe40	3,2E-32	0,887538	0,516	0,193	9,94E-28	13
Sla	3,27E-32	0,868635	0,521	0,203	1,01E-27	13
Dap	5,07E-32	0,971835	0,484	0,186	1,58E-27	13
Vim	7,49E-32	1,134171	0,751	0,426	2,33E-27	13
Laptm5	1,03E-31	0,786661	0,949	0,8	3,2E-27	13
Prkx	1,06E-30	0,786321	0,373	0,118	3,29E-26	13
Timp2	2,18E-30	0,646474	0,276	0,071	6,78E-26	13
Prrt1	2,09E-29	0,626028	0,286	0,076	6,49E-25	13
Anxa2	9,65E-29	0,769721	0,332	0,099	3E-24	13
Cst3	3,99E-28	0,774151	0,493	0,199	1,24E-23	13
D16Ertd47	5,81E-28	0,805613	0,465	0,178	1,81E-23	13
Tmsb4x	6,1E-28	0,430913	1	0,994	1,89E-23	13
Serinc3	7,9E-28	0,827795	0,687	0,359	2,45E-23	13
Gadd45b	1,2E-27	1,024264	0,461	0,178	3,73E-23	13
Ppib	2,32E-27	0,766554	0,848	0,603	7,22E-23	13
Ptprcap	6,24E-27	0,762246	0,876	0,634	1,94E-22	13

Oaz1	7,88E-27	0,577639	0,986	0,931	2,45E-22	13
Thy1	9,28E-27	0,784333	0,912	0,751	2,88E-22	13
Prdx6	1,37E-26	0,858452	0,834	0,574	4,25E-22	13
Stk24	3,25E-26	0,828432	0,664	0,357	1,01E-21	13
Gm2a	4,09E-26	0,765959	0,442	0,175	1,27E-21	13
Rara	5,93E-26	0,674426	0,355	0,118	1,84E-21	13
Klf9	7,2E-26	0,683841	0,313	0,099	2,24E-21	13
Rexo2	8,53E-26	0,831486	0,571	0,279	2,65E-21	13
Pon2	9,15E-26	0,607109	0,323	0,102	2,84E-21	13
Faah	2,54E-25	0,682121	0,419	0,156	7,9E-21	13
Clic1	2,79E-25	0,695264	0,88	0,638	8,67E-21	13
Ppp1r11	4,6E-25	0,736187	0,461	0,189	1,43E-20	13
Coro2a	1,97E-24	0,626321	0,253	0,071	6,12E-20	13
Selplg	2,69E-24	0,706232	0,908	0,678	8,35E-20	13
Id2	6,35E-24	0,741284	0,719	0,388	1,97E-19	13
Arpc3	7,18E-24	0,658695	0,866	0,682	2,23E-19	13
Klrk1	1,14E-23	0,597061	0,327	0,105	3,54E-19	13
Pim1	2,2E-23	0,91106	0,756	0,48	6,83E-19	13
Junb	2,33E-23	0,683386	0,995	0,959	7,23E-19	13
Cd7	2,51E-23	0,850969	0,475	0,217	7,79E-19	13
Smco4	6,64E-23	0,588202	0,304	0,101	2,06E-18	13
Atp6v0e	7,57E-23	0,720144	0,622	0,327	2,35E-18	13
Srgn	8,37E-23	0,607333	0,963	0,855	2,6E-18	13
Elk3	9,72E-23	0,535436	0,263	0,079	3,02E-18	13
Lgals3bp	1,01E-22	0,69042	0,346	0,124	3,15E-18	13
Pdia4	1,19E-22	0,719253	0,327	0,116	3,7E-18	13
Tpst2	1,5E-22	0,816113	0,594	0,298	4,67E-18	13
Gpr132	2,64E-22	0,878052	0,714	0,436	8,19E-18	13
Trpv2	4,17E-22	0,544612	0,258	0,078	1,3E-17	13
Cish	4,85E-22	0,725831	0,318	0,11	1,51E-17	13
Camk4	1,26E-21	0,641646	0,415	0,166	3,92E-17	13
Myo1f	1,78E-21	0,512243	0,318	0,108	5,53E-17	13
Clint1	2,64E-21	0,729301	0,581	0,3	8,21E-17	13
Samsn1	4,7E-21	0,729062	0,382	0,154	1,46E-16	13
Tiparp	6,39E-21	0,914147	0,359	0,142	1,98E-16	13
Slc38a1	8,01E-21	0,728522	0,479	0,222	2,49E-16	13
Ssbp3	1,08E-20	0,523634	0,318	0,111	3,35E-16	13
Nrip1	1,15E-20	0,667741	0,447	0,192	3,56E-16	13
Sec11c	1,71E-20	0,7085	0,636	0,356	5,31E-16	13
Ncor1	2,73E-20	0,731231	0,737	0,464	8,48E-16	13
Uhrf2	2,86E-20	0,788559	0,332	0,127	8,89E-16	13
Ptpn7	3,61E-20	0,696019	0,392	0,164	1,12E-15	13
Comt	2,35E-19	0,688075	0,424	0,188	7,29E-15	13
Hsp90b1	3,15E-19	0,734645	0,668	0,404	9,79E-15	13
Gstp1	4,11E-19	0,69792	0,793	0,542	1,28E-14	13
Zap70	6,4E-19	0,689965	0,604	0,332	1,99E-14	13
Nr1d2	6,49E-19	0,505862	0,253	0,082	2,01E-14	13
Tmem154	6,78E-19	0,462569	0,272	0,092	2,1E-14	13
Csrnp1	8,33E-19	0,797226	0,613	0,353	2,59E-14	13
Mrps5	9,17E-19	0,724336	0,336	0,134	2,85E-14	13
Gnas	1,18E-18	0,567249	0,894	0,697	3,67E-14	13
Pfkp	1,52E-18	0,607639	0,364	0,146	4,71E-14	13
Zc3h12a	2,61E-18	0,742826	0,516	0,266	8,09E-14	13
Ucp2	2,76E-18	0,654702	0,857	0,659	8,57E-14	13
AI467606	3,2E-18	0,665334	0,272	0,098	9,95E-14	13

Gng2	3,67E-18	0,537087	0,318	0,122	1,14E-13	13
Siah2	7,13E-18	0,720859	0,406	0,187	2,21E-13	13
Nfkb1	7,28E-18	0,584896	0,479	0,231	2,26E-13	13
Odc1	7,46E-18	1,042511	0,567	0,331	2,32E-13	13
Crip1	8,32E-18	1,017704	0,889	0,746	2,58E-13	13
Mapkapk3	1,02E-17	0,486739	0,276	0,098	3,15E-13	13
Rnf166	1,43E-17	0,569402	0,516	0,262	4,44E-13	13
Mthfsl	1,66E-17	0,49006	0,272	0,096	5,16E-13	13
Cdc42ep3	1,94E-17	0,594448	0,29	0,107	6,03E-13	13
Ndufs5	2,08E-17	0,591742	0,47	0,228	6,47E-13	13
Arf5	3,44E-17	0,611483	0,797	0,622	1,07E-12	13
Sept9	4,83E-17	0,571056	0,636	0,362	1,5E-12	13
Eprs	4,98E-17	0,58246	0,41	0,187	1,54E-12	13
Ndfip1	5,21E-17	0,569811	0,783	0,528	1,62E-12	13
Sik1	7,17E-17	0,591902	0,396	0,181	2,23E-12	13
Serf2	8,65E-17	0,457981	0,963	0,906	2,69E-12	13
Ppia	1,39E-16	0,337484	0,995	0,987	4,32E-12	13
Mapk6	1,48E-16	0,531723	0,253	0,089	4,6E-12	13
Cmtm6	2,13E-16	0,633359	0,35	0,151	6,63E-12	13
Rinl	2,72E-16	0,653772	0,544	0,306	8,45E-12	13
Aldoa	4,22E-16	0,56127	0,765	0,575	1,31E-11	13
Plec	4,47E-16	0,823743	0,429	0,213	1,39E-11	13
Ptpra	4,93E-16	0,509651	0,332	0,137	1,53E-11	13
Itgal	6,33E-16	0,606647	0,599	0,338	1,97E-11	13
Fli1	8,43E-16	0,560862	0,41	0,193	2,62E-11	13
Ifrd1	1,09E-15	0,769912	0,682	0,446	3,39E-11	13
Dctn1	1,12E-15	0,616493	0,286	0,112	3,47E-11	13
Card19	1,25E-15	0,63896	0,318	0,136	3,9E-11	13
Tmbim4	1,69E-15	0,516502	0,304	0,124	5,23E-11	13
Ahcyl2	1,92E-15	0,527017	0,276	0,108	5,95E-11	13
Diaph1	1,92E-15	0,582945	0,47	0,241	5,95E-11	13
Fnbp1	3,35E-15	0,559583	0,493	0,261	1,04E-10	13
Hilpda	3,54E-15	0,673034	0,258	0,1	1,1E-10	13
Nr4a1	3,61E-15	0,843809	0,659	0,443	1,12E-10	13
Ppdpf	5,39E-15	0,601882	0,618	0,366	1,67E-10	13
Mif4gd	1,23E-14	0,494841	0,3	0,122	3,83E-10	13
Cd84	1,37E-14	0,492387	0,355	0,158	4,26E-10	13
Cox7c	1,9E-14	0,463741	0,899	0,797	5,91E-10	13
Emp3	1,91E-14	0,674577	0,733	0,512	5,92E-10	13
Prr7	3,14E-14	0,614524	0,263	0,105	9,74E-10	13
Cd3e	5,6E-14	0,426022	0,968	0,895	1,74E-09	13
Tbl1x	9,7E-14	0,456788	0,304	0,129	3,01E-09	13
Usmg5	1,22E-13	0,488865	0,825	0,657	3,78E-09	13
Actg1	1,24E-13	0,469763	0,963	0,928	3,87E-09	13
Smap1	1,34E-13	0,526256	0,535	0,304	4,17E-09	13
Fosl2	1,4E-13	0,724654	0,401	0,206	4,33E-09	13
S100a13	1,86E-13	0,529744	0,613	0,384	5,78E-09	13
Runx1	1,94E-13	0,461801	0,396	0,191	6,01E-09	13
Nfkbia	1,95E-13	0,576532	0,903	0,752	6,04E-09	13
Sh3bgrl3	1,98E-13	0,386033	0,959	0,864	6,15E-09	13
Wbp1	2,06E-13	0,404358	0,263	0,105	6,39E-09	13
Slc7a6os	2,82E-13	0,517341	0,267	0,109	8,76E-09	13
Pkp3	2,85E-13	0,484771	0,382	0,186	8,85E-09	13
Cwc25	3,11E-13	0,509099	0,253	0,102	9,67E-09	13
Tmem256	4,17E-13	0,469553	0,313	0,141	1,29E-08	13

Rgs1	4,72E-13	0,842427	0,507	0,308	1,47E-08	13
Timm13	6,92E-13	0,45926	0,613	0,37	2,15E-08	13
Rtn4	7,12E-13	0,533973	0,387	0,193	2,21E-08	13
Anxa5	7,2E-13	0,522648	0,341	0,158	2,24E-08	13
Cdc42	1,23E-12	0,461858	0,848	0,664	3,8E-08	13
Krtcap2	1,43E-12	0,51698	0,728	0,494	4,43E-08	13
Cd164	2,94E-12	0,543265	0,507	0,301	9,14E-08	13
Ppp1r9b	4,01E-12	0,380217	0,341	0,158	1,24E-07	13
Per1	4,01E-12	0,552831	0,571	0,357	1,25E-07	13
Rilpl2	4,02E-12	0,400045	0,258	0,108	1,25E-07	13
Kdm6b	4,18E-12	0,594382	0,521	0,305	1,3E-07	13
Cebpb	4,27E-12	0,840107	0,479	0,285	1,33E-07	13
Cox6c	4,43E-12	0,423368	0,862	0,725	1,38E-07	13
Pdia3	5,56E-12	0,603667	0,645	0,465	1,73E-07	13
Hspa5	7,54E-12	0,57156	0,853	0,708	2,34E-07	13
Cyba	7,89E-12	0,425707	0,853	0,653	2,45E-07	13
Maz	9,34E-12	0,4567	0,41	0,219	2,9E-07	13
Snx2	9,45E-12	0,406403	0,323	0,149	2,94E-07	13
Ppp3ca	1,18E-11	0,41472	0,332	0,158	3,67E-07	13
Minos1	1,2E-11	0,481986	0,521	0,315	3,72E-07	13
Il2rg	1,22E-11	0,441849	0,88	0,755	3,78E-07	13
Znrd1	1,39E-11	0,426672	0,378	0,188	4,31E-07	13
Ppp1ca	1,39E-11	0,419462	0,802	0,625	4,33E-07	13
Ebp	1,48E-11	0,395221	0,272	0,12	4,59E-07	13
BC004004	1,54E-11	0,458013	0,272	0,122	4,77E-07	13
Cdk2ap2	1,96E-11	0,549535	0,696	0,512	6,07E-07	13
Esyt1	2,29E-11	0,533565	0,516	0,316	7,1E-07	13
Ppp1r12a	2,33E-11	0,544872	0,618	0,406	7,22E-07	13
Lcp1	2,73E-11	0,427412	0,793	0,585	8,49E-07	13
Il2rb	2,89E-11	0,253246	0,65	0,376	8,99E-07	13
Gnb2	3,15E-11	0,471762	0,705	0,499	9,78E-07	13
Ndufa4	3,2E-11	0,407725	0,793	0,588	9,95E-07	13
Sem1	3,25E-11	0,446579	0,834	0,671	1,01E-06	13
Sit1	3,62E-11	0,51431	0,382	0,201	1,12E-06	13
Irs2	3,92E-11	0,648038	0,281	0,131	1,22E-06	13
Flna	4,35E-11	0,466613	0,618	0,394	1,35E-06	13
Ech1	4,7E-11	0,474405	0,327	0,159	1,46E-06	13
AC149090	5,08E-11	0,444872	0,488	0,283	1,58E-06	13
Prkar1a	5,74E-11	0,509706	0,664	0,472	1,78E-06	13
Tmem160	5,99E-11	0,498973	0,479	0,278	1,86E-06	13
Tax1bp1	7,55E-11	0,480405	0,604	0,383	2,34E-06	13
Cyb5a	8,07E-11	0,53126	0,47	0,276	2,5E-06	13
Jun	8,44E-11	1,056035	0,687	0,528	2,62E-06	13
Ldha	8,55E-11	0,392688	0,829	0,662	2,65E-06	13
Acat1	1,06E-10	0,398368	0,304	0,145	3,29E-06	13
Selenot	1,06E-10	0,568373	0,438	0,257	3,29E-06	13
Gnb1	1,56E-10	0,466478	0,475	0,278	4,84E-06	13
Uqcrb	1,61E-10	0,421266	0,631	0,415	5E-06	13
Ppp1r14b	1,78E-10	0,441311	0,373	0,2	5,52E-06	13
Reep5	1,8E-10	0,455786	0,544	0,333	5,6E-06	13
Cox6b1	1,85E-10	0,419321	0,742	0,579	5,76E-06	13
Rpn2	2,4E-10	0,437828	0,438	0,247	7,45E-06	13
Myl12b	2,64E-10	0,360006	0,899	0,829	8,2E-06	13
Nptn	2,8E-10	0,413443	0,35	0,184	8,71E-06	13
Surf4	2,83E-10	0,469789	0,355	0,19	8,78E-06	13

Vars	3E-10	0,435324	0,346	0,182	9,33E-06	13
Sub1	3,14E-10	0,445089	0,876	0,765	9,75E-06	13
Runx3	3,14E-10	0,457645	0,392	0,213	9,75E-06	13
Irf2bp2	3,35E-10	0,451157	0,346	0,181	1,04E-05	13
Gpr65	3,52E-10	0,391672	0,276	0,13	1,09E-05	13
Anxa6	3,65E-10	0,512496	0,636	0,425	1,13E-05	13
Timm23	4,34E-10	0,484584	0,493	0,303	1,35E-05	13
Tex264	4,85E-10	0,353719	0,276	0,128	1,5E-05	13
Rasgrp1	4,96E-10	0,461806	0,516	0,316	1,54E-05	13
Rab10os	5,02E-10	0,435592	0,406	0,221	1,56E-05	13
Rps6ka3	5,04E-10	0,443574	0,272	0,128	1,56E-05	13
Ralbp1	5,81E-10	0,508741	0,406	0,228	1,8E-05	13
Retreg2	6,96E-10	0,467759	0,295	0,148	2,16E-05	13
Btg2	8,35E-10	0,485399	0,641	0,451	2,59E-05	13
Cd3g	9,12E-10	0,398376	0,926	0,846	2,83E-05	13
Calm1	9,51E-10	0,377729	0,908	0,81	2,95E-05	13
Cfl1	9,64E-10	0,335366	0,954	0,923	2,99E-05	13
Cmtm7	1,12E-09	0,442101	0,396	0,217	3,48E-05	13
Tmem208	1,22E-09	0,390198	0,281	0,134	3,78E-05	13
Cox5a	1,29E-09	0,407996	0,682	0,494	4,02E-05	13
Itpr3	1,4E-09	0,489715	0,253	0,123	4,34E-05	13
Arhgdia	1,54E-09	0,391369	0,705	0,522	4,79E-05	13
Txndc17	1,67E-09	0,3766	0,392	0,217	5,18E-05	13
Mapk14	1,71E-09	0,441213	0,286	0,141	5,3E-05	13
Anxa11	1,72E-09	0,418291	0,346	0,183	5,33E-05	13
Abhd17a	1,74E-09	0,353294	0,359	0,189	5,41E-05	13
Ndufa12	1,75E-09	0,380679	0,355	0,188	5,42E-05	13
Rcsd1	2,39E-09	0,500039	0,373	0,216	7,41E-05	13
Capns1	2,5E-09	0,42222	0,516	0,32	7,77E-05	13
Galnt1	2,55E-09	0,413613	0,286	0,143	7,93E-05	13
Ndufa13	2,6E-09	0,345229	0,774	0,601	8,08E-05	13
Arf4	2,63E-09	0,444241	0,668	0,475	8,18E-05	13
Prdx2	2,83E-09	0,45859	0,419	0,248	8,78E-05	13
Lamtor1	3,01E-09	0,349619	0,35	0,185	9,35E-05	13
Dusp5	3,04E-09	0,527426	0,654	0,482	9,43E-05	13
Fmnl1	3,1E-09	0,440629	0,581	0,381	9,63E-05	13
Fth1	3,36E-09	0,383439	0,995	0,969	0,000104	13
Mrps12	3,66E-09	0,337696	0,267	0,128	0,000114	13
Ssna1	3,83E-09	0,406048	0,336	0,181	0,000119	13
Gch1	3,84E-09	0,43254	0,263	0,126	0,000119	13
Gna13	4,36E-09	0,531229	0,567	0,407	0,000135	13
Mtpn	4,7E-09	0,427422	0,3	0,154	0,000146	13
Cyth4	4,82E-09	0,429346	0,392	0,22	0,00015	13
Mtdh	5,53E-09	0,409298	0,502	0,314	0,000172	13
Pim3	5,78E-09	0,592645	0,332	0,182	0,00018	13
Map4	6,63E-09	0,318123	0,318	0,162	0,000206	13
Dock2	6,84E-09	0,442209	0,613	0,433	0,000213	13
Dnajc3	7,17E-09	0,445526	0,535	0,349	0,000223	13
Ccdc107	8,01E-09	0,329191	0,267	0,13	0,000249	13
Tmem14c	8,11E-09	0,417882	0,447	0,268	0,000252	13
Tgfb1	9,7E-09	0,376813	0,493	0,309	0,000301	13
Rps27l	1E-08	0,364215	0,346	0,187	0,000311	13
Elob	1,02E-08	0,333733	0,806	0,651	0,000317	13
Tmem50a	1,04E-08	0,400479	0,691	0,53	0,000323	13
Csnk2b	1,14E-08	0,486945	0,535	0,373	0,000353	13

Tnfaip3	1,17E-08	0,437932	0,871	0,751	0,000365	13
Ywhaz	1,22E-08	0,319957	0,903	0,787	0,00038	13
Selenof	1,47E-08	0,477085	0,59	0,42	0,000457	13
Vamp8	1,63E-08	0,39049	0,488	0,311	0,000507	13
Akirin1	1,82E-08	0,415327	0,276	0,142	0,000566	13
Chchd2	1,83E-08	0,326609	0,853	0,75	0,000567	13
Srpr	1,97E-08	0,403915	0,327	0,177	0,000612	13
Wdr83os	2,02E-08	0,339041	0,493	0,313	0,000626	13
Psd4	2,08E-08	0,381496	0,276	0,139	0,000645	13
Ost4	2,4E-08	0,417494	0,71	0,544	0,000746	13
Ubald1	2,48E-08	0,431967	0,295	0,155	0,000769	13
Tmco1	2,77E-08	0,37466	0,281	0,146	0,000859	13
Tmbim6	3,02E-08	0,357544	0,733	0,57	0,000939	13
Nfkbid	3,1E-08	0,414516	0,318	0,174	0,000961	13
March2	3,19E-08	0,367442	0,35	0,195	0,000991	13
Chmp2a	3,26E-08	0,405686	0,396	0,229	0,001012	13
Ubl5	3,34E-08	0,397542	0,737	0,592	0,001036	13
Psmb2	3,81E-08	0,37281	0,47	0,296	0,001182	13
Nr3c1	3,81E-08	0,416095	0,323	0,177	0,001185	13
Sugt1	4,03E-08	0,402523	0,373	0,224	0,001252	13
Laptm4a	4,61E-08	0,348312	0,341	0,19	0,001431	13
Ubc	5,13E-08	0,344333	0,954	0,844	0,001592	13
Mrpl36	5,37E-08	0,331314	0,281	0,144	0,001666	13
Rbl2	5,6E-08	0,408402	0,286	0,151	0,00174	13
Rab1b	5,77E-08	0,389486	0,401	0,245	0,001791	13
Ndufv3	5,95E-08	0,440935	0,594	0,447	0,001848	13
Arpc4	6,22E-08	0,411356	0,664	0,486	0,001932	13
Inpp5d	7,34E-08	0,391994	0,359	0,206	0,002279	13
Cdk11b	7,37E-08	0,604883	0,544	0,385	0,002288	13
Banf1	7,51E-08	0,395869	0,535	0,355	0,002332	13
Ctsa	8,34E-08	0,329883	0,373	0,215	0,002591	13
Dnajb11	8,5E-08	0,329865	0,3	0,16	0,00264	13
Glud1	9,08E-08	0,500374	0,456	0,297	0,00282	13
Nme2	9,19E-08	0,341402	0,871	0,771	0,002854	13
Mppe1	9,6E-08	0,35898	0,272	0,141	0,00298	13
Znrf1	1,07E-07	0,467049	0,429	0,274	0,003331	13
Txn1	1,08E-07	0,373317	0,516	0,348	0,003355	13
Fxyd5	1,09E-07	0,31435	0,94	0,891	0,003387	13
Abhd8	1,27E-07	0,365043	0,378	0,224	0,003958	13
Fam102a	1,29E-07	0,378642	0,442	0,279	0,004014	13
Gata3	1,32E-07	0,269757	0,336	0,182	0,004088	13
Csk	1,33E-07	0,368956	0,544	0,364	0,004145	13
Pld3	1,38E-07	0,345131	0,332	0,187	0,004294	13
Pkm	1,54E-07	0,367042	0,673	0,527	0,004772	13
Vdac1	1,75E-07	0,279408	0,332	0,184	0,005437	13
Idh3a	1,88E-07	0,364275	0,253	0,13	0,005851	13
5031425E	1,99E-07	0,302068	0,29	0,158	0,006189	13
Pfdn1	2,37E-07	0,326065	0,327	0,184	0,007351	13
Elmsan1	2,41E-07	0,446257	0,295	0,168	0,007496	13
Dynlrb1	2,49E-07	0,334826	0,461	0,293	0,007723	13
Pdia6	2,59E-07	0,348761	0,286	0,159	0,00803	13
Polr2j	2,59E-07	0,301168	0,272	0,145	0,008046	13
Ppp1cc	2,65E-07	0,458785	0,613	0,485	0,008222	13
Tab2	2,77E-07	0,32148	0,258	0,137	0,008592	13
Sar1a	2,99E-07	0,340966	0,336	0,195	0,009294	13

Ywhah	3,07E-07	0,319962	0,507	0,33	0,009519	13
Tor1aip1	3,6E-07	0,297358	0,304	0,168	0,011168	13
Nedd8	3,69E-07	0,328524	0,576	0,405	0,011448	13
Hcls1	3,75E-07	0,348831	0,456	0,291	0,011637	13
Ltb	4E-07	0,536494	0,802	0,739	0,01241	13
Calm3	4,21E-07	0,43674	0,456	0,306	0,013082	13
Cuta	4,33E-07	0,333292	0,456	0,291	0,013432	13
Psmb3	4,83E-07	0,323782	0,631	0,461	0,014994	13
BC031181	4,85E-07	0,323898	0,387	0,234	0,015069	13
Uqcr11	5E-07	0,414126	0,502	0,333	0,015536	13
Dad1	5,17E-07	0,358824	0,71	0,546	0,016065	13
Psmd8	5,39E-07	0,376063	0,475	0,315	0,016734	13
Tma7	5,67E-07	0,348004	0,742	0,602	0,017603	13
Manf	6,3E-07	0,449759	0,396	0,255	0,01957	13
Higd2a	6,91E-07	0,313251	0,452	0,294	0,02145	13
Psen2	6,98E-07	0,343087	0,253	0,134	0,021681	13
Arl2bp	7,44E-07	0,31511	0,461	0,298	0,023091	13
Fryl	7,71E-07	0,333825	0,364	0,224	0,023957	13
Spty2d1	9,07E-07	0,444022	0,304	0,18	0,028167	13
Neat1	9,73E-07	0,404451	0,309	0,182	0,030213	13
Xist	9,76E-07	0,329017	0,705	0,537	0,030304	13
Dctn3	9,95E-07	0,33401	0,387	0,24	0,0309	13
Ywhaq	1,04E-06	0,352077	0,465	0,312	0,03221	13
Tprgl	1,06E-06	0,407092	0,562	0,4	0,032802	13
D8Ertd738	1,07E-06	0,357072	0,737	0,566	0,033129	13
Cd47	1,09E-06	0,430848	0,7	0,591	0,033964	13
Rhog	1,14E-06	0,370977	0,512	0,353	0,035538	13
Ndufa7	1,18E-06	0,369672	0,673	0,543	0,036744	13
Emc10	1,23E-06	0,33126	0,47	0,311	0,038082	13
Pebp1	1,27E-06	0,29204	0,521	0,356	0,039492	13
Ier3ip1	1,36E-06	0,315491	0,488	0,334	0,042174	13
Tmem123	1,42E-06	0,370265	0,336	0,203	0,044229	13
Lypla2	1,43E-06	0,285914	0,433	0,277	0,044353	13
Mvb12a	1,44E-06	0,304544	0,267	0,146	0,044581	13
Kbtbd11	1,47E-06	0,452574	0,309	0,184	0,045506	13
Dtx1	1,5E-06	0,533428	0,309	0,184	0,046656	13
Tnfrsf9	1,3E-248	1,484123	0,401	0,021	4E-244	14
Irf8	6,5E-116	1,036947	0,29	0,025	2E-111	14
Srm	1,03E-83	1,382649	0,556	0,12	3,2E-79	14
Eif5a	2,18E-83	1,577101	0,99	0,764	6,76E-79	14
Ptma	1,32E-80	1,195687	0,995	0,956	4,11E-76	14
Hsp90ab1	2,31E-80	1,348245	1	0,938	7,19E-76	14
Ppa1	4,29E-75	0,967304	0,473	0,092	1,33E-70	14
Nhp2	1,56E-73	1,257172	0,686	0,195	4,84E-69	14
Ran	2,01E-67	1,457409	0,928	0,492	6,24E-63	14
Bcl2a1d	1,06E-65	1,213616	0,43	0,087	3,3E-61	14
Nop16	8,82E-65	0,724403	0,367	0,063	2,74E-60	14
Hspd1	4,28E-63	1,351404	0,671	0,217	1,33E-58	14
Mif	9,2E-62	1,675845	0,932	0,609	2,86E-57	14
Phgdh	3,47E-59	1,042587	0,531	0,137	1,08E-54	14
Ranbp1	8,38E-59	1,208402	0,768	0,306	2,6E-54	14
Mybbp1a	1,26E-58	0,972791	0,614	0,174	3,9E-54	14
Mettl1	2,18E-58	0,783343	0,401	0,081	6,77E-54	14
Galk1	4,79E-58	0,620653	0,304	0,049	1,49E-53	14
Pa2g4	9,38E-58	1,324324	0,758	0,303	2,91E-53	14

Fabp5	6,51E-56	0,635228	0,275	0,042	2,02E-51	14
C1qbp	6,75E-56	1,207014	0,696	0,257	2,1E-51	14
Nop2	4,27E-55	0,575998	0,3	0,049	1,33E-50	14
Ncl	8,19E-55	1,452592	0,923	0,624	2,54E-50	14
Apex1	4,43E-54	0,810876	0,444	0,101	1,38E-49	14
Ldha	1,07E-52	1,17463	0,957	0,66	3,31E-48	14
Bcl2a1b	1,47E-51	1,257702	0,589	0,185	4,57E-47	14
Nme1	4,9E-51	1,09074	0,739	0,292	1,52E-46	14
Nolc1	5,81E-48	1,002153	0,56	0,169	1,8E-43	14
Psat1	2,11E-47	0,538164	0,251	0,041	6,55E-43	14
Polr2l	2,4E-47	1,034144	0,739	0,291	7,45E-43	14
Timm8a1	5,49E-47	0,664897	0,372	0,082	1,7E-42	14
Cycs	6,8E-47	1,165296	0,807	0,42	2,11E-42	14
Atp5g1	1,14E-46	1,030435	0,884	0,499	3,53E-42	14
Utp20	1,46E-46	0,541875	0,29	0,053	4,54E-42	14
Hspa8	1,58E-46	1,001794	0,995	0,943	4,91E-42	14
St6galnac4	4,06E-46	0,624641	0,3	0,057	1,26E-41	14
Gadd45b	1,18E-45	1,06842	0,56	0,176	3,68E-41	14
Psme2	5,55E-45	1,214552	0,913	0,645	1,72E-40	14
Gar1	1,97E-44	0,827038	0,493	0,141	6,13E-40	14
Eif3b	4,33E-44	0,889237	0,614	0,207	1,34E-39	14
Fam162a	6,83E-44	0,742804	0,488	0,136	2,12E-39	14
Nop58	7,59E-44	0,991356	0,623	0,227	2,36E-39	14
Mrto4	7,86E-44	0,754183	0,469	0,127	2,44E-39	14
Lap3	7,99E-44	0,481605	0,275	0,05	2,48E-39	14
Rrp15	1,06E-42	0,542902	0,324	0,068	3,29E-38	14
Psma4	2,63E-42	0,818819	0,58	0,19	8,17E-38	14
Ipo5	1,21E-41	0,665517	0,372	0,089	3,75E-37	14
Nfkb1	1,52E-41	0,949429	0,623	0,229	4,71E-37	14
Ptpn6	1,75E-41	0,863706	0,599	0,208	5,44E-37	14
Timm10	6,54E-41	0,475657	0,251	0,046	2,03E-36	14
Prmt1	8,62E-41	0,839105	0,618	0,225	2,68E-36	14
Ruvbl1	4,91E-40	0,498861	0,314	0,067	1,53E-35	14
Cd82	6,82E-40	1,150064	0,715	0,312	2,12E-35	14
Shmt2	7,17E-40	0,70305	0,401	0,104	2,23E-35	14
Npm1	8,76E-40	0,987685	0,966	0,884	2,72E-35	14
Cct3	1,68E-39	0,949625	0,594	0,217	5,2E-35	14
Ybx1	3,25E-39	0,995371	0,928	0,701	1,01E-34	14
Dkc1	4,89E-39	0,648712	0,406	0,107	1,52E-34	14
Nutf2	7,19E-39	0,713192	0,522	0,165	2,23E-34	14
Mrps6	1,26E-38	0,606725	0,367	0,091	3,92E-34	14
Dctpp1	1,6E-38	0,633096	0,304	0,067	4,96E-34	14
Ppp1r14b	1,95E-38	0,828141	0,565	0,197	6,06E-34	14
Fbl	3,77E-38	0,982481	0,778	0,389	1,17E-33	14
Nsun2	6,2E-38	0,720453	0,478	0,145	1,93E-33	14
Tuba1b	6,22E-38	1,175988	0,792	0,46	1,93E-33	14
Ndufaf4	1,22E-37	0,629879	0,377	0,098	3,78E-33	14
Tomm40	1,35E-37	0,706945	0,415	0,116	4,18E-33	14
Pgam1	3,42E-37	0,831658	0,536	0,184	1,06E-32	14
Hspe1	4,26E-37	1,068294	0,908	0,603	1,32E-32	14
Tpi1	7,87E-37	0,836003	0,401	0,113	2,44E-32	14
Uqcc2	7,91E-37	0,813158	0,609	0,219	2,46E-32	14
Atp5g3	1,14E-36	0,94295	0,928	0,725	3,54E-32	14
Lyar	2,33E-36	0,630798	0,386	0,103	7,22E-32	14
Gapdh	2,96E-36	0,928271	0,976	0,905	9,19E-32	14

Hsph1	3,87E-36	0,595969	0,333	0,082	1,2E-31	14
Cct8	2,42E-35	0,867795	0,686	0,296	7,51E-31	14
Ddx21	3,58E-35	0,975727	0,686	0,299	1,11E-30	14
Set	5,44E-35	1,016032	0,836	0,51	1,69E-30	14
Noc2l	7,76E-35	0,719519	0,512	0,167	2,41E-30	14
Atad3a	8,28E-35	0,552525	0,275	0,061	2,57E-30	14
Sdhaf1	8,98E-35	0,684837	0,498	0,16	2,79E-30	14
Cdk4	1,12E-34	0,800913	0,614	0,244	3,48E-30	14
Banf1	1,22E-34	0,915159	0,729	0,353	3,8E-30	14
Psme3	2,07E-34	0,749601	0,469	0,153	6,43E-30	14
Nop10	3,35E-34	0,998518	0,816	0,502	1,04E-29	14
St13	4,37E-34	0,795367	0,585	0,219	1,36E-29	14
Anp32b	4,94E-34	0,890799	0,802	0,439	1,53E-29	14
Uqcrq	5,96E-34	0,839381	0,87	0,498	1,85E-29	14
Tm2d3	8,02E-34	0,460969	0,271	0,059	2,49E-29	14
Hsp90aa1	1,7E-33	0,99735	0,865	0,557	5,27E-29	14
Gm28285	2,14E-33	0,518375	0,304	0,073	6,64E-29	14
Ebna1bp2	2,8E-33	0,703985	0,464	0,147	8,7E-29	14
Gnl3	2,86E-33	0,822129	0,565	0,212	8,87E-29	14
Phb	4,49E-33	0,79353	0,541	0,207	1,39E-28	14
Nip7	6,23E-33	0,599286	0,406	0,119	1,94E-28	14
Rcc2	1,1E-32	0,720402	0,522	0,185	3,43E-28	14
Erh	1,19E-32	0,928528	0,729	0,356	3,7E-28	14
Rrp1b	1,43E-32	0,476861	0,271	0,061	4,43E-28	14
Ppia	2,45E-32	0,492932	0,995	0,987	7,6E-28	14
Psma7	2,81E-32	0,832991	0,768	0,414	8,74E-28	14
Aars	4,8E-32	0,442298	0,3	0,072	1,49E-27	14
Slc25a5	8,04E-32	0,866527	0,889	0,615	2,5E-27	14
Pdia6	1,14E-31	0,661628	0,478	0,156	3,53E-27	14
Polr2h	2,21E-31	0,468801	0,382	0,108	6,87E-27	14
Eif2s1	2,27E-31	0,663451	0,517	0,185	7,06E-27	14
Serbp1	2,34E-31	0,879928	0,899	0,681	7,27E-27	14
Snrpa1	2,97E-31	0,590785	0,454	0,144	9,21E-27	14
G3bp1	3,5E-31	0,838167	0,71	0,33	1,09E-26	14
Relb	1,03E-30	0,704403	0,415	0,13	3,18E-26	14
Slc7a5	1,61E-30	0,590416	0,29	0,073	5E-26	14
Eif4a1	2,35E-30	0,883915	0,899	0,629	7,29E-26	14
Fubp1	2,45E-30	0,683322	0,575	0,214	7,61E-26	14
Rps2	4,46E-30	0,494176	1	0,993	1,39E-25	14
Pfn1	4,52E-30	0,563338	0,995	0,968	1,4E-25	14
Xcl1	4,54E-30	1,634583	0,367	0,116	1,41E-25	14
Hspa9	7,37E-30	0,674154	0,594	0,233	2,29E-25	14
Eif1ax	1,21E-29	0,607902	0,483	0,169	3,76E-25	14
Uck2	3,17E-29	0,552347	0,343	0,098	9,83E-25	14
Tcp1	4,82E-29	0,84402	0,667	0,304	1,5E-24	14
Gart	7,63E-29	0,502397	0,295	0,077	2,37E-24	14
Eprs	8,05E-29	0,620797	0,507	0,186	2,5E-24	14
Foxp4	8,72E-29	0,526406	0,304	0,081	2,71E-24	14
Prpf19	2,16E-28	0,524248	0,444	0,146	6,7E-24	14
Cacybp	2,57E-28	0,648345	0,517	0,192	7,99E-24	14
Cox5a	2,73E-28	0,744919	0,826	0,492	8,48E-24	14
Nme2	4,38E-28	0,760647	0,957	0,77	1,36E-23	14
Sdf2l1	5,61E-28	0,41034	0,266	0,065	1,74E-23	14
Rps27l	5,8E-28	0,728286	0,502	0,185	1,8E-23	14
Eif3c	5,82E-28	0,70426	0,71	0,332	1,81E-23	14

Snrpf	1,06E-27	0,723352	0,928	0,61	3,3E-23	14
Gramd1b	1,19E-27	0,559497	0,324	0,092	3,68E-23	14
Stip1	1,34E-27	0,63912	0,406	0,135	4,16E-23	14
Elp5	1,54E-27	0,469619	0,261	0,065	4,77E-23	14
Mrpl12	5,26E-27	0,685572	0,483	0,181	1,63E-22	14
Eef1e1	5,32E-27	0,583366	0,382	0,12	1,65E-22	14
Chchd2	5,54E-27	0,66209	0,942	0,749	1,72E-22	14
Ddx39	5,66E-27	0,595118	0,498	0,184	1,76E-22	14
Rps17	6,86E-27	0,663346	0,971	0,836	2,13E-22	14
Eif5b	6,92E-27	0,701045	0,589	0,25	2,15E-22	14
Tufm	1,05E-26	0,51285	0,42	0,14	3,27E-22	14
Sms	1,23E-26	0,655626	0,551	0,22	3,82E-22	14
Nap1l1	1,24E-26	0,667986	0,643	0,293	3,85E-22	14
Kars	2,07E-26	0,53013	0,324	0,094	6,42E-22	14
Utp18	2,5E-26	0,403835	0,343	0,1	7,77E-22	14
Odc1	2,8E-26	0,779107	0,71	0,33	8,69E-22	14
Tmem147	3,55E-26	0,441854	0,372	0,116	1,1E-21	14
Rpn1	3,82E-26	0,595479	0,391	0,129	1,19E-21	14
Acot7	4,27E-26	0,522008	0,324	0,095	1,33E-21	14
Psme1	6,24E-26	0,676232	0,947	0,71	1,94E-21	14
Ftsj3	6,31E-26	0,522347	0,343	0,105	1,96E-21	14
Cd160	1,75E-25	0,488453	0,285	0,078	5,44E-21	14
Ppan	1,78E-25	0,601395	0,401	0,137	5,52E-21	14
Bud23	1,9E-25	0,38739	0,3	0,083	5,9E-21	14
Lsm7	2,15E-25	0,748482	0,618	0,285	6,68E-21	14
Ubl4a	2,58E-25	0,49177	0,333	0,101	8,01E-21	14
Wdr83os	3,63E-25	0,701924	0,647	0,311	1,13E-20	14
Eif2s2	4,18E-25	0,701021	0,807	0,48	1,3E-20	14
Pgk1	5,16E-25	0,78468	0,623	0,297	1,6E-20	14
Wdr18	5,82E-25	0,44602	0,309	0,09	1,81E-20	14
Bcl2l11	9,02E-25	0,667051	0,329	0,102	2,8E-20	14
Pomp	9,53E-25	0,734897	0,715	0,38	2,96E-20	14
Nifk	1,3E-24	0,505924	0,304	0,089	4,04E-20	14
Atp5e	1,66E-24	0,550456	0,976	0,884	5,14E-20	14
Eif4e	1,78E-24	0,599063	0,357	0,115	5,54E-20	14
Txn1	2,29E-24	0,786811	0,681	0,346	7,11E-20	14
Rpl7l1	2,33E-24	0,462808	0,309	0,091	7,24E-20	14
Tkt	2,48E-24	0,717763	0,594	0,283	7,7E-20	14
Mrps28	2,51E-24	0,446063	0,367	0,118	7,79E-20	14
Impdh2	2,82E-24	0,762957	0,614	0,301	8,75E-20	14
Psmd1	2,87E-24	0,477094	0,362	0,117	8,92E-20	14
Rsl1d1	3,92E-24	0,638664	0,575	0,246	1,22E-19	14
9130401M	3,97E-24	0,400505	0,295	0,084	1,23E-19	14
Hnrnpab	4,39E-24	0,77954	0,816	0,541	1,36E-19	14
Rel	4,69E-24	0,764412	0,469	0,181	1,46E-19	14
Yars	1,06E-23	0,302038	0,266	0,071	3,3E-19	14
Ndufab1	1,08E-23	0,617873	0,56	0,237	3,34E-19	14
Calr	1,16E-23	0,704164	0,758	0,414	3,61E-19	14
Wdr43	1,21E-23	0,58998	0,469	0,178	3,75E-19	14
Fkbp2	1,57E-23	0,389184	0,275	0,077	4,86E-19	14
Lcp1	1,9E-23	0,756254	0,87	0,584	5,9E-19	14
Tgfb1	2,72E-23	0,783074	0,643	0,307	8,45E-19	14
Mrpl42	3,26E-23	0,533857	0,42	0,152	1,01E-18	14
Cyc1	4,36E-23	0,567229	0,589	0,249	1,35E-18	14
Pabpc4	5,38E-23	0,445695	0,329	0,103	1,67E-18	14

Mcm6	9,24E-23	0,481984	0,333	0,105	2,87E-18	14
Nop56	1,17E-22	0,608645	0,575	0,255	3,63E-18	14
Ndufa12	1,55E-22	0,563851	0,469	0,186	4,8E-18	14
Strap	2,03E-22	0,619437	0,556	0,246	6,31E-18	14
Cox7b	2,21E-22	0,700754	0,812	0,519	6,86E-18	14
Stx6	2,4E-22	0,502887	0,275	0,081	7,44E-18	14
Syncrip	2,42E-22	0,571296	0,469	0,186	7,51E-18	14
Gars	2,49E-22	0,489492	0,401	0,142	7,73E-18	14
Pebp1	2,72E-22	0,706099	0,662	0,354	8,45E-18	14
Phb2	2,9E-22	0,658827	0,633	0,308	9,02E-18	14
Pkm	3,12E-22	0,74825	0,821	0,525	9,68E-18	14
Timm23	4,3E-22	0,628702	0,628	0,301	1,34E-17	14
Cct5	4,32E-22	0,686999	0,715	0,376	1,34E-17	14
Mdn1	5,61E-22	0,48307	0,372	0,13	1,74E-17	14
Gpr65	1,24E-21	0,471207	0,372	0,129	3,86E-17	14
Polr2f	1,35E-21	0,584554	0,454	0,178	4,2E-17	14
Npm3	1,35E-21	0,662376	0,671	0,354	4,21E-17	14
Ndufa5	1,41E-21	0,561263	0,56	0,245	4,37E-17	14
Rcl1	1,57E-21	0,425145	0,314	0,101	4,87E-17	14
Lrpprc	1,67E-21	0,315631	0,261	0,074	5,19E-17	14
Uchl3	1,72E-21	0,535978	0,435	0,162	5,36E-17	14
Pno1	2,39E-21	0,478801	0,314	0,102	7,42E-17	14
Pdap1	2,62E-21	0,593182	0,527	0,225	8,13E-17	14
Psmd7	2,63E-21	0,540863	0,454	0,178	8,15E-17	14
Farsa	2,71E-21	0,416779	0,275	0,082	8,43E-17	14
Mrpl3	3,28E-21	0,309569	0,261	0,074	1,02E-16	14
Tsr1	3,85E-21	0,4001	0,275	0,083	1,2E-16	14
Ndufc2	3,97E-21	0,518238	0,589	0,264	1,23E-16	14
Timm50	4,38E-21	0,397566	0,275	0,081	1,36E-16	14
Snrpa	4,54E-21	0,464868	0,382	0,136	1,41E-16	14
Abce1	4,64E-21	0,430422	0,329	0,11	1,44E-16	14
Psmb2	5,05E-21	0,604416	0,609	0,294	1,57E-16	14
Gm10131	5,55E-21	0,406261	0,261	0,077	1,72E-16	14
Ak2	6,28E-21	0,548716	0,483	0,199	1,95E-16	14
Nfkbid	6,45E-21	0,969551	0,425	0,172	2E-16	14
Ndufa4	6,96E-21	0,690655	0,87	0,587	2,16E-16	14
Cish	7,62E-21	0,500079	0,329	0,11	2,36E-16	14
Hsp90b1	8,05E-21	0,66793	0,725	0,403	2,5E-16	14
Hnrnpa2b'	8,43E-21	0,587911	0,937	0,762	2,62E-16	14
Zfp593	8,58E-21	0,47658	0,391	0,143	2,66E-16	14
Sept11	8,73E-21	0,339357	0,285	0,085	2,71E-16	14
Ssrp1	8,87E-21	0,519971	0,498	0,207	2,75E-16	14
Snrpd2	9,09E-21	0,713559	0,657	0,356	2,82E-16	14
Hnrnpa1	1,16E-20	0,630292	0,894	0,673	3,6E-16	14
Ccnd2	1,42E-20	0,852826	0,734	0,444	4,41E-16	14
Prdx1	1,43E-20	0,561632	0,802	0,463	4,45E-16	14
M6pr	1,46E-20	0,49331	0,57	0,243	4,52E-16	14
Psma5	1,58E-20	0,549903	0,478	0,197	4,9E-16	14
Vars	1,61E-20	0,545695	0,449	0,181	5,01E-16	14
Timm13	1,75E-20	0,597681	0,686	0,369	5,43E-16	14
Rrs1	2,36E-20	0,42217	0,28	0,088	7,33E-16	14
Rps19bp1	2,54E-20	0,36784	0,261	0,078	7,88E-16	14
Pum3	3,07E-20	0,348616	0,256	0,075	9,54E-16	14
Mak16	3,28E-20	0,449051	0,411	0,155	1,02E-15	14
Nars	3,33E-20	0,461327	0,44	0,17	1,03E-15	14

Atp5b	5E-20	0,64876	0,855	0,627	1,55E-15	14
Cdv3	7,46E-20	0,477809	0,396	0,149	2,32E-15	14
Cct2	1,09E-19	0,633023	0,739	0,427	3,39E-15	14
Cyba	1,34E-19	0,693747	0,889	0,653	4,16E-15	14
Cdc37	1,37E-19	0,644763	0,71	0,403	4,26E-15	14
Tomm5	1,4E-19	0,628013	0,551	0,27	4,35E-15	14
Fkbp4	1,56E-19	0,519002	0,498	0,209	4,85E-15	14
Get4	1,96E-19	0,360514	0,391	0,142	6,08E-15	14
Gspt1	2,23E-19	0,421841	0,396	0,149	6,91E-15	14
Denr	2,82E-19	0,525482	0,44	0,183	8,77E-15	14
Eif3d	3,11E-19	0,51151	0,541	0,243	9,66E-15	14
Eif4g1	3,34E-19	0,573286	0,536	0,249	1,04E-14	14
Ppid	3,34E-19	0,467758	0,314	0,108	1,04E-14	14
Mrpl21	3,49E-19	0,481543	0,473	0,195	1,08E-14	14
Psmg4	3,65E-19	0,444369	0,411	0,158	1,13E-14	14
Pin1	3,71E-19	0,413605	0,357	0,128	1,15E-14	14
Nudc	4,38E-19	0,566445	0,522	0,239	1,36E-14	14
Mphosph1	8,32E-19	0,349997	0,348	0,123	2,58E-14	14
Atp2a2	8,55E-19	0,303595	0,256	0,078	2,65E-14	14
Rilpl2	8,8E-19	0,522958	0,304	0,107	2,73E-14	14
Myc	9,09E-19	0,641845	0,3	0,105	2,82E-14	14
Ddx27	9,66E-19	0,441535	0,396	0,151	3E-14	14
Chchd1	1E-18	0,461831	0,459	0,189	3,12E-14	14
Snrpb	1,03E-18	0,569431	0,787	0,505	3,19E-14	14
Mrpl15	1,75E-18	0,427127	0,406	0,156	5,44E-14	14
Uqcr11	1,77E-18	0,550704	0,633	0,331	5,5E-14	14
Slc25a39	1,97E-18	0,321975	0,372	0,136	6,13E-14	14
Ssr2	2,18E-18	0,471143	0,435	0,179	6,76E-14	14
Tomm70a	2,32E-18	0,461615	0,411	0,162	7,21E-14	14
Rangap1	2,57E-18	0,372552	0,314	0,107	7,99E-14	14
Sssca1	2,89E-18	0,431937	0,309	0,107	8,99E-14	14
Lsm6	3,55E-18	0,452244	0,551	0,248	1,1E-13	14
Ccnh	3,55E-18	0,391608	0,261	0,083	1,1E-13	14
Mthfd2	3,81E-18	0,37635	0,275	0,089	1,18E-13	14
Rbbp7	3,89E-18	0,573847	0,527	0,24	1,21E-13	14
Bcap29	3,94E-18	0,358339	0,251	0,079	1,22E-13	14
Trim28	4,17E-18	0,447658	0,522	0,225	1,29E-13	14
Psmb5	5,07E-18	0,568335	0,599	0,299	1,57E-13	14
Gnl2	5,25E-18	0,398744	0,266	0,086	1,63E-13	14
Eef1g	5,38E-18	0,578645	0,874	0,696	1,67E-13	14
Tmem256	5,74E-18	0,472865	0,362	0,14	1,78E-13	14
Ly6a	5,82E-18	1,174866	0,507	0,259	1,81E-13	14
Alyref	6,35E-18	0,491292	0,502	0,221	1,97E-13	14
Tbrg4	6,52E-18	0,472964	0,319	0,116	2,03E-13	14
Cnih4	7,83E-18	0,318701	0,261	0,082	2,43E-13	14
Psma2	8,25E-18	0,565359	0,787	0,478	2,56E-13	14
Glrx3	8,52E-18	0,538111	0,498	0,229	2,64E-13	14
Khsrp	8,84E-18	0,380246	0,29	0,098	2,75E-13	14
Pbdc1	9,9E-18	0,302056	0,3	0,101	3,07E-13	14
Fasn	1,09E-17	0,390798	0,256	0,082	3,39E-13	14
Higd1a	1,1E-17	0,543501	0,546	0,26	3,43E-13	14
Sfxn1	1,25E-17	0,377698	0,329	0,119	3,87E-13	14
Pcbp1	1,35E-17	0,583969	0,797	0,526	4,2E-13	14
Trp53	1,39E-17	0,461275	0,498	0,225	4,32E-13	14
Nfkbib	1,75E-17	0,466555	0,44	0,183	5,45E-13	14

Dtymk	1,76E-17	0,313153	0,295	0,099	5,47E-13	14
Mrps7	1,76E-17	0,339525	0,329	0,117	5,48E-13	14
Lsm2	1,94E-17	0,42749	0,333	0,125	6,03E-13	14
Srsf2	1,98E-17	0,602589	0,845	0,573	6,15E-13	14
Dnajb11	2,03E-17	0,417351	0,401	0,159	6,31E-13	14
Ndufa11	2,74E-17	0,548843	0,585	0,297	8,5E-13	14
Mrpl17	3,37E-17	0,323173	0,309	0,109	1,05E-12	14
Psma6	3,73E-17	0,421928	0,507	0,224	1,16E-12	14
Rnf126	4,62E-17	0,307779	0,338	0,124	1,43E-12	14
Psmc4	4,72E-17	0,416652	0,454	0,193	1,47E-12	14
Rgs3	5,12E-17	0,408899	0,338	0,126	1,59E-12	14
Hnrnpu	5,14E-17	0,589684	0,802	0,556	1,6E-12	14
Aldoa	5,43E-17	0,684261	0,826	0,574	1,69E-12	14
Metap2	5,55E-17	0,489294	0,58	0,276	1,72E-12	14
Mea1	5,62E-17	0,41321	0,425	0,175	1,75E-12	14
Polr2e	5,71E-17	0,564748	0,464	0,215	1,77E-12	14
Eif6	6,03E-17	0,422263	0,454	0,193	1,87E-12	14
2210016F	6,22E-17	0,295056	0,309	0,108	1,93E-12	14
Txnl4a	7,38E-17	0,311139	0,29	0,1	2,29E-12	14
Abcf1	7,43E-17	0,455766	0,483	0,213	2,31E-12	14
Bax	7,48E-17	0,554381	0,633	0,329	2,32E-12	14
Usmg5	7,58E-17	0,509404	0,903	0,656	2,35E-12	14
Siva1	8,34E-17	0,328986	0,266	0,088	2,59E-12	14
Rpl41	8,74E-17	0,274664	1	0,996	2,71E-12	14
Adss	1,04E-16	0,458777	0,435	0,187	3,24E-12	14
Ndufb6	1,09E-16	0,502624	0,551	0,268	3,39E-12	14
Atp5d	1,13E-16	0,542501	0,87	0,652	3,5E-12	14
Pes1	1,25E-16	0,290175	0,304	0,107	3,88E-12	14
Canx	1,28E-16	0,49932	0,507	0,247	3,96E-12	14
Ndufb4	1,55E-16	0,484724	0,691	0,374	4,8E-12	14
Txnrd1	1,62E-16	0,346005	0,271	0,092	5,03E-12	14
Dad1	1,79E-16	0,532732	0,836	0,545	5,55E-12	14
Eftud2	2,14E-16	0,3922	0,333	0,128	6,66E-12	14
Glrx5	2,2E-16	0,373463	0,464	0,201	6,83E-12	14
Lrmp	2,24E-16	0,303276	0,261	0,086	6,95E-12	14
Mrpl36	2,28E-16	0,364804	0,367	0,143	7,07E-12	14
Caprin1	2,7E-16	0,394916	0,556	0,254	8,39E-12	14
Atp5j2	3,19E-16	0,486181	0,884	0,656	9,9E-12	14
Ddx10	3,37E-16	0,37024	0,3	0,11	1,05E-11	14
Mrps36	3,38E-16	0,34634	0,396	0,158	1,05E-11	14
Ahsa1	3,45E-16	0,420268	0,391	0,162	1,07E-11	14
Eif1a	3,84E-16	0,330351	0,271	0,093	1,19E-11	14
Gm26825	4,22E-16	0,622683	0,348	0,14	1,31E-11	14
Ptges3	4,29E-16	0,560251	0,599	0,315	1,33E-11	14
Adsl	4,34E-16	0,278525	0,329	0,121	1,35E-11	14
Naa10	4,62E-16	0,363087	0,454	0,193	1,44E-11	14
Smarcc1	4,98E-16	0,29048	0,251	0,082	1,55E-11	14
Gtpbp4	5,57E-16	0,402045	0,425	0,179	1,73E-11	14
Hdgf	6,24E-16	0,513788	0,541	0,264	1,94E-11	14
1810022K	6,38E-16	0,56111	0,657	0,364	1,98E-11	14
Snrpd3	6,54E-16	0,5788	0,676	0,405	2,03E-11	14
Psmd11	6,66E-16	0,418949	0,483	0,215	2,07E-11	14
Hnrnpc	6,88E-16	0,424543	0,657	0,338	2,14E-11	14
Kdm2b	6,93E-16	0,629615	0,353	0,144	2,15E-11	14
Dnajc19	8,03E-16	0,442759	0,502	0,233	2,49E-11	14

Psmb7	9,26E-16	0,369972	0,343	0,133	2,88E-11	14
Fdx1l	9,37E-16	0,329982	0,295	0,107	2,91E-11	14
Hars	1,03E-15	0,300616	0,285	0,101	3,18E-11	14
Suclg1	1,04E-15	0,314055	0,386	0,155	3,24E-11	14
Clns1a	1,19E-15	0,352482	0,382	0,153	3,69E-11	14
Rbm8a	1,22E-15	0,47685	0,507	0,237	3,78E-11	14
Rwdd1	1,31E-15	0,417899	0,589	0,292	4,06E-11	14
Snrpd1	1,42E-15	0,583781	0,638	0,374	4,42E-11	14
Lman2	1,47E-15	0,408225	0,459	0,206	4,55E-11	14
Bsg	1,52E-15	0,422529	0,589	0,291	4,72E-11	14
Dnajc2	1,58E-15	0,41312	0,449	0,197	4,89E-11	14
Txnl1	1,67E-15	0,424603	0,464	0,21	5,2E-11	14
Naa50	1,76E-15	0,334278	0,329	0,125	5,46E-11	14
Ppp1r11	1,78E-15	0,411391	0,44	0,189	5,53E-11	14
Bccip	1,93E-15	0,368694	0,406	0,171	5,98E-11	14
Snrpe	1,95E-15	0,525925	0,865	0,636	6,04E-11	14
Hnrnpa3	2,02E-15	0,537838	0,928	0,745	6,28E-11	14
Ddost	2,06E-15	0,520316	0,512	0,244	6,4E-11	14
P4hb	2,24E-15	0,413798	0,56	0,269	6,97E-11	14
Ndfip1	2,25E-15	0,67205	0,792	0,528	6,97E-11	14
Polr2k	2,31E-15	0,418743	0,449	0,197	7,17E-11	14
Cnbp	2,78E-15	0,506554	0,932	0,788	8,62E-11	14
Prpf40a	2,81E-15	0,40443	0,459	0,202	8,73E-11	14
Eif3a	2,87E-15	0,492002	0,647	0,354	8,91E-11	14
Mrpl35	3,38E-15	0,346272	0,314	0,118	1,05E-10	14
Isg15	3,48E-15	0,623366	0,314	0,124	1,08E-10	14
Mtap	3,55E-15	0,347402	0,275	0,099	1,1E-10	14
Mrpl23	3,8E-15	0,486278	0,657	0,351	1,18E-10	14
Elob	4,17E-15	0,478647	0,879	0,651	1,29E-10	14
Sarnp	4,98E-15	0,409497	0,527	0,253	1,54E-10	14
Minos1	4,98E-15	0,450436	0,604	0,314	1,55E-10	14
Acsl5	5,17E-15	0,385262	0,309	0,119	1,61E-10	14
Mapkapk3	5,34E-15	0,426113	0,271	0,098	1,66E-10	14
Ndufb8	5,76E-15	0,464041	0,58	0,302	1,79E-10	14
Atp5j	8,53E-15	0,507027	0,816	0,596	2,65E-10	14
Ptprcap	9,08E-15	0,505861	0,889	0,634	2,82E-10	14
Bhlhe40	1E-14	0,343104	0,435	0,195	3,12E-10	14
Ndufb2	1,26E-14	0,392617	0,512	0,242	3,91E-10	14
Hnrnpdl	1,73E-14	0,54219	0,831	0,566	5,36E-10	14
Txn2	1,83E-14	0,58367	0,575	0,311	5,68E-10	14
Hprt	1,99E-14	0,459957	0,502	0,242	6,17E-10	14
Rbmxl1	2,02E-14	0,350489	0,271	0,1	6,28E-10	14
Sec13	2,04E-14	0,315041	0,377	0,156	6,32E-10	14
Pim3	2,31E-14	0,480484	0,401	0,181	7,16E-10	14
Kpnb1	2,4E-14	0,395182	0,44	0,199	7,45E-10	14
Timm17a	2,44E-14	0,354401	0,377	0,16	7,59E-10	14
Slc35a4	2,61E-14	0,281106	0,266	0,095	8,12E-10	14
Gadd45gi[	2,63E-14	0,33988	0,411	0,179	8,17E-10	14
Syngr2	2,83E-14	0,371527	0,329	0,132	8,79E-10	14
Sumo2	2,94E-14	0,448243	0,874	0,614	9,12E-10	14
Stat1	3,6E-14	0,791783	0,594	0,336	1,12E-09	14
Grpel1	3,97E-14	0,41543	0,464	0,209	1,23E-09	14
Ak6	4,62E-14	0,320251	0,324	0,128	1,43E-09	14
Ola1	4,77E-14	0,3361	0,391	0,166	1,48E-09	14
Psmc1	4,81E-14	0,338636	0,367	0,154	1,49E-09	14

Eif3g	5,05E-14	0,453565	0,512	0,256	1,57E-09	14
Cebpz	5,35E-14	0,451971	0,512	0,258	1,66E-09	14
Ccdc124	5,48E-14	0,383255	0,435	0,194	1,7E-09	14
Ndufb7	5,55E-14	0,420862	0,638	0,362	1,72E-09	14
Dynll2	6,05E-14	0,343662	0,319	0,127	1,88E-09	14
Phf5a	6,15E-14	0,32394	0,43	0,189	1,91E-09	14
Slc1a5	6,21E-14	0,462655	0,507	0,247	1,93E-09	14
Smu1	6,31E-14	0,337501	0,348	0,144	1,96E-09	14
Mrpl54	7,17E-14	0,369232	0,483	0,223	2,23E-09	14
Vdac2	8,45E-14	0,465827	0,633	0,351	2,62E-09	14
Bst2	8,56E-14	0,609738	0,512	0,263	2,66E-09	14
Mdh2	8,7E-14	0,469131	0,676	0,411	2,7E-09	14
Psmd2	9,52E-14	0,336651	0,348	0,145	2,96E-09	14
Edf1	9,53E-14	0,498881	0,797	0,51	2,96E-09	14
Psma3	9,68E-14	0,471639	0,715	0,442	3,01E-09	14
Gps1	1,09E-13	0,333109	0,251	0,092	3,39E-09	14
Cops6	1,12E-13	0,393209	0,473	0,223	3,48E-09	14
Emc6	1,2E-13	0,520108	0,493	0,252	3,72E-09	14
Psmd14	1,32E-13	0,308913	0,401	0,175	4,1E-09	14
Uqcr10	1,46E-13	0,532332	0,715	0,458	4,54E-09	14
2010107E	1,47E-13	0,435269	0,874	0,679	4,57E-09	14
Ywhag	1,76E-13	0,345587	0,314	0,127	5,45E-09	14
Snx5	2,49E-13	0,384026	0,599	0,305	7,72E-09	14
Rce1	2,81E-13	0,281005	0,271	0,102	8,73E-09	14
Hypk	3E-13	0,407604	0,536	0,269	9,32E-09	14
Mrpl20	3,04E-13	0,330188	0,454	0,209	9,43E-09	14
Nfkb2	3,36E-13	0,365097	0,3	0,12	1,04E-08	14
E2f4	3,38E-13	0,290582	0,464	0,21	1,05E-08	14
Dut	3,72E-13	0,345755	0,314	0,129	1,16E-08	14
Nadk	3,73E-13	0,31636	0,28	0,108	1,16E-08	14
Stoml2	3,81E-13	0,337178	0,285	0,112	1,18E-08	14
Cbx1	3,85E-13	0,302083	0,357	0,151	1,2E-08	14
Ctdnep1	3,88E-13	0,286995	0,285	0,11	1,21E-08	14
Isyna1	3,99E-13	0,340314	0,314	0,129	1,24E-08	14
Paics	4,06E-13	0,358104	0,382	0,168	1,26E-08	14
Eif3l	4,43E-13	0,401768	0,464	0,228	1,38E-08	14
Psmc5	4,82E-13	0,365279	0,435	0,204	1,5E-08	14
Bzw2	5,07E-13	0,397105	0,502	0,248	1,57E-08	14
Icam1	5,12E-13	0,699216	0,314	0,136	1,59E-08	14
Larp1	5,19E-13	0,408121	0,42	0,199	1,61E-08	14
Cct7	5,33E-13	0,522381	0,609	0,36	1,66E-08	14
Hnrnpk	5,84E-13	0,440197	0,845	0,6	1,81E-08	14
Erap1	6,17E-13	0,309158	0,3	0,121	1,92E-08	14
Pdia4	7,02E-13	0,356534	0,29	0,117	2,18E-08	14
Rnps1	8,05E-13	0,373691	0,512	0,259	2,5E-08	14
Pdia3	8,2E-13	0,570821	0,71	0,464	2,55E-08	14
Spcs1	9,65E-13	0,432422	0,643	0,355	3E-08	14
Slc38a1	1,1E-12	0,428594	0,454	0,223	3,41E-08	14
Ilf3	1,19E-12	0,281515	0,251	0,094	3,69E-08	14
Rsl24d1	1,38E-12	0,383905	0,372	0,168	4,29E-08	14
Atp5o.1	1,43E-12	0,449596	0,686	0,413	4,45E-08	14
Psmb3	1,64E-12	0,465695	0,729	0,46	5,08E-08	14
Bola3	1,98E-12	0,366731	0,425	0,2	6,16E-08	14
Anp32e	2,01E-12	0,417096	0,43	0,204	6,24E-08	14
Vdac3	2,16E-12	0,28184	0,382	0,168	6,71E-08	14

Aprt	2,19E-12	0,387964	0,58	0,311	6,82E-08	14
Uqcrb	2,21E-12	0,489129	0,671	0,415	6,88E-08	14
Drg1	2,27E-12	0,328427	0,28	0,114	7,05E-08	14
Ndufv2	2,31E-12	0,415032	0,478	0,237	7,17E-08	14
Gtf2h5	2,43E-12	0,334784	0,449	0,209	7,54E-08	14
Dcun1d5	2,47E-12	0,431152	0,541	0,289	7,66E-08	14
Sf3a2	3,08E-12	0,335261	0,43	0,201	9,57E-08	14
Ube2g1	3,11E-12	0,305128	0,251	0,097	9,66E-08	14
U2af1	3,81E-12	0,452407	0,614	0,347	1,18E-07	14
Ddx41	3,95E-12	0,266405	0,29	0,116	1,23E-07	14
Imp4	4,36E-12	0,281484	0,362	0,161	1,35E-07	14
Rad23b	4,68E-12	0,309493	0,425	0,198	1,45E-07	14
Bola2	4,81E-12	0,450513	0,758	0,509	1,49E-07	14
Rps25	4,85E-12	0,367797	0,961	0,881	1,51E-07	14
Dusp2	4,94E-12	0,630458	0,647	0,394	1,53E-07	14
Rabggtb	5,56E-12	0,326042	0,319	0,136	1,73E-07	14
Psmb10	6,02E-12	0,520344	0,589	0,35	1,87E-07	14
Psmb6	6,11E-12	0,432026	0,594	0,34	1,9E-07	14
Llph	6,12E-12	0,459044	0,638	0,386	1,9E-07	14
Ssr4	6,44E-12	0,409869	0,681	0,431	2E-07	14
Psma1	6,77E-12	0,341932	0,411	0,194	2,1E-07	14
Ppie	6,84E-12	0,299198	0,275	0,111	2,12E-07	14
Vcp	6,97E-12	0,421325	0,56	0,306	2,17E-07	14
Ufm1	7,07E-12	0,292809	0,338	0,147	2,2E-07	14
Atp5a1	7,58E-12	0,439949	0,845	0,628	2,35E-07	14
Mbd3	8,83E-12	0,348182	0,362	0,167	2,74E-07	14
Las1l	9,14E-12	0,382316	0,266	0,104	2,84E-07	14
Isy1	1,02E-11	0,324856	0,367	0,165	3,18E-07	14
Copz1	1,04E-11	0,290204	0,372	0,167	3,23E-07	14
Hspbp1	1,05E-11	0,339106	0,309	0,135	3,26E-07	14
Hmgn1	1,22E-11	0,524104	0,58	0,35	3,78E-07	14
Hdac2	1,28E-11	0,280974	0,309	0,131	3,96E-07	14
Ddb1	1,48E-11	0,303896	0,357	0,161	4,58E-07	14
Arl1	1,51E-11	0,307448	0,295	0,125	4,69E-07	14
Sec61g	1,57E-11	0,396792	0,894	0,713	4,87E-07	14
Gm47283	1,57E-11	0,531596	0,681	0,42	4,87E-07	14
Chd4	1,73E-11	0,429385	0,604	0,341	5,38E-07	14
Tcerg1	1,76E-11	0,323532	0,324	0,143	5,48E-07	14
Tcof1	1,86E-11	0,41172	0,502	0,265	5,78E-07	14
Sars	1,95E-11	0,345321	0,502	0,254	6,06E-07	14
Btf3	2,17E-11	0,345419	0,976	0,887	6,74E-07	14
Cfl1	2,31E-11	0,329105	0,976	0,923	7,16E-07	14
Nsfl1c	2,4E-11	0,28065	0,357	0,16	7,45E-07	14
Eif2s3x	2,44E-11	0,302262	0,435	0,21	7,58E-07	14
B4galnt1	2,44E-11	0,554055	0,812	0,594	7,59E-07	14
Rpl31	2,62E-11	0,371623	0,957	0,86	8,15E-07	14
Zmiz2	3,31E-11	0,293197	0,295	0,127	1,03E-06	14
Ifi47	3,44E-11	0,47208	0,488	0,257	1,07E-06	14
Nop14	3,69E-11	0,261433	0,251	0,1	1,15E-06	14
Snx3	3,75E-11	0,365984	0,604	0,345	1,17E-06	14
Psmc3	3,8E-11	0,33142	0,483	0,252	1,18E-06	14
Orai1	4,03E-11	0,335795	0,353	0,161	1,25E-06	14
Sem1	4,08E-11	0,401339	0,874	0,671	1,27E-06	14
Hnrnpd	4,08E-11	0,337092	0,556	0,302	1,27E-06	14
Ssb	4,19E-11	0,352976	0,556	0,303	1,3E-06	14

Cox6c	4,3E-11	0,398394	0,894	0,724	1,34E-06	14
Rrp1	4,43E-11	0,356098	0,551	0,302	1,38E-06	14
Atp5k	4,85E-11	0,41653	0,763	0,491	1,5E-06	14
Hspa5	5,55E-11	0,480386	0,889	0,708	1,72E-06	14
Pdcd5	5,77E-11	0,401006	0,556	0,318	1,79E-06	14
Izumo1r	5,85E-11	0,500246	0,271	0,116	1,82E-06	14
Smarca4	5,85E-11	0,255194	0,382	0,176	1,82E-06	14
Tubb5	6,61E-11	0,500024	0,831	0,635	2,05E-06	14
Mat2a	6,73E-11	0,387754	0,551	0,301	2,09E-06	14
Stat5a	7,34E-11	0,279788	0,271	0,113	2,28E-06	14
Rps12	7,48E-11	0,284207	0,995	0,962	2,32E-06	14
Csnk2b	8,3E-11	0,422842	0,614	0,372	2,58E-06	14
Rpl14	8,68E-11	0,256405	0,995	0,973	2,7E-06	14
Ipo7	8,97E-11	0,325836	0,261	0,11	2,78E-06	14
Sla	1,04E-10	0,420194	0,406	0,204	3,23E-06	14
Tmem11	1,05E-10	0,254234	0,285	0,121	3,26E-06	14
Uba1	1,14E-10	0,282608	0,498	0,252	3,53E-06	14
Eif3j1	1,16E-10	0,363366	0,488	0,256	3,59E-06	14
Zcrb1	1,17E-10	0,254172	0,449	0,222	3,63E-06	14
Etf1	1,21E-10	0,364416	0,459	0,237	3,76E-06	14
Lsm12	1,22E-10	0,303582	0,401	0,197	3,78E-06	14
Eif1ad	1,25E-10	0,307163	0,271	0,114	3,89E-06	14
Abhd11	1,3E-10	0,292997	0,251	0,104	4,04E-06	14
Brix1	1,31E-10	0,292062	0,324	0,145	4,08E-06	14
Cfdp1	1,36E-10	0,297973	0,372	0,176	4,22E-06	14
Mrps17	1,41E-10	0,266135	0,275	0,116	4,36E-06	14
Ndufs6	1,44E-10	0,298889	0,425	0,207	4,48E-06	14
Lsm3	1,53E-10	0,286112	0,42	0,202	4,75E-06	14
Ptp4a3	1,53E-10	0,308429	0,42	0,208	4,76E-06	14
Top1	1,55E-10	0,284026	0,536	0,284	4,83E-06	14
Ivns1abp	1,68E-10	0,305001	0,353	0,167	5,23E-06	14
Chordc1	1,76E-10	0,344305	0,3	0,132	5,47E-06	14
Cox7c	1,98E-10	0,372149	0,928	0,796	6,15E-06	14
Jak2	2,09E-10	0,475732	0,304	0,143	6,49E-06	14
Mrps33	2,49E-10	0,370508	0,469	0,245	7,72E-06	14
Atic	2,59E-10	0,311535	0,275	0,121	8,05E-06	14
Chmp2a	2,79E-10	0,311494	0,449	0,228	8,66E-06	14
Taf1d	2,81E-10	0,415366	0,686	0,431	8,72E-06	14
Utp14a	2,89E-10	0,260539	0,261	0,111	8,97E-06	14
Ube2n	3,09E-10	0,268809	0,478	0,244	9,61E-06	14
Nob1	3,11E-10	0,294697	0,333	0,155	9,67E-06	14
Nedd8	3,16E-10	0,386712	0,667	0,404	9,82E-06	14
Zc3h15	3,21E-10	0,345657	0,522	0,282	9,96E-06	14
Romo1	3,79E-10	0,320911	0,594	0,341	1,18E-05	14
Rad23a	4,05E-10	0,28427	0,42	0,212	1,26E-05	14
Mrps21	4,38E-10	0,306388	0,565	0,31	1,36E-05	14
Tapbp	4,45E-10	0,454077	0,652	0,426	1,38E-05	14
Ywhae	4,69E-10	0,340466	0,551	0,309	1,46E-05	14
Clpp	5,8E-10	0,263347	0,271	0,116	1,8E-05	14
Mrps24	5,84E-10	0,42016	0,556	0,324	1,81E-05	14
Tnfrsf18	5,95E-10	0,483097	0,406	0,217	1,85E-05	14
Sept7	5,97E-10	0,402152	0,556	0,321	1,85E-05	14
Hint1	7,74E-10	0,337445	0,831	0,604	2,4E-05	14
Cox6b1	7,81E-10	0,373143	0,802	0,579	2,43E-05	14
Gbp4	8,06E-10	0,684822	0,329	0,169	2,5E-05	14

Ppp5c	8,33E-10	0,279645	0,261	0,113	2,59E-05	14
Cope	8,36E-10	0,261643	0,507	0,27	2,6E-05	14
Yrdc	8,39E-10	0,337887	0,435	0,231	2,6E-05	14
Bag1	9,21E-10	0,289946	0,527	0,289	2,86E-05	14
Alkbh1	9,24E-10	0,266626	0,382	0,189	2,87E-05	14
Cd52	9,58E-10	0,417574	0,981	0,924	2,97E-05	14
Zap70	1,17E-09	0,342782	0,585	0,333	3,63E-05	14
Ewsr1	1,34E-09	0,315475	0,647	0,393	4,16E-05	14
Polr2g	1,34E-09	0,270257	0,353	0,17	4,17E-05	14
Rpl7a	1,48E-09	0,280487	0,99	0,954	4,59E-05	14
Ikbke	1,5E-09	0,305062	0,348	0,17	4,65E-05	14
Snhg12	1,59E-09	0,372989	0,604	0,363	4,94E-05	14
Hsbp1	1,62E-09	0,272824	0,44	0,224	5,04E-05	14
Dbnl	1,69E-09	0,311503	0,493	0,264	5,24E-05	14
Gm19585	1,9E-09	0,453389	0,333	0,164	5,91E-05	14
Timm10b	1,97E-09	0,323891	0,401	0,206	6,12E-05	14
Dhx15	2,03E-09	0,281189	0,58	0,334	6,31E-05	14
Tgif1	2,08E-09	0,364175	0,454	0,245	6,45E-05	14
Pam16	2,14E-09	0,269272	0,319	0,152	6,66E-05	14
Bzw1	2,18E-09	0,422199	0,628	0,381	6,77E-05	14
Prpf8	2,28E-09	0,321307	0,478	0,26	7,1E-05	14
Psmc2	2,44E-09	0,304328	0,295	0,136	7,58E-05	14
U2surp	2,58E-09	0,260109	0,391	0,196	8E-05	14
Tmed5	2,66E-09	0,276316	0,425	0,221	8,26E-05	14
Pfdn2	2,91E-09	0,361324	0,507	0,285	9,03E-05	14
Ndufa1	3,01E-09	0,39176	0,7	0,455	9,36E-05	14
Srsf10	3,02E-09	0,298959	0,517	0,291	9,37E-05	14
Slc39a7	3,35E-09	0,250665	0,29	0,133	0,000104	14
Pole4	3,37E-09	0,340875	0,56	0,326	0,000105	14
Nubp1	3,76E-09	0,250343	0,353	0,173	0,000117	14
Cox6a1	3,77E-09	0,328808	0,831	0,593	0,000117	14
Hspa4	3,82E-09	0,324507	0,633	0,391	0,000119	14
Nap1l4	4,14E-09	0,298565	0,478	0,262	0,000129	14
Cox5b	4,37E-09	0,401148	0,807	0,629	0,000136	14
Tmem258	4,4E-09	0,367116	0,7	0,45	0,000137	14
Cks2	4,44E-09	0,258881	0,338	0,164	0,000138	14
1110004F	5,2E-09	0,298113	0,473	0,25	0,000162	14
Snu13	5,37E-09	0,406092	0,778	0,548	0,000167	14
Cox8a	5,69E-09	0,340024	0,942	0,802	0,000177	14
Igtp	5,72E-09	0,589977	0,333	0,172	0,000178	14
Fundc2	5,99E-09	0,297872	0,406	0,214	0,000186	14
Sar1a	6,18E-09	0,277003	0,382	0,194	0,000192	14
Srrm2	6,37E-09	0,304281	0,913	0,686	0,000198	14
Rab8b	6,77E-09	0,302436	0,28	0,129	0,00021	14
Polr2c	7,44E-09	0,273921	0,304	0,145	0,000231	14
Ywhah	7,53E-09	0,350572	0,575	0,329	0,000234	14
Got2	7,66E-09	0,270555	0,29	0,134	0,000238	14
Jpt1	8,55E-09	0,332255	0,7	0,447	0,000265	14
Ndufa13	9,37E-09	0,360106	0,816	0,601	0,000291	14
Fam96a	9,82E-09	0,272329	0,425	0,219	0,000305	14
Gtf2f1	9,83E-09	0,262263	0,295	0,138	0,000305	14
Nudt21	1,13E-08	0,281295	0,56	0,32	0,000351	14
Tbl1x	1,25E-08	0,314465	0,275	0,13	0,000387	14
Srgn	1,29E-08	0,704814	0,908	0,856	0,0004	14
Zranb2	1,3E-08	0,2648	0,275	0,128	0,000405	14

Cops9	1,32E-08	0,304079	0,628	0,371	0,00041	14
Csk	1,43E-08	0,361535	0,594	0,363	0,000443	14
Mia2	1,44E-08	0,280511	0,449	0,24	0,000448	14
Swi5	1,64E-08	0,312966	0,527	0,307	0,000509	14
Irf2bp2	1,71E-08	0,342489	0,348	0,181	0,00053	14
Cxcr6	1,78E-08	0,411764	0,319	0,165	0,000553	14
Cenpx	2,2E-08	0,295143	0,444	0,236	0,000683	14
Atp1a1	2,33E-08	0,258385	0,536	0,306	0,000724	14
Aebp2	2,34E-08	0,273255	0,319	0,158	0,000727	14
Rap1a	2,56E-08	0,294877	0,589	0,353	0,000794	14
Mrpl52	2,62E-08	0,320263	0,729	0,509	0,000815	14
Myl12a	2,82E-08	0,297591	0,889	0,657	0,000876	14
Sf3b3	3,16E-08	0,277814	0,522	0,302	0,000982	14
Hnrnpf	3,24E-08	0,286725	0,903	0,732	0,001005	14
Mrps14	3,24E-08	0,255863	0,449	0,246	0,001007	14
Nfatc1	3,31E-08	0,346344	0,348	0,181	0,001027	14
Sap18	3,65E-08	0,319054	0,754	0,533	0,001133	14
Srsf6	4,08E-08	0,337388	0,594	0,383	0,001266	14
Snhg6	4,54E-08	0,272999	0,319	0,163	0,001411	14
Hnrnpa0	4,6E-08	0,286687	0,551	0,325	0,001428	14
Cox7a2	4,91E-08	0,33472	0,763	0,549	0,001524	14
Ddx18	5,24E-08	0,340124	0,396	0,22	0,001626	14
Capza1	5,32E-08	0,271223	0,604	0,367	0,001654	14
Agfg1	5,37E-08	0,261088	0,271	0,13	0,001669	14
Sec61b	5,74E-08	0,323798	0,874	0,668	0,001783	14
Cct4	7,61E-08	0,360463	0,58	0,364	0,002362	14
Tomm20	8,06E-08	0,390397	0,705	0,516	0,002501	14
Ghitm	8,83E-08	0,313411	0,512	0,307	0,002743	14
Cd6	1,03E-07	0,287787	0,531	0,309	0,003202	14
Ube2i	1,03E-07	0,295237	0,667	0,44	0,003205	14
Il2rg	1,3E-07	0,425692	0,913	0,755	0,004033	14
Fkbp1a	1,4E-07	0,323049	0,633	0,407	0,004356	14
Tcea1	1,42E-07	0,283883	0,541	0,323	0,004419	14
AW11201(	1,87E-07	0,782056	0,744	0,639	0,005805	14
Slc3a2	2E-07	0,357948	0,599	0,394	0,006216	14
Atp5f1	2,15E-07	0,269285	0,739	0,492	0,006668	14
Sf3b6	2,26E-07	0,253738	0,643	0,397	0,00702	14
Ncoa3	2,45E-07	0,256789	0,3	0,153	0,007619	14
H2afj	3E-07	0,317597	0,787	0,61	0,009323	14
2010111I0	3,22E-07	0,300923	0,348	0,187	0,009992	14
Ube2l3	3,41E-07	0,341544	0,454	0,274	0,010584	14
Eny2	3,49E-07	0,252341	0,401	0,221	0,010852	14
Ptbp1	3,52E-07	0,295472	0,638	0,409	0,010945	14
Ndufb9	3,96E-07	0,265932	0,541	0,34	0,012298	14
Ndufs5	4,09E-07	0,262068	0,406	0,229	0,012693	14
Spcs2	5,7E-07	0,287538	0,531	0,323	0,0177	14
Nt5c	6,18E-07	0,255248	0,565	0,351	0,019192	14
Psmb4	7,11E-07	0,252643	0,527	0,326	0,022079	14
Ifng	7,36E-07	0,613121	0,261	0,136	0,02285	14
Polr1d	7,41E-07	0,313511	0,787	0,596	0,023007	14
Nr4a2	7,8E-07	0,254074	0,251	0,127	0,024233	14
Ubl5	9,92E-07	0,301963	0,797	0,592	0,030803	14
Psmb8	9,99E-07	0,368466	0,802	0,673	0,031013	14
Capzb	1,06E-06	0,326999	0,792	0,588	0,032931	14
Rnf7	1,15E-06	0,258702	0,425	0,24	0,035718	14

Rbx1	1,18E-06	0,324005	0,556	0,365	0,036593	14
Trac	1,21E-06	0,261456	0,836	0,599	0,037566	14
Eif3i	1,3E-06	0,296328	0,715	0,508	0,040223	14
Rrbp1	1,34E-06	0,296104	0,304	0,165	0,041496	14
Akr1b3	1,38E-06	0,27211	0,546	0,341	0,042765	14
G3bp2	1,55E-06	0,297271	0,488	0,296	0,048097	14
Fcer1g	0	4,102371	0,867	0,026	0	15
Tyrobp	0	3,901889	0,63	0,015	0	15
Ccl3	0	3,089304	0,496	0,017	0	15
Cd244	0	1,467951	0,393	0,002	0	15
Klra7	9,4E-296	2,983716	0,548	0,024	2,9E-291	15
Klra1	1,3E-291	1,903307	0,348	0,009	4,1E-287	15
Gzmb	5,1E-270	4,279243	0,889	0,08	1,6E-265	15
Itgax	1,3E-251	1,143124	0,259	0,005	4E-247	15
Lat2	1,8E-251	1,177941	0,348	0,01	5,6E-247	15
Ccl4	4,5E-241	4,529912	0,756	0,061	1,4E-236	15
Ccdc136	4,1E-221	1,120625	0,281	0,008	1,3E-216	15
Cd160	2,7E-211	2,657833	0,77	0,074	8,4E-207	15
Itga1	1,6E-193	1,798542	0,548	0,038	4,9E-189	15
Xcl1	7,9E-182	3,515836	0,859	0,113	2,4E-177	15
Klre1	4,5E-178	2,298738	0,585	0,048	1,4E-173	15
Gzma	4,1E-140	4,132849	0,459	0,037	1,3E-135	15
Klra9	5,6E-139	1,878528	0,319	0,017	1,7E-134	15
Fgl2	7,7E-137	1,978285	0,563	0,057	2,4E-132	15
Cd7	7,7E-135	4,079361	0,933	0,214	2,4E-130	15
Klrc2	2,9E-115	1,559877	0,43	0,038	9,1E-111	15
Sulf2	9,8E-110	0,868598	0,267	0,015	3E-105	15
Klrb1c	3,2E-102	1,714715	0,57	0,074	9,82E-98	15
Klrk1	1,68E-95	1,897599	0,644	0,104	5,22E-91	15
Emid1	1,79E-92	1,044884	0,267	0,018	5,55E-88	15
Ccl5	1,1E-91	2,500233	1	0,332	3,42E-87	15
Lrrk1	1,39E-89	1,127837	0,311	0,026	4,31E-85	15
Trgv2	6,51E-87	1,614803	0,43	0,05	2,02E-82	15
Tcrg-C4	1,02E-86	1,292873	0,393	0,042	3,17E-82	15
Efhd2	2,48E-80	2,004365	0,837	0,242	7,7E-76	15
Anxa2	7,83E-80	1,708284	0,578	0,098	2,43E-75	15
Rgs1	1,18E-75	2,972625	0,881	0,305	3,66E-71	15
S100a6	2,28E-75	2,311791	0,867	0,244	7,07E-71	15
Nkg7	1,07E-73	1,986091	1	0,466	3,33E-69	15
Osbpl3	1,31E-67	0,966438	0,311	0,033	4,08E-63	15
Gem	3,09E-67	1,963502	0,556	0,107	9,6E-63	15
Litaf	1,69E-62	1,294508	0,333	0,041	5,24E-58	15
Chn2	3,66E-61	1,120236	0,378	0,053	1,14E-56	15
Tcrg-C2	8,45E-60	0,872575	0,296	0,034	2,62E-55	15
Padi2	1,37E-59	1,242579	0,467	0,083	4,24E-55	15
AW11201(	2,71E-57	1,933677	0,97	0,637	8,43E-53	15
Zfp683	2,19E-56	0,980941	0,319	0,041	6,8E-52	15
Ctsw	2,44E-55	1,770737	0,919	0,42	7,58E-51	15
S100a4	6,27E-55	1,413705	0,548	0,113	1,95E-50	15
Klrd1	2,21E-53	1,937622	0,741	0,248	6,87E-49	15
Tnfsf10	7,34E-53	0,968986	0,281	0,035	2,28E-48	15
Ifng	1,48E-52	1,537202	0,578	0,134	4,58E-48	15
Il2rb	4,51E-52	1,581872	0,904	0,375	1,4E-47	15
Slamf7	1,29E-50	1,114653	0,385	0,064	4E-46	15
Abi3	2,4E-48	0,894953	0,341	0,053	7,44E-44	15

Tbx21	6,53E-43	1,092532	0,422	0,087	2,03E-38	15
Tcrg-C1	1,38E-42	1,037275	0,289	0,044	4,28E-38	15
Klrc1	2,81E-40	1,313294	0,341	0,061	8,73E-36	15
AU020206	3,21E-36	1,335323	0,519	0,145	9,97E-32	15
Tmsb4x	5,84E-36	0,754467	1	0,994	1,81E-31	15
Mfsd10	7,55E-35	1,08429	0,385	0,087	2,34E-30	15
Bhlhe40	1,53E-34	1,083796	0,615	0,194	4,74E-30	15
Atp2b4	2,12E-34	0,854505	0,259	0,043	6,59E-30	15
Sytl3	1,47E-33	1,314637	0,563	0,182	4,57E-29	15
H2-Q6	2,41E-33	1,282018	0,904	0,565	7,48E-29	15
Ahnak	5,95E-33	1,19937	0,756	0,307	1,85E-28	15
Chsy1	6,47E-33	1,050483	0,319	0,065	2,01E-28	15
Gm26532	2,81E-32	1,201702	0,304	0,061	8,74E-28	15
Acot7	4,7E-32	1,073799	0,393	0,096	1,46E-27	15
Cd52	9,64E-32	0,878062	0,978	0,925	2,99E-27	15
Pfn1	1,16E-31	0,795616	0,993	0,968	3,6E-27	15
H2-D1	7,9E-31	0,665174	0,993	0,983	2,45E-26	15
Serpina3g	4,55E-30	1,130494	0,319	0,069	1,41E-25	15
Rhob	2,42E-29	1,294553	0,444	0,132	7,5E-25	15
Cxcr6	3,65E-29	1,00186	0,533	0,164	1,13E-24	15
Id2	4,53E-29	1,187788	0,8	0,389	1,41E-24	15
Nr4a2	6,81E-29	1,391347	0,43	0,126	2,12E-24	15
Sh3bgrl3	7,43E-29	0,876686	0,956	0,864	2,31E-24	15
Gimap4	8,39E-28	1,08278	0,911	0,61	2,6E-23	15
H2-K1	1,34E-27	0,634175	0,993	0,982	4,17E-23	15
Cd3g	3,97E-27	1,04336	0,933	0,846	1,23E-22	15
Myo1f	5,31E-27	1,100727	0,393	0,109	1,65E-22	15
Cst7	4,72E-26	1,193552	0,489	0,174	1,47E-21	15
Rgs3	2,57E-25	1,064609	0,415	0,126	7,97E-21	15
Dennd4a	9,83E-24	1,15742	0,585	0,252	3,05E-19	15
Adgre5	1,71E-23	0,970824	0,807	0,456	5,32E-19	15
Itgb2	4,08E-23	1,060329	0,763	0,417	1,27E-18	15
Cox8a	8E-23	0,833312	0,904	0,803	2,48E-18	15
Tigit	2,05E-22	0,776664	0,274	0,065	6,36E-18	15
Ptprcap	2,68E-22	0,920474	0,889	0,635	8,31E-18	15
Hopx	4,24E-22	1,070626	0,607	0,263	1,32E-17	15
Abcb1a	4,88E-22	0,708783	0,252	0,057	1,52E-17	15
Zyx	6,39E-22	0,949935	0,689	0,347	1,99E-17	15
Dusp1	6,67E-22	1,503947	0,815	0,551	2,07E-17	15
Ctsd	9,09E-22	1,036255	0,756	0,433	2,82E-17	15
Gimap7	9,35E-22	1,073286	0,57	0,25	2,9E-17	15
H2-Q7	2,07E-21	0,798575	0,941	0,819	6,42E-17	15
S100a11	1,18E-20	0,907797	0,756	0,43	3,67E-16	15
Btg2	1,28E-20	1,117585	0,741	0,452	3,97E-16	15
Actb	1,51E-20	0,556959	0,993	0,989	4,69E-16	15
Cish	1,7E-20	0,836882	0,363	0,111	5,28E-16	15
Myl6	2,83E-20	0,658692	0,97	0,879	8,78E-16	15
Ikzf2	3,36E-20	0,88373	0,281	0,073	1,04E-15	15
Capzb	5,12E-20	0,923766	0,881	0,588	1,59E-15	15
Nsmaf	1,08E-19	0,811892	0,341	0,105	3,34E-15	15
Ubb	1,51E-19	0,533946	0,978	0,987	4,69E-15	15
Ifitm10	2,63E-19	0,905233	0,378	0,128	8,17E-15	15
Gimap3	3,81E-19	0,778903	0,911	0,673	1,18E-14	15
Serpinb6b	4,87E-19	0,750663	0,259	0,067	1,51E-14	15
Itm2b	3,14E-18	0,801709	0,926	0,831	9,75E-14	15

Gng2	3,59E-18	0,887324	0,363	0,122	1,11E-13	15
Cxcr3	9,08E-18	0,80793	0,37	0,125	2,82E-13	15
Dusp2	1,11E-17	1,095167	0,696	0,395	3,45E-13	15
Rcn1	1,57E-17	0,76833	0,252	0,068	4,88E-13	15
Ucp2	2,37E-17	0,753968	0,859	0,66	7,36E-13	15
Dok2	2,57E-17	0,931407	0,489	0,207	7,99E-13	15
Cyth4	4,38E-17	0,855456	0,489	0,22	1,36E-12	15
Clic1	7,73E-17	0,706589	0,874	0,639	2,4E-12	15
Cd8a	9,35E-17	1,458369	0,504	0,244	2,9E-12	15
Mxd4	1,21E-16	1,062015	0,444	0,186	3,74E-12	15
Lcp1	1,64E-16	0,795722	0,83	0,586	5,1E-12	15
Gpr65	3,45E-16	0,820502	0,363	0,13	1,07E-11	15
Rnf130	3,86E-16	0,768106	0,259	0,076	1,2E-11	15
Srgn	4,3E-16	0,694491	0,933	0,856	1,33E-11	15
Cd3e	6,94E-16	0,71186	0,948	0,896	2,15E-11	15
Trp53i11	8,7E-16	0,62364	0,281	0,087	2,7E-11	15
Trip4	2,26E-15	0,648612	0,259	0,078	7,03E-11	15
Gm19585	3,86E-15	0,799346	0,407	0,164	1,2E-10	15
Ccnd3	4,96E-15	0,757394	0,733	0,442	1,54E-10	15
Ctsa	5,24E-15	0,821024	0,467	0,215	1,63E-10	15
Tbc1d10c	7,18E-15	0,811915	0,652	0,379	2,23E-10	15
Sh2d2a	8,1E-15	0,868947	0,659	0,381	2,52E-10	15
Zfp36	1,36E-14	0,809497	0,726	0,467	4,21E-10	15
Abcb9	1,41E-14	0,658415	0,274	0,088	4,37E-10	15
B2m	1,45E-14	0,481205	0,978	0,98	4,52E-10	15
Fosb	2,05E-14	0,881557	0,511	0,24	6,35E-10	15
Tesc	2,15E-14	0,73557	0,393	0,158	6,68E-10	15
Zap70	2,26E-14	0,788091	0,615	0,334	7,01E-10	15
Sipa1	3,2E-14	0,778833	0,459	0,207	9,94E-10	15
Fos	3,48E-14	1,201589	0,659	0,42	1,08E-09	15
Pglyrp1	5,07E-14	0,833884	0,393	0,164	1,58E-09	15
Glrx	7,04E-14	0,664769	0,281	0,094	2,19E-09	15
Lax1	7,34E-14	0,799208	0,319	0,117	2,28E-09	15
Lrmp	8,24E-14	0,725215	0,267	0,087	2,56E-09	15
Crip1	8,69E-14	0,62556	0,889	0,747	2,7E-09	15
Apobec3	9,34E-14	0,796091	0,489	0,24	2,9E-09	15
Pla2g16	9,53E-14	0,668546	0,496	0,238	2,96E-09	15
Arhgap9	1,05E-13	0,877231	0,57	0,32	3,27E-09	15
Ppp3cc	1,06E-13	0,697858	0,459	0,219	3,28E-09	15
Psmb8	1,44E-13	0,643104	0,859	0,673	4,47E-09	15
Peak1	1,66E-13	0,687734	0,311	0,112	5,17E-09	15
Hcst	1,95E-13	0,68542	0,896	0,791	6,05E-09	15
Ptprc	2,17E-13	0,681942	0,889	0,787	6,74E-09	15
Rac2	2,5E-13	0,453526	0,956	0,913	7,75E-09	15
Txnrd1	1,8E-12	0,663052	0,267	0,093	5,59E-08	15
Cd226	1,93E-12	0,793848	0,348	0,142	5,98E-08	15
H2-Q4	2,91E-12	0,709008	0,837	0,698	9,04E-08	15
Sorl1	2,97E-12	0,885542	0,363	0,16	9,23E-08	15
Epsti1	3,33E-12	0,719241	0,674	0,418	1,03E-07	15
Serf2	3,54E-12	0,44602	0,978	0,906	1,1E-07	15
H3f3b	4,01E-12	0,559016	0,985	0,977	1,25E-07	15
Zfp36l2	4,13E-12	0,817374	0,867	0,68	1,28E-07	15
Ikzf3	4,47E-12	0,739929	0,4	0,182	1,39E-07	15
Jun	4,56E-12	1,231986	0,733	0,529	1,42E-07	15
Itgal	5,28E-12	0,766375	0,585	0,34	1,64E-07	15

Prkar1a	5,98E-12	0,677679	0,704	0,472	1,86E-07	15
Malat1	9,46E-12	0,635142	1	0,984	2,94E-07	15
Fasl	1,12E-11	0,513334	0,252	0,085	3,48E-07	15
Gpx4	1,16E-11	0,726356	0,711	0,491	3,61E-07	15
Sh2d3c	1,16E-11	0,666675	0,274	0,102	3,62E-07	15
Rinl	1,25E-11	0,59865	0,57	0,308	3,88E-07	15
Ddit3	1,42E-11	0,876759	0,348	0,152	4,4E-07	15
Mbnl1	1,44E-11	0,694608	0,874	0,702	4,47E-07	15
Rps6ka1	1,45E-11	0,782674	0,407	0,191	4,49E-07	15
Rgs2	2,73E-11	1,002473	0,511	0,298	8,48E-07	15
Pik3r1	3E-11	0,779126	0,415	0,204	9,31E-07	15
Twf2	3,42E-11	0,670692	0,437	0,217	1,06E-06	15
Sqstm1	3,62E-11	0,86245	0,711	0,547	1,12E-06	15
Tmed5	3,96E-11	0,643888	0,444	0,222	1,23E-06	15
H2-T23	4E-11	0,756489	0,741	0,595	1,24E-06	15
Cyba	4,42E-11	0,588784	0,844	0,655	1,37E-06	15
Apol7e	4,76E-11	0,547988	0,267	0,099	1,48E-06	15
Jund	4,85E-11	0,634917	0,97	0,903	1,51E-06	15
Slc28a2	4,94E-11	0,605854	0,319	0,133	1,53E-06	15
Ndfip1	6,71E-11	0,605331	0,741	0,53	2,08E-06	15
Prex1	6,91E-11	0,68711	0,481	0,247	2,14E-06	15
Fnbp1	8,73E-11	0,71973	0,489	0,262	2,71E-06	15
Cd37	8,96E-11	0,671284	0,719	0,518	2,78E-06	15
Ndufa13	1,09E-10	0,616564	0,763	0,602	3,39E-06	15
Map3k8	1,13E-10	0,722628	0,259	0,097	3,5E-06	15
Neurl3	1,32E-10	0,787028	0,385	0,183	4,1E-06	15
Mob3a	1,47E-10	0,632513	0,363	0,167	4,57E-06	15
Ppp1r18	2,63E-10	0,624398	0,763	0,587	8,17E-06	15
Actg1	3,37E-10	0,47831	0,978	0,928	1,05E-05	15
Ly6e	5,87E-10	0,524536	0,896	0,801	1,82E-05	15
Ech1	7,44E-10	0,553292	0,348	0,16	2,31E-05	15
Capns1	7,87E-10	0,709886	0,533	0,32	2,44E-05	15
Tab2	8,07E-10	0,59546	0,319	0,137	2,51E-05	15
Gnas	8,41E-10	0,603168	0,793	0,699	2,61E-05	15
Runx3	8,98E-10	0,705977	0,415	0,214	2,79E-05	15
Gbp4	1,04E-09	0,711429	0,356	0,169	3,22E-05	15
Rin3	1,12E-09	0,580066	0,296	0,126	3,47E-05	15
Gimap8	1,6E-09	0,762564	0,437	0,236	4,97E-05	15
Calm1	2,28E-09	0,524522	0,904	0,81	7,07E-05	15
Mef2a	2,67E-09	0,62468	0,296	0,131	8,28E-05	15
Gimap5	2,67E-09	0,760263	0,637	0,449	8,28E-05	15
Aes	2,72E-09	0,638028	0,733	0,603	8,45E-05	15
Gadd45b	3,22E-09	0,799265	0,378	0,18	0,0001	15
Wbp1	3,58E-09	0,572477	0,259	0,106	0,000111	15
Ost4	3,87E-09	0,590247	0,726	0,544	0,00012	15
Prdx5	3,89E-09	0,671129	0,496	0,299	0,000121	15
Os9	4,13E-09	0,54146	0,393	0,201	0,000128	15
Arhgdia	4,13E-09	0,552567	0,704	0,523	0,000128	15
Actr3	4,42E-09	0,52015	0,837	0,688	0,000137	15
Nedd9	5,26E-09	0,684227	0,37	0,184	0,000163	15
Pitpnc1	5,65E-09	0,59754	0,459	0,251	0,000175	15
Ppil2	6,32E-09	0,610472	0,348	0,171	0,000196	15
Acap1	8,57E-09	0,68153	0,548	0,345	0,000266	15
Anxa11	8,65E-09	0,617955	0,363	0,184	0,000269	15
Rbl2	8,71E-09	0,597587	0,326	0,152	0,00027	15

Nbeal2	9,07E-09	0,544719	0,267	0,112	0,000282	15
Atf4	9,27E-09	0,672079	0,607	0,431	0,000288	15
Fkbp8	1,01E-08	0,539595	0,541	0,334	0,000315	15
Tln1	1,14E-08	0,617101	0,615	0,43	0,000353	15
Rnaset2b	1,25E-08	0,595863	0,526	0,329	0,000389	15
Atp6v0e	1,37E-08	0,563881	0,541	0,33	0,000424	15
Cox17	1,5E-08	0,613591	0,563	0,366	0,000465	15
Ier2	1,53E-08	0,664843	0,756	0,609	0,000476	15
Il10rb	1,56E-08	0,509053	0,259	0,11	0,000485	15
Junb	1,64E-08	0,486791	0,956	0,96	0,00051	15
Grina	1,77E-08	0,59467	0,319	0,151	0,000551	15
Lamtor4	1,93E-08	0,647924	0,378	0,201	0,000599	15
Ifnar2	2,17E-08	0,520577	0,319	0,151	0,000674	15
Elob	2,28E-08	0,543513	0,785	0,653	0,000707	15
Ptpn22	2,57E-08	0,568379	0,511	0,313	0,000798	15
Prr13	2,72E-08	0,507666	0,511	0,304	0,000845	15
Ccnd2	2,78E-08	0,56608	0,637	0,447	0,000864	15
H2-T22	2,9E-08	0,596309	0,756	0,547	0,000899	15
Socs1	3,22E-08	0,643833	0,444	0,252	0,001	15
Mndal	3,43E-08	0,619836	0,511	0,314	0,001064	15
Spcs2	3,51E-08	0,542039	0,511	0,324	0,00109	15
Klf10	3,9E-08	0,662141	0,252	0,108	0,00121	15
Prkacb	3,94E-08	0,531276	0,385	0,203	0,001224	15
Rab37	4,85E-08	0,624012	0,259	0,118	0,001507	15
Ubl5	4,93E-08	0,535201	0,726	0,593	0,001531	15
Slc9a3r1	5,47E-08	0,538707	0,556	0,35	0,001698	15
Tbcb	6,35E-08	0,604859	0,274	0,127	0,001971	15
Cdc42	7,03E-08	0,446841	0,815	0,665	0,002182	15
Sept1	8,78E-08	0,466026	0,852	0,733	0,002727	15
Pglyrp2	8,91E-08	0,483982	0,281	0,128	0,002767	15
Dcaf7	9,5E-08	0,56292	0,311	0,153	0,002952	15
Midn	1,08E-07	0,608538	0,267	0,125	0,003362	15
Akna	1,3E-07	0,533673	0,481	0,294	0,004045	15
Ubc	1,35E-07	0,425153	0,889	0,845	0,004193	15
Ntan1	1,38E-07	0,709702	0,341	0,18	0,004278	15
Taf10	1,41E-07	0,599345	0,563	0,379	0,004366	15
Fth1	1,53E-07	0,481691	0,956	0,969	0,004762	15
Cfl1	1,61E-07	0,387235	0,919	0,924	0,004987	15
Sema4d	1,8E-07	0,534999	0,422	0,243	0,005586	15
Pde7a	1,87E-07	0,653	0,37	0,199	0,005795	15
Gbp8	1,87E-07	0,505907	0,259	0,115	0,005807	15
Uqcrb	2,22E-07	0,446964	0,593	0,417	0,00689	15
Sft2d1	2,51E-07	0,49273	0,363	0,192	0,007782	15
Atp6v0a2	2,51E-07	0,546506	0,274	0,131	0,007808	15
Clec2d	2,57E-07	0,589905	0,459	0,269	0,007981	15
Klf6	3,21E-07	0,666961	0,689	0,553	0,009967	15
Tmem256	3,24E-07	0,533551	0,289	0,142	0,010047	15
S100a10	3,6E-07	0,270984	0,815	0,628	0,011192	15
Lrp10	4,08E-07	0,601268	0,43	0,264	0,012674	15
Tmbim6	4,19E-07	0,506056	0,726	0,571	0,013006	15
H2afy	4,2E-07	0,54231	0,644	0,492	0,013028	15
Isg15	4,22E-07	0,657813	0,267	0,126	0,013093	15
Selplg	4,45E-07	0,445097	0,83	0,68	0,013813	15
Tpi1	4,56E-07	0,468254	0,252	0,116	0,014149	15
Rab8b	4,87E-07	0,569198	0,274	0,13	0,015125	15

Lcp2	6,13E-07	0,608142	0,511	0,337	0,019037	15
Ifngr1	6,18E-07	0,548228	0,667	0,473	0,019197	15
Cox6c	7,98E-07	0,429441	0,8	0,726	0,024793	15
Tsc22d4	8,37E-07	0,48594	0,533	0,35	0,025998	15
Tma7	9,11E-07	0,471434	0,77	0,602	0,0283	15
Arpc1b	9,15E-07	0,456253	0,852	0,723	0,028412	15
Ndufa4	9,67E-07	0,464147	0,748	0,59	0,030033	15
Ptk2b	9,86E-07	0,547139	0,378	0,215	0,030607	15
Vps28	1,06E-06	0,527784	0,607	0,431	0,032788	15
Esyt1	1,06E-06	0,594511	0,496	0,317	0,032859	15
Krtcap2	1,12E-06	0,521553	0,659	0,496	0,034749	15
Tnfrsf18	1,18E-06	0,566392	0,378	0,218	0,036686	15
Ddx17	1,19E-06	0,402591	0,333	0,173	0,037028	15
Mea1	1,2E-06	0,58203	0,326	0,177	0,037278	15
Vamp8	1,24E-06	0,564043	0,481	0,312	0,038404	15
Lgals1	1,27E-06	0,464186	0,578	0,383	0,03934	15
Tapbp	1,27E-06	0,566007	0,593	0,427	0,039495	15
Atp5j2	1,33E-06	0,474317	0,756	0,658	0,041187	15
Card19	1,33E-06	0,47788	0,274	0,137	0,04136	15
Tmem59	1,36E-06	0,533866	0,474	0,298	0,042128	15
Atp6v1f	1,37E-06	0,490451	0,667	0,497	0,042611	15
Sem1	1,39E-06	0,363784	0,815	0,672	0,043089	15
Syngr2	1,56E-06	0,383651	0,274	0,133	0,048386	15

Supplemental Table 3. List of significant NKT cell markers for each of the time points compared to control.

gene	p_val	avg_log2FC	pct.1	pct.2	p_val_adj	comparison
Uba52	1,2804E-86	1,54465748	0,995	0,974	3,9761E-82	D10 vs Control
Nfkbia	1,1668E-63	2,35056219	0,933	0,456	3,6233E-59	D10 vs Control
Tnfaip3	3,0788E-59	2,17461954	0,902	0,407	9,5607E-55	D10 vs Control
Zfp36	3,2227E-53	2,18623354	0,891	0,418	1,0007E-48	D10 vs Control
Csrnp1	7,561E-53	1,5720206	0,663	0,132	2,3479E-48	D10 vs Control
Gm10076	5,0592E-50	1,05522991	0,383	0,016	1,571E-45	D10 vs Control
Rpl13a	1,4864E-49	1,03072015	0,969	0,946	4,6157E-45	D10 vs Control
Nr4a1	6,0093E-49	1,77908413	0,591	0,112	1,8661E-44	D10 vs Control
Atf3	3,1576E-46	1,73704614	0,316	0,005	9,8052E-42	D10 vs Control
Vps37b	3,2719E-45	1,77017657	0,829	0,367	1,016E-40	D10 vs Control
Vim	5,0019E-45	1,97891323	0,642	0,156	1,5532E-40	D10 vs Control
Ifrd1	1,4552E-39	1,58877067	0,617	0,166	4,5188E-35	D10 vs Control
Capg	1,5058E-39	1,37366769	0,497	0,086	4,6761E-35	D10 vs Control
Cd7	2,0002E-38	1,16770961	0,756	0,243	6,2112E-34	D10 vs Control
mt-Atp8	4,3459E-37	1,13019577	0,979	0,946	1,3495E-32	D10 vs Control
Gapdh	1,0778E-36	1,09194322	0,969	0,858	3,3468E-32	D10 vs Control
Ier2	1,8005E-36	1,45173087	0,891	0,504	5,591E-32	D10 vs Control
Klra9	7,0528E-34	1,39854456	0,383	0,05	2,1901E-29	D10 vs Control
Tgif1	4,9457E-33	1,01375583	0,342	0,039	1,5358E-28	D10 vs Control
Nfkbiz	6,127E-33	1,27881195	0,565	0,156	1,9026E-28	D10 vs Control
Fosl2	3,4711E-30	1,08842732	0,399	0,068	1,0779E-25	D10 vs Control
Hspa8	8,239E-30	0,84254493	0,984	0,911	2,5585E-25	D10 vs Control
Junb	1,637E-29	1,04027028	0,995	0,907	5,0834E-25	D10 vs Control
Dusp1	5,6706E-29	1,4861331	0,896	0,583	1,7609E-24	D10 vs Control
Pnrc1	1,0652E-28	0,9201764	0,964	0,816	3,3077E-24	D10 vs Control
Ppp1r15a	1,5284E-28	1,22382254	0,829	0,54	4,746E-24	D10 vs Control
S100a6	1,0743E-27	1,3841662	0,87	0,577	3,336E-23	D10 vs Control
Gem	8,2356E-27	1,2457332	0,451	0,107	2,5574E-22	D10 vs Control
Ly6c2	9,5803E-27	1,25612825	0,762	0,381	2,975E-22	D10 vs Control
Pim1	1,901E-26	1,45770715	0,736	0,4	5,9031E-22	D10 vs Control
Nme2	1,6789E-25	1,04272528	0,819	0,521	5,2134E-21	D10 vs Control
Ccl5	1,1787E-24	1,6225392	0,881	0,751	3,6603E-20	D10 vs Control
Gadd45b	1,737E-24	1,29377291	0,425	0,11	5,3939E-20	D10 vs Control
Maff	1,9684E-24	1,02365502	0,352	0,07	6,1124E-20	D10 vs Control
Dusp5	2,0631E-24	1,37554304	0,653	0,313	6,4066E-20	D10 vs Control
Rplp0	2,2547E-24	0,55467837	0,995	0,978	7,0015E-20	D10 vs Control
Ftl1	6,7426E-24	0,91184602	0,974	0,927	2,0938E-19	D10 vs Control
Slc38a2	3,317E-23	1,0328308	0,746	0,389	1,03E-18	D10 vs Control
Fcer1g	4,8679E-23	0,92731286	0,42	0,098	1,5116E-18	D10 vs Control
Gzmb	1,7401E-22	1,57864606	0,617	0,255	5,4035E-18	D10 vs Control
Nfkbid	2,9882E-22	1,09527382	0,503	0,17	9,2793E-18	D10 vs Control
Hsp90ab1	3,8059E-22	0,73971283	0,964	0,894	1,1818E-17	D10 vs Control
Eif5a	3,9717E-22	0,86772583	0,907	0,723	1,2333E-17	D10 vs Control
Eef1a1	3,9908E-22	0,3850494	0,99	0,997	1,2392E-17	D10 vs Control
Klf2	4,4823E-21	1,03685111	0,648	0,274	1,3919E-16	D10 vs Control
Anxa2	4,5495E-21	0,98698078	0,482	0,156	1,4128E-16	D10 vs Control
Erh	6,0386E-21	0,74790273	0,482	0,154	1,8752E-16	D10 vs Control
Ifng	6,4169E-21	1,33460096	0,637	0,319	1,9927E-16	D10 vs Control
Dennd4a	8,424E-21	1,00874235	0,627	0,28	2,6159E-16	D10 vs Control
Rps2	1,0816E-20	0,50504738	0,984	0,984	3,3586E-16	D10 vs Control
Zc3h12a	1,1067E-20	0,86509855	0,42	0,118	3,4365E-16	D10 vs Control
Gpr183	1,821E-20	0,81335425	0,285	0,053	5,6547E-16	D10 vs Control
Emp3	2,1196E-20	1,07184337	0,689	0,342	6,582E-16	D10 vs Control

Rpl36al	2,7811E-20	0,68567119	0,943	0,757	8,6362E-16	D10 vs Control
Rasgrp2	9,5434E-20	0,92136365	0,477	0,16	2,9635E-15	D10 vs Control
Rgcc	1,893E-19	0,98555579	0,342	0,084	5,8782E-15	D10 vs Control
Tagap	2,4725E-19	0,81629709	0,337	0,081	7,6779E-15	D10 vs Control
Lgals1	5,7293E-19	1,03018238	0,813	0,566	1,7791E-14	D10 vs Control
Pim3	8,9152E-19	0,88873893	0,332	0,084	2,7684E-14	D10 vs Control
Ier5	3,9975E-18	1,01241437	0,762	0,462	1,2413E-13	D10 vs Control
Bhlhe40	6,574E-18	1,15167601	0,637	0,341	2,0414E-13	D10 vs Control
Sidt1	7,9991E-17	0,81777702	0,311	0,079	2,4839E-12	D10 vs Control
Ccrl2	9,7256E-17	1,07630135	0,254	0,053	3,0201E-12	D10 vs Control
Crip1	1,1682E-16	0,9597265	0,922	0,751	3,6277E-12	D10 vs Control
Sytl3	1,3359E-16	0,8506697	0,596	0,291	4,1483E-12	D10 vs Control
Arf4	5,0325E-16	0,78748445	0,72	0,403	1,5627E-11	D10 vs Control
Klf10	7,3794E-16	0,75680706	0,259	0,058	2,2915E-11	D10 vs Control
Zfp36l1	1,0521E-15	0,95291151	0,653	0,351	3,267E-11	D10 vs Control
Oser1	1,4967E-15	0,70363089	0,337	0,1	4,6478E-11	D10 vs Control
Rpsa	2,0201E-15	0,34945691	0,995	0,989	6,2732E-11	D10 vs Control
Kdm6b	2,1324E-15	0,78945249	0,44	0,173	6,6217E-11	D10 vs Control
Per1	2,3769E-15	0,78117376	0,425	0,152	7,381E-11	D10 vs Control
Cd69	3,5163E-15	0,92578589	0,606	0,308	1,0919E-10	D10 vs Control
Actg1	6,4601E-15	0,52535515	0,984	0,947	2,0061E-10	D10 vs Control
Rgs2	4,6839E-14	0,90117426	0,503	0,224	1,4545E-09	D10 vs Control
Dusp10	9,2394E-14	0,76209298	0,275	0,078	2,8691E-09	D10 vs Control
Klrk1	9,8689E-14	0,70904497	0,725	0,404	3,0646E-09	D10 vs Control
Zyx	1,0881E-13	0,69720641	0,777	0,493	3,379E-09	D10 vs Control
Odc1	1,8349E-13	1,02854664	0,425	0,188	5,6979E-09	D10 vs Control
Egr1	2,1509E-13	0,51225597	0,383	0,137	6,6793E-09	D10 vs Control
Nme1	3,2089E-13	0,6923084	0,487	0,219	9,9647E-09	D10 vs Control
Klrc2	3,9039E-13	0,66519266	0,435	0,17	1,2123E-08	D10 vs Control
Pabpc1	8,3774E-13	0,4827295	0,974	0,905	2,6014E-08	D10 vs Control
Ass1	1,0557E-12	0,68150486	0,326	0,114	3,2782E-08	D10 vs Control
Zfp36l2	1,1193E-12	0,88800139	0,876	0,731	3,4759E-08	D10 vs Control
H2afz	1,2621E-12	0,72791688	0,751	0,54	3,9192E-08	D10 vs Control
Il21r	3,141E-12	0,64203926	0,508	0,247	9,7538E-08	D10 vs Control
Rpl3	4,511E-12	0,38831968	0,984	0,966	1,4008E-07	D10 vs Control
Rpl15	4,8105E-12	0,42487899	0,984	0,958	1,4938E-07	D10 vs Control
Hexim1	6,2451E-12	0,66448988	0,295	0,098	1,9393E-07	D10 vs Control
Tob1	7,4909E-12	0,61849025	0,311	0,107	2,3261E-07	D10 vs Control
Ywhaq	7,545E-12	0,66956659	0,539	0,283	2,3429E-07	D10 vs Control
Rbm3	1,1627E-11	0,53264471	0,891	0,782	3,6106E-07	D10 vs Control
Sertad1	1,218E-11	0,63682461	0,482	0,219	3,7822E-07	D10 vs Control
Mxd1	1,2573E-11	0,54379623	0,269	0,082	3,9044E-07	D10 vs Control
Tagln2	1,3919E-11	0,69139349	0,824	0,649	4,3222E-07	D10 vs Control
Arl4c	2,2374E-11	0,61879235	0,606	0,337	6,9478E-07	D10 vs Control
Eef2	3,5422E-11	0,3720823	0,99	0,949	1,1E-06	D10 vs Control
Hspa5	3,6002E-11	0,59089025	0,834	0,652	1,118E-06	D10 vs Control
Cebpb	4,1595E-11	0,57286286	0,575	0,303	1,2916E-06	D10 vs Control
Sys1	4,1866E-11	0,51965488	0,301	0,104	1,3001E-06	D10 vs Control
Map1lc3b	4,5795E-11	0,57882264	0,684	0,418	1,4221E-06	D10 vs Control
S100a4	5,9519E-11	0,86195821	0,404	0,182	1,8482E-06	D10 vs Control
Eif1	8,5379E-11	0,33310318	0,979	0,984	2,6513E-06	D10 vs Control
Rpl35	9,3587E-11	0,38237369	0,974	0,966	2,9062E-06	D10 vs Control
Tcf7	1,0634E-10	0,58946208	0,399	0,173	3,302E-06	D10 vs Control
Rpl14	1,0904E-10	0,33061384	0,984	0,963	3,3861E-06	D10 vs Control
Rpl29	1,2025E-10	0,35045869	0,995	0,967	3,7342E-06	D10 vs Control

Ptma	1,5082E-10	0,37938686	0,979	0,967	4,6834E-06	D10 vs Control
Cks2	1,6386E-10	0,52664134	0,28	0,095	5,0883E-06	D10 vs Control
H3f3b	1,8258E-10	0,42917022	0,974	0,967	5,6696E-06	D10 vs Control
Rbm38	2,205E-10	0,65234622	0,549	0,306	6,8471E-06	D10 vs Control
Flna	2,5347E-10	0,56152272	0,518	0,263	7,8711E-06	D10 vs Control
Ddit3	3,2122E-10	0,6268267	0,337	0,135	9,9749E-06	D10 vs Control
Dnajb1	4,2449E-10	0,54239098	0,368	0,154	1,3182E-05	D10 vs Control
Bin2	4,9799E-10	0,59207854	0,777	0,569	1,5464E-05	D10 vs Control
Atf4	5,4239E-10	0,65227373	0,642	0,395	1,6843E-05	D10 vs Control
Dnajb9	7,0665E-10	0,55437718	0,311	0,123	2,1944E-05	D10 vs Control
Dynll1	9,9842E-10	0,57149995	0,632	0,376	3,1004E-05	D10 vs Control
Atp1b3	1,1492E-09	0,5476492	0,513	0,278	3,5686E-05	D10 vs Control
Rara	1,1531E-09	0,61039613	0,264	0,093	3,5808E-05	D10 vs Control
Yrdc	1,1886E-09	0,46176494	0,368	0,157	3,6911E-05	D10 vs Control
Rpl8	1,2509E-09	0,28421604	0,99	0,989	3,8846E-05	D10 vs Control
Gna13	1,5362E-09	0,59624823	0,503	0,275	4,7703E-05	D10 vs Control
Npm1	1,8789E-09	0,52816867	0,86	0,762	5,8344E-05	D10 vs Control
Slc25a4	2,4897E-09	0,47613829	0,264	0,095	7,7312E-05	D10 vs Control
Glul	3,0461E-09	0,43808727	0,275	0,098	9,4592E-05	D10 vs Control
Srsf2	3,3398E-09	0,59721996	0,72	0,519	0,00010371	D10 vs Control
Gpr132	4,8034E-09	0,58005013	0,513	0,291	0,00014916	D10 vs Control
Cited2	4,8736E-09	0,83865774	0,492	0,264	0,00015134	D10 vs Control
Ptbp1	5,0609E-09	0,5626193	0,503	0,283	0,00015716	D10 vs Control
Serpinb6b	5,5851E-09	0,56843642	0,368	0,168	0,00017344	D10 vs Control
Cytip	6,4999E-09	0,58475393	0,575	0,347	0,00020184	D10 vs Control
Sqstm1	8,4386E-09	0,54756135	0,689	0,47	0,00026204	D10 vs Control
Btg2	1,0205E-08	0,69447765	0,715	0,561	0,00031688	D10 vs Control
Fos	1,0705E-08	0,62663604	0,611	0,378	0,00033242	D10 vs Control
Il7r	1,283E-08	0,54356685	0,601	0,361	0,0003984	D10 vs Control
Serpinb9	1,3706E-08	0,49434778	0,254	0,092	0,00042562	D10 vs Control
Dnaja1	1,4426E-08	0,54560138	0,653	0,42	0,00044796	D10 vs Control
Gramd3	1,4536E-08	0,53794723	0,596	0,37	0,00045138	D10 vs Control
Hnrnpdl	2,3636E-08	0,47845458	0,674	0,426	0,00073396	D10 vs Control
Tnf	4,193E-08	0,44338852	0,259	0,1	0,00130204	D10 vs Control
Wsb1	4,8588E-08	0,48480075	0,28	0,115	0,0015088	D10 vs Control
Arid5a	6,3186E-08	0,38189899	0,259	0,101	0,00196213	D10 vs Control
Ube2d3	6,8865E-08	0,42001879	0,845	0,711	0,00213847	D10 vs Control
Slc3a2	1,6249E-07	0,50308175	0,611	0,414	0,00504578	D10 vs Control
Chd7	1,6726E-07	0,50755987	0,404	0,21	0,00519401	D10 vs Control
Vasp	1,9853E-07	0,45591591	0,694	0,505	0,00616485	D10 vs Control
Ezr	1,9906E-07	0,44293257	0,637	0,406	0,00618132	D10 vs Control
Sub1	2,0961E-07	0,47023428	0,86	0,736	0,00650902	D10 vs Control
Ahnak	2,1757E-07	0,46999781	0,819	0,596	0,00675623	D10 vs Control
Rpl7a	2,2558E-07	0,30483691	0,984	0,953	0,00700497	D10 vs Control
Dazap2	2,3603E-07	0,51517266	0,741	0,535	0,00732933	D10 vs Control
Batf	3,1046E-07	0,44127857	0,28	0,124	0,00964065	D10 vs Control
H3f3a	3,1116E-07	0,36542197	0,922	0,806	0,0096624	D10 vs Control
Srsf3	3,811E-07	0,5069319	0,689	0,533	0,01183439	D10 vs Control
Plk3	3,8842E-07	0,42287176	0,254	0,106	0,01206147	D10 vs Control
Snrpd1	4,2102E-07	0,47417282	0,492	0,286	0,01307379	D10 vs Control
Pag1	4,2918E-07	0,43505737	0,332	0,159	0,0133273	D10 vs Control
Ptp4a1	6,543E-07	0,5011508	0,42	0,235	0,02031788	D10 vs Control
Pik3r5	7,0016E-07	0,47558864	0,275	0,126	0,02174196	D10 vs Control
Traf1	8,0476E-07	0,51822754	0,451	0,269	0,02499015	D10 vs Control
Taf1d	8,6313E-07	0,51736536	0,472	0,289	0,02680264	D10 vs Control

Myo1g	1,1166E-06	0,49296581	0,383	0,205	0,03467365	D10 vs Control
Ly6c2	1,9622E-30	1,44792903	0,762	0,295	6,0931E-26	D21 vs Control
Capg	5,7755E-29	1,21105456	0,497	0,098	1,7935E-24	D21 vs Control
Klra9	1,2801E-27	1,35386103	0,383	0,049	3,9752E-23	D21 vs Control
Cd7	1,0168E-26	0,79956513	0,756	0,276	3,1574E-22	D21 vs Control
Vim	2,3858E-17	1,11929751	0,642	0,299	7,4087E-13	D21 vs Control
S100a6	6,0213E-17	1,08289957	0,87	0,643	1,8698E-12	D21 vs Control
Klrk1	1,2273E-16	0,82370887	0,725	0,35	3,8113E-12	D21 vs Control
Ccl5	8,3563E-16	1,08367729	0,881	0,771	2,5949E-11	D21 vs Control
Rpl14	7,3872E-15	0,4313259	0,984	0,949	2,2939E-10	D21 vs Control
Zfp36	1,0831E-14	0,80717904	0,891	0,662	3,3633E-10	D21 vs Control
Rplp0	2,3187E-14	0,40642408	0,995	0,985	7,2001E-10	D21 vs Control
Arl4c	2,5778E-14	0,74157358	0,606	0,276	8,0048E-10	D21 vs Control
Fcer1g	2,667E-14	0,84847347	0,42	0,132	8,2817E-10	D21 vs Control
Gzmb	2,8306E-14	1,04404957	0,617	0,299	8,7898E-10	D21 vs Control
Rasgrp2	3,1199E-14	0,74474449	0,477	0,177	9,6884E-10	D21 vs Control
mt-Co2	6,7925E-14	0,33658675	0,995	0,994	2,1093E-09	D21 vs Control
Zyx	9,2978E-14	0,71467594	0,777	0,481	2,8872E-09	D21 vs Control
Nfkbia	9,9841E-14	0,75160162	0,933	0,776	3,1003E-09	D21 vs Control
Pim1	2,6993E-13	0,91634134	0,736	0,487	8,3821E-09	D21 vs Control
Lgals1	5,3676E-12	0,83892704	0,813	0,605	1,6668E-07	D21 vs Control
Rps18	1,5783E-11	0,38349539	0,974	0,957	4,9011E-07	D21 vs Control
mt-Atp8	1,7546E-11	0,41959288	0,979	0,985	5,4484E-07	D21 vs Control
Rpl13	2,4849E-11	0,26969912	0,995	0,994	7,7165E-07	D21 vs Control
Tgif1	2,7338E-11	0,64157315	0,342	0,118	8,4893E-07	D21 vs Control
Klrc2	4,9135E-11	0,53636909	0,435	0,175	1,5258E-06	D21 vs Control
Rps2	6,0659E-11	0,34770642	0,984	0,989	1,8837E-06	D21 vs Control
Crip1	8,182E-11	0,77059251	0,922	0,763	2,5408E-06	D21 vs Control
Emp3	1,4205E-10	0,72517779	0,689	0,408	4,4111E-06	D21 vs Control
Atf3	1,5756E-10	0,75964129	0,316	0,105	4,8927E-06	D21 vs Control
Rpl35	3,4568E-10	0,39018552	0,974	0,966	1,0734E-05	D21 vs Control
Gpr183	4,0564E-10	0,59370772	0,285	0,092	1,2596E-05	D21 vs Control
Rps19	5,1424E-10	0,37649372	0,979	0,968	1,5969E-05	D21 vs Control
Rpl29	6,4567E-10	0,36147291	0,995	0,962	2,005E-05	D21 vs Control
Anxa2	8,3982E-10	0,61128006	0,482	0,226	2,6079E-05	D21 vs Control
Klrd1	2,727E-09	0,56416215	0,601	0,331	8,468E-05	D21 vs Control
Nme2	4,6121E-09	0,53375541	0,819	0,62	0,00014322	D21 vs Control
Klf2	6,9187E-09	0,53649229	0,648	0,38	0,00021485	D21 vs Control
Serpinb6b	1,1232E-08	0,55599775	0,368	0,16	0,00034879	D21 vs Control
Tnfaip3	1,2662E-08	0,51192621	0,902	0,748	0,0003932	D21 vs Control
Tmsb4x	1,349E-08	0,27050891	0,995	0,991	0,00041892	D21 vs Control
Klre1	1,3733E-08	0,54308026	0,368	0,156	0,00042646	D21 vs Control
Rpl4	1,4151E-08	0,41883876	0,943	0,853	0,00043944	D21 vs Control
H2afz	1,6323E-08	0,57346227	0,751	0,551	0,00050688	D21 vs Control
Rplp1	1,643E-08	0,28503201	0,99	0,987	0,00051021	D21 vs Control
Flna	2,1012E-08	0,47677954	0,518	0,267	0,00065249	D21 vs Control
Rpl12	2,7961E-08	0,33647397	0,959	0,947	0,00086827	D21 vs Control
Sidt1	4,1079E-08	0,55593187	0,311	0,122	0,00127561	D21 vs Control
Rpl32	4,1326E-08	0,30723644	0,984	0,991	0,0012833	D21 vs Control
Rpl23a	8,6943E-08	0,40611809	0,938	0,84	0,00269985	D21 vs Control
Bin2	9,1512E-08	0,47066453	0,777	0,547	0,00284174	D21 vs Control
Rpl15	9,4983E-08	0,34680582	0,984	0,951	0,0029495	D21 vs Control
Anxa1	9,935E-08	0,47012926	0,295	0,12	0,0030851	D21 vs Control
Rps12	1,7521E-07	0,34607421	0,974	0,951	0,00544067	D21 vs Control
Rara	2,0235E-07	0,56560409	0,264	0,103	0,00628356	D21 vs Control

Il18r1	2,1743E-07	0,51309638	0,332	0,15	0,00675174	D21 vs Control
Il21r	2,4478E-07	0,43260471	0,508	0,28	0,00760103	D21 vs Control
Eif5a	4,2732E-07	0,45620217	0,907	0,816	0,0132695	D21 vs Control
Ass1	4,8869E-07	0,52113593	0,326	0,154	0,0151753	D21 vs Control
Il7r	6,5234E-07	0,45217463	0,601	0,363	0,02025714	D21 vs Control
H3f3a	8,2198E-07	0,3708477	0,922	0,795	0,02552492	D21 vs Control
Vasp	9,3747E-07	0,42394628	0,694	0,489	0,02911125	D21 vs Control
Ncf4	9,6323E-07	0,43346567	0,306	0,137	0,02991125	D21 vs Control
Rpl7a	1,1514E-06	0,32184957	0,984	0,921	0,03575383	D21 vs Control
Esyt1	1,356E-06	0,41236533	0,58	0,368	0,04210838	D21 vs Control
Vim	1,238E-34	1,49228187	0,642	0,225	3,8442E-30	D48 vs Control
Klra9	1,2005E-31	1,16505578	0,383	0,075	3,7278E-27	D48 vs Control
Ly6c2	1,3122E-31	1,38797715	0,762	0,373	4,0748E-27	D48 vs Control
Cd7	1,5151E-31	1,17222905	0,756	0,317	4,705E-27	D48 vs Control
Capg	7,8137E-30	1,06489071	0,497	0,14	2,4264E-25	D48 vs Control
S100a6	5,0568E-24	1,20486379	0,87	0,632	1,5703E-19	D48 vs Control
Fcer1g	4,4288E-23	1,22396738	0,42	0,121	1,3753E-18	D48 vs Control
Arl4c	1,2864E-20	0,84101478	0,606	0,265	3,9946E-16	D48 vs Control
Anxa2	1,6542E-19	0,85083852	0,482	0,182	5,1369E-15	D48 vs Control
Hspa8	3,7674E-19	0,62439108	0,984	0,949	1,1699E-14	D48 vs Control
Zfp36	4,3084E-18	0,89064364	0,891	0,673	1,3379E-13	D48 vs Control
Ccl5	4,9701E-17	1,16365021	0,881	0,847	1,5434E-12	D48 vs Control
Emp3	5,7329E-17	0,87550983	0,689	0,389	1,7802E-12	D48 vs Control
Nfkbia	9,6801E-17	0,78307572	0,933	0,766	3,006E-12	D48 vs Control
Ahnak	2,1789E-15	0,76570272	0,819	0,535	6,766E-11	D48 vs Control
Crip1	2,8648E-15	0,8942054	0,922	0,79	8,8961E-11	D48 vs Control
Gzmb	6,6986E-15	1,23652655	0,617	0,347	2,0801E-10	D48 vs Control
Pim1	1,5832E-14	0,9081596	0,736	0,508	4,9164E-10	D48 vs Control
Rasgrp2	1,9461E-14	0,69276684	0,477	0,207	6,0432E-10	D48 vs Control
Lgals1	3,053E-14	0,82257255	0,813	0,627	9,4805E-10	D48 vs Control
Hsp90ab1	3,3617E-14	0,57776327	0,964	0,931	1,0439E-09	D48 vs Control
Tagln2	4,2923E-14	0,71295785	0,824	0,619	1,3329E-09	D48 vs Control
Flna	1,1864E-13	0,62117535	0,518	0,244	3,6841E-09	D48 vs Control
Atf3	1,9438E-13	0,98317396	0,316	0,113	6,036E-09	D48 vs Control
Nme2	2,6941E-12	0,61857473	0,819	0,624	8,366E-08	D48 vs Control
Klrk1	3,5364E-12	0,63690622	0,725	0,456	1,0982E-07	D48 vs Control
Actg1	5,9117E-12	0,4232041	0,984	0,977	1,8358E-07	D48 vs Control
Sidt1	7,0907E-12	0,63877507	0,311	0,118	2,2019E-07	D48 vs Control
Ass1	1,5045E-11	0,65991691	0,326	0,136	4,672E-07	D48 vs Control
Tagap	1,8291E-11	0,69058511	0,337	0,138	5,6799E-07	D48 vs Control
Il7r	2,7744E-11	0,66534698	0,601	0,345	8,6154E-07	D48 vs Control
Dynll1	3,0605E-11	0,61142191	0,632	0,375	9,5039E-07	D48 vs Control
Zyx	2,2536E-10	0,57847044	0,777	0,562	6,998E-06	D48 vs Control
Gpr183	2,7384E-10	0,51971313	0,285	0,111	8,5034E-06	D48 vs Control
Klrc2	4,0384E-10	0,52716184	0,435	0,215	1,2541E-05	D48 vs Control
Anxa1	7,4972E-10	0,46674799	0,295	0,123	2,3281E-05	D48 vs Control
Il18r1	1,0315E-09	0,50161491	0,332	0,145	3,2032E-05	D48 vs Control
Bin2	1,3044E-09	0,52041665	0,777	0,581	4,0507E-05	D48 vs Control
Nfkbiz	1,3929E-09	0,46702824	0,565	0,324	4,3253E-05	D48 vs Control
Gm26917	2,9967E-09	0,70568952	0,668	0,497	9,3056E-05	D48 vs Control
Actb	8,0146E-09	0,32906448	0,995	0,997	0,00024888	D48 vs Control
H2afz	1,0097E-08	0,56354472	0,751	0,587	0,00031355	D48 vs Control
Tgif1	1,0792E-08	0,47088293	0,342	0,161	0,00033514	D48 vs Control
Slfn2	1,1872E-08	0,47506078	0,472	0,272	0,00036867	D48 vs Control
Rpl35	1,3598E-08	0,31574392	0,974	0,981	0,00042227	D48 vs Control

Slc25a4	1,7357E-08	0,36787638	0,264	0,108	0,000539	D48 vs Control
Lgals3bp	1,7882E-08	0,45618743	0,378	0,187	0,00055529	D48 vs Control
Esyt1	1,7963E-08	0,49007565	0,58	0,361	0,0005578	D48 vs Control
Atp1b3	2,2869E-08	0,4880253	0,513	0,309	0,00071015	D48 vs Control
Slc38a2	3,3181E-08	0,485074	0,746	0,555	0,00103036	D48 vs Control
Ostf1	4,8066E-08	0,45722276	0,648	0,424	0,00149259	D48 vs Control
Tnf	8,4946E-08	0,34862065	0,259	0,11	0,00263784	D48 vs Control
Rps26	3,1075E-07	0,26088982	0,984	0,982	0,00964969	D48 vs Control
Ms4a4b	4,057E-07	0,49620845	0,86	0,737	0,01259805	D48 vs Control
Adh5	4,0584E-07	0,39040514	0,28	0,135	0,01260268	D48 vs Control
Rara	5,481E-07	0,48784136	0,264	0,124	0,01702024	D48 vs Control
Cct3	7,2412E-07	0,41030111	0,368	0,194	0,02248623	D48 vs Control
Tcf7	7,6301E-07	0,38918375	0,399	0,22	0,02369379	D48 vs Control
Ncf4	9,0669E-07	0,39590341	0,306	0,152	0,0281555	D48 vs Control
Klre1	9,7208E-07	0,47905489	0,368	0,202	0,03018589	D48 vs Control
Itgb7	1,1318E-06	0,44547478	0,705	0,535	0,03514483	D48 vs Control
Arpc2	1,3379E-06	0,35681398	0,917	0,855	0,04154733	D48 vs Control
Rpl29	1,361E-06	0,26114964	0,995	0,983	0,04226389	D48 vs Control

Supplemental Table 4. List of significant Tregs cell markers for each of the time points compared to cor

gene	p_val	avg_log2FC	pct.1	pct.2	p_val_adj	comparison
Rpl13a	2,0859E-09	1,07092635	1	0,917	6,4775E-05	D10 vs Control
Uba52	3,0608E-09	1,05005196	0,977	0,95	9,5047E-05	D10 vs Control
Vps37b	5,2245E-08	1,75605015	0,682	0,2	0,00162237	D10 vs Control
Nr4a1	2,6685E-07	1,92638658	0,75	0,333	0,00828664	D10 vs Control
Pnrc1	3,5403E-07	1,39952663	0,977	0,783	0,01099356	D10 vs Control
Csrnp1	4,3172E-07	1,70124697	0,659	0,217	0,01340635	D10 vs Control
Junb	5,817E-07	1,18808024	0,977	0,967	0,01806347	D10 vs Control
Tnfaip3	8,994E-07	1,62405626	0,886	0,6	0,0279291	D10 vs Control
Abt1	4,2162E-07	0,61273035	0,318	0,027	0,01309271	D21 vs Control
Txk	9,3742E-07	0,87983786	0,318	0,036	0,02910984	D21 vs Control
Arhgef3	8,063E-08	1,17628671	0,386	0,07	0,0025038	D48 vs Control
Emp3	3,6158E-07	0,89917913	0,909	0,611	0,01122823	D48 vs Control

ıtrol.
